# Molecular and physiological acclimation to low light and iron scarcity in a globally abundant oceanic pelagophyte

**DOI:** 10.1038/s41467-026-71628-0

**Published:** 2026-04-20

**Authors:** T. H. Coale, R. H. Lampe, M. Tan, E. Rowland, Z. Füssy, P. Venepally, H. Zheng, J. McCrow, E. M. Bertrand, A. E. Allen

**Affiliations:** 1 Ocean Sciences Department, University of California, Santa Cruz, CA USA; 2 Microbial and Environmental Genomics, J. Craig Venter Institute, La Jolla, CA USA; 3 Integrative Oceanography Division, Scripps Institution of Oceanography, University of California San Diego, La Jolla, CA USA; 4 Department of Biology, Dalhousie University, Halifax, NS Canada; 5 Faculty of Science, Department of Chemistry, University of South Bohemia, České Budějovice, CZ Czech Republic

**Keywords:** Microbial biooceanography, Marine biology, Microbial ecology, Microbial genetics

## Abstract

*Pelagomonas calceolata* is a widely distributed marine alga and is among the most numerous eukaryotes on Earth. It is abundant in subsurface chlorophyll maximum layer (SCML) communities where iron (Fe) and light are jointly scarce. To understand the strategies behind *P. calceolata*’s success, we profile this organism’s physiology and gene expression as it experiences Fe/light co-limitation. We describe cellular changes under steady-state Fe limitation and short-term responses to Fe resupply. Culture experiments reveal that *P. calceolata* maintains low Fe:carbon (C) ratios and dynamically regulates iron-sparing strategies, including flavodoxin expression and substitution of metal-rich proteins. Furthermore, environmental gene expression shows that Fe- and light-responsive genes identified in culture are enriched in SCML metatranscriptomes, indicating that *P. calceolata* expresses these adaptations in situ. These results demonstrate low Fe tolerance as a key adaptation enabling *P. calceolata* to thrive in light-limited marine environments and highlight its role in oceanic carbon and nitrogen cycling.

## Abstract (web summary)

Sunlight and iron scarcity can limit ocean algae that fuel marine food webs. This study defines iron- and light-responsive physiology, transcripts, and proteins in the abundant pelagophyte Pelagomonas calceolate and validates these responses using metatranscriptomes.

## Introduction

The rapid attenuation of sunlight in seawater restricts the vertical distribution of photoautotrophs to surface waters where nutrients are quickly depleted. This results in the formation of SCML phytoplankton communities, which straddle light and nutrient limitations. SCML communities are ubiquitous features of aquatic environments, which sometimes also represent biomass and primary production maxima^1–4^. Oceanic ferriclines are typically deeper than nitriclines^5–7^, leading to low subsurface Fe:N and the potential for iron (Fe) limitation as nutrient drawdown occurs. Fe limitation of natural SCML communities is understudied, but evidence indicates it could be widespread and intensifying^8–11^. The Fe demand of photosynthesis has the potential to result in biochemically mediated co-limitation^12^. In response to low irradiance, algae can increase their cellular inventories of both photosynthetic pigments^13^ and photosystem proteins^14^. This entails an increased Fe requirement; therefore, the low light of SCML habitats has the potential to exacerbate Fe limitation^15^. Phytoplankton that successfully inhabit these environments are adapted to endure despite the scarcity of these two essential components of photosynthesis.

After the Pelagophyceae (Stramenopiles) class was established^16^, environmental surveys confirmed the presence of this lineage in the Atlantic and Pacific Oceans via diagnostic pigments^17,18^. Subsequently, *P. calceolata* has been observed worldwide in many marine metagenomic surveys^19–21^. Other studies have identified *P. calceolata* as a significant member of SCML phytoplankton communities found in both the Northeast and Southeast East subtropical Pacific^8,22–24^. Global-scale marine metagenomics found pelagophytes to be one of the principal photosynthetic groups in the world’s oceans^25^, and *P. calceolata* was found to occupy an ecological niche characterized by low Fe and low light availability^26,27^.

Fe is required by marine phytoplankton in varying amounts reflecting adaptation to Fe bioavailability^28–30^. *P. calceolata* has among the lowest Fe requirements for growth of any eukaryotic phytoplankton yet studied in culture^28,31^, which affords it success in Fe-poor environments. Analysis of gene expression across high and low-Fe oceanic environments has provided broad insight into the underlying cellular response of *P. calceolata* to Fe bioavailability^27^. The *P. calceolata* genome contains genes that are Fe sensitive across phytoplankton taxa, such as the iron-starvation-induced proteins (ISIPs), ferric reductases, multi-copper oxidases, plastocyanin, flavodoxin, and phytotransferrin (*pTF*)^27^. However, many Fe-related genes exhibit a diel expression pattern, and Fe-stress-related changes to central metabolic processes are differentially expressed across the day/night cycle^32,33^. Phototrophic organisms modify their transcriptomes in tight synchrony with light availability^34,35^, and when photosynthesis is impacted by Fe stress, the timing of these changes likely becomes more important. In the case of *P. calceolata*, these regulatory patterns may play an outsized role due to its particular ecological niche.

The elemental stoichiometry of phytoplankton biomass can vary between cells experiencing different physicochemical environments, and Fe availability can be a major factor. Cellular Fe quotas can fluctuate due to luxury uptake and storage, or substitution of low Fe alternative proteins. This dynamic adjustment of Fe content mirrors the patterns in gene expression described above, indicating that variability in Fe quotas may be closely coordinated with the regulation of Fe acquisition and utilization pathways. Laboratory studies of marine phytoplankton have demonstrated increased Fe quotas at low light intensities^29,36^ and short photoperiods^37^. Fe also exerts influence on the stoichiometry of other elements through its role in C and nitrogen (N) metabolism. Fe limitation has been shown to alter nutrient uptake, as well as C fixation rates in diatoms and phytoplankton communities^38,39^. The influence of Fe/light co-limitation on the elemental composition of *P. calceolata* holds significant implications for its biogeochemical role, yet remains understudied.

**Fig. 1:**
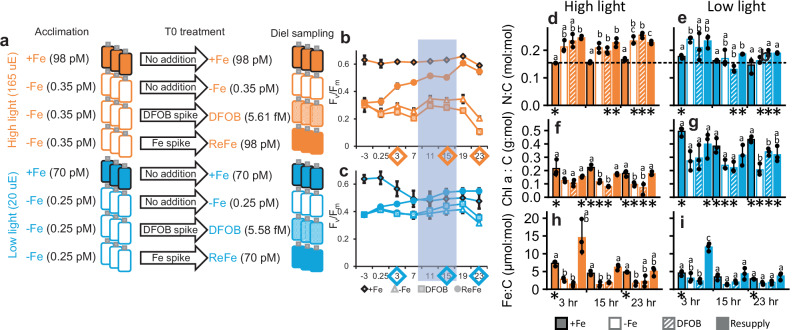
Fe/light co-limitation experiment design and time-resolved physiological responses in *P. calceolata.* **a** Experimental design of Fe/light co-limitation experiment. Triplicate cultures were acclimated to initial conditions, and the experiment was initiated at T0 with chelator (DFOB) and Fe additions to certain cultures. Calculated average Feʹ concentrations given in parentheses after Fe treatment. Sampling then occurred over the ensuing 23 hours. Fv/Fm of *P. calceolata* cultures over the diel cycle are shown for high light (**b**), and low light (**c**), experiments. Hours since spike given on x-axis. Fe resupply and DFOB spike occurred at 0 hours. The shaded area indicates the dark period of a 14:10 h light:dark cycle. Major time points for physiological characterization are indicated with diamonds. Ratios of nitrogen (**d**, **e**), chlorophyll *a* (**f**, **g**), and Fe (**h**, **i**) to carbon (C) at the three major time points. High light treatments are in orange, low light in blue. Bars show the mean of biological triplicate (n = 3 per treatment) cultures with error bars representing ± 1 SD. Letters above bars denote groups that are not significantly different from each other, based on two-sided pairwise comparisons within the same timepoint and light level (Holm-adjusted *P* < 0.05), Asterisks (*) indicate significant differences between the light levels at the same timepoint and Fe treatment (two-sided Welch’s t-test, *P* < 0.05). Exact *p*-values are given in a Source Data file. Dotted line (**d**, **e**) indicates a ratio of 16 N:106 C. Source data are provided as a Source Data file.

To characterize the physiological strategies *P. calceolata* employs under Fe/light co-limitation in the marine environment, we conduct diel sampling across light and dark transitions to capture time-resolved cellular responses (Fig. 1a). We measure cellular content of C, N, and Fe and documented how these change in response to Fe bioavailability and light levels. We also profile the transcriptome and proteome of *P. calceolata* to examine the impact of Fe and light limitations, Fe/light co-limitation, release from Fe limitation and short-term changes in Fe speciation. We utilize our findings to quantify *P. calceolata*’s contribution to primary productivity in hundreds of environmental samples spanning a range of conditions. This study supports the emerging viewpoint *P. calceolata* is a key marine eukaryote with versatile metabolic strategies and a significant role in carbon cycling.

## Results

### Fe and light induce state changes in *P. calceolata* physiology

To investigate how *P. calceolata* acclimates to combined Fe and light limitation, we conducted an experiment manipulating Fe availability and irradiance under controlled trace-metal conditions. Cultures were acclimated for more than ten generations in Aquil medium with defined Fe′ concentrations before sampling (Supplementary Fig. 1a, b). Two light intensities were used to bracket natural SCML conditions: high light (HL, 165 µmol quanta m^−2^ s^−1^) and low light (LL, 20 µmol quanta m^−2^ s^−1^). Each light treatment was crossed with two iron regimes: Fe-replete (+Fe, 98–70 pM Feʹ) and Fe-limited (−Fe, 0.35-0.25 pM Feʹ), yielding four primary treatments (HL +Fe, HL -Fe, LL +Fe, LL -Fe) with triplicate biological cultures. After long-term acclimation, we initiated diel time-course sampling every 4 hours for physiological measurements, elemental quotas, transcriptomics, and TMT-based proteomics, followed by short-term Fe-resupply (ReFe) and siderophore (desferrioxamine b, DFOB) perturbations (Fig. 1a). This design allowed us to disentangle main and interactive effects of Fe and light while capturing rapid and diel responses across cellular, molecular, and biochemical scales. Fe availability had consequences for *P. calceolata* cells at both light levels. Fe-starved *P. calceolata* is photosynthetically impaired as indicated by low Fv/Fm ratios (Fig. 1b, c, Supplementary Data 1). Photosynthetic competency was highest in the Fe-replete cultures, and the Fe-resupply cultures showed a steady increase in Fv/Fm over the experiment. By the final time point, 23 hrs after the Fe addition, both resupply treatments had recovered Fv/Fm.

Fe-starved cells had a lower C content than Fe-replete cells at both high and low light (Fig. 1c, d). Co-limited cells had the lowest C quotas. N quotas showed the same trend in the low light cultures, but no difference was observed at high light (Supplementary Fig. 1e,f). This results in high light Fe limited cells having the largest departure from a Redfield-like stoichiometry^40^ due to a lack of C (Fig. 1d, e). Co-limited cells had the lowest N content, although this recovered following Fe resupply (Supplementary Fig. 1e, f). Differences between Fe-deplete and chelator-treated cells were not evident in any of these physiological measurements.

Chlorophyll *a* (Chl *a*) quotas were higher in all the low light-adapted cells than in any of the high light cultures (Supplementary Fig. 1g,h). Chl *a*:Fe ratios were lower in the high light cultures (Supplementary Fig. 2a,b), which is consistent with previous findings^29^. In both light treatments, the Fe resupply cultures had the lowest Chl *a*:Fe, likely resulting from rapid Fe uptake and slower Chl *a* biosynthesis.

Fe quotas were highest in the Fe replete cultures and similar in the resupply cultures (Supplementary Fig. 1i, j). Despite the high C quota of Fe-replete cells, the Fe:C ratio was still higher in these relative to the Fe-limited cells, especially at high light (Fig. 1h, i). Fe resupply cultures after 3 hrs had the highest Fe:C due to low initial C quotas, and high Fe uptake rates subsequent to the resupply spike (Fig. 1h, i).

Quotas of copper (Cu) followed a trend opposite to that of Fe quotas. Fe-limited cells had higher quotas for Cu (Supplementary Fig. 2c, d). This was also the case for Cu:C ratios, which were higher in the Fe-limited cultures (Supplementary Fig. 2e, f). Cu (both Cu/cell and Cu:C) in resupply cultures at both light levels began high but returned to values seen in the Fe replete cultures by the end of the experiment.

**Fig. 2:**
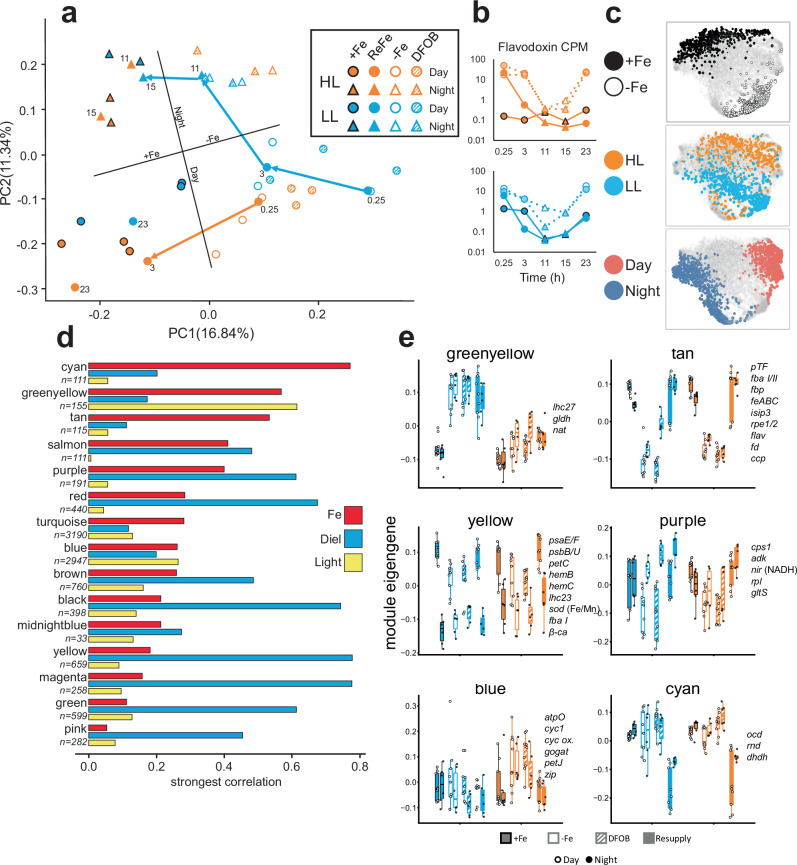
Transcriptome dynamics under Fe/light co-limitation across the diel cycle. **a** Principal component analysis plot of *P. calceolata* transcriptomes. Each symbol represents biological triplicates at a single time point. Orange and blue indicate high light (HL) and low light (LL), respectively. Triangles indicate daytime points and circles show nighttime points. Black outlines indicate iron replete (+Fe), solid fill with no outline indicates iron resupply (ReFe), no fill indicates iron deplete (−Fe), and diagonal stripes indicate DFOB. ReFe points are labeled with hours since iron spike. **b** Flavodoxin transcript abundance (CPM) over the diel cycle. Symbols show mean CPM of biological triplicates. **c** Manifold approximation and projection of *P. calceolata* gene expression in 120 transcriptomes across the diel cycle and in response to iron/light co-limitation. Each circle represents a gene, with size scaled to average logCPM. Differentially expressed genes are colored according to response type determined with edgeR. **d** Bars show the strongest correlation between each module eigengene and Fe-treatments, diel patterns, or light level. Diel strength reflects the maximum correlation with sin24, cos24, or day/night encoding of the sampling time. Fe strength reflects the maximum correlation with any of the four iron treatments (+Fe, −Fe, DFOB, ReFe). *n* indicates the number of genes included in each module. **e** Each panel shows WGCNA module eigengene values under low and high light. Within each light treatment, boxes denote Fe treatments; dots are biological replicates (*n* = 3 independent cultures per treatment, 3-day and 2-night timepoints) plotted as day or night means per culture. Box plots show the median, IQR (25th–75th), and whiskers to 1.5×IQR (outliers not shown). Selected gene annotations are listed next to each panel. Source data are provided as a Source Data file.

Transcriptomes of each treatment were sequenced at five time points (15 min, 3 hr, 11 hr, 15 hr, and 23 hr) after resupplying with Fe or spiking the strong chelator, DFOB. The impact of our culturing conditions was evident on a global transcriptomic scale. There was clear differentiation between day and night samples, and by Fe treatment (Fig. 2, Supplementary Data 2–6). Fe-deplete and DFOB treatments were closely clustered, and separated from Fe replete samples (Fig. 2a). Fe resupply samples began associated with the low Fe treatments, but later time points were associated with the Fe replete treatment (Fig. 2a). In the low light treatment, this shift occurred by the 15 hr time point while in the high light treatment, all resupply points beginning at 3 hrs appeared similar to Fe replete samples. Many genes responded in a similar way to both Fe and light, likely due to similar effects of each on photosynthetic activity (Fig. 2b, Supplementary Fig. 3a–c).

Weighted gene co-expression network analysis (WGCNA) revealed sets of genes whose expression patterns responded distinctly to Fe status, light intensity, and diel phase (Fig. 2c, Supplementary Figs. 4, 5). Several modules were strongly diel, including the yellow module, whose diel fluctuations became attenuated under high light, indicating light-dependent modulation of diel rhythms (Fig. 2d). Additional modules were co-regulated by both diel phase and Fe status. In contrast, only two modules (greenyellow and blue) showed strong light responses, and both were also Fe sensitive; one module integrated all three regulatory inputs (Fig. 2d, e). A separate module showed a strong Fe response without sensitivity to light or diel variables, indicating a core Fe-responsive network (tan module, Fig. 2d, Supplementary Data 7). Together, these module-level patterns demonstrate that Fe and light regulate broad, coordinated gene networks in *P. calceolata*, and that environmental stress can reshape the timing or strength of daily transcriptional rhythms.

**Fig. 3:**
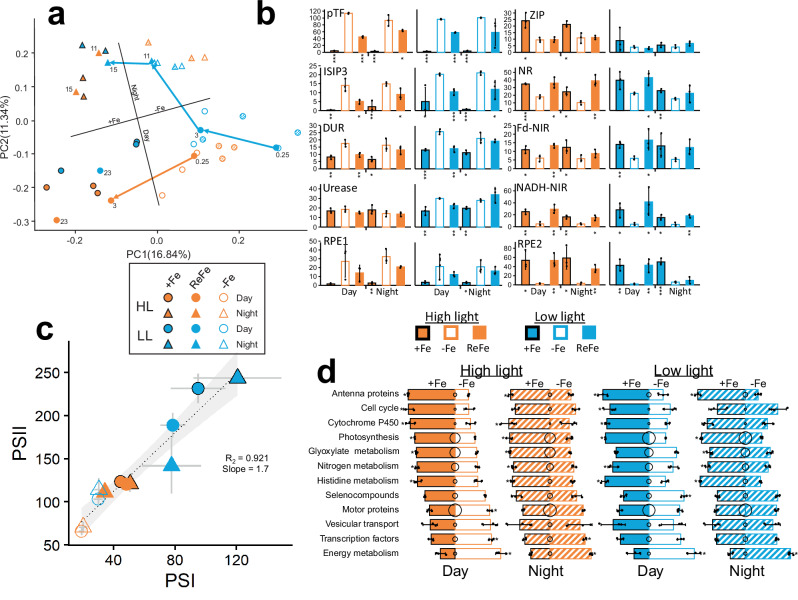
Proteomic response of *P. calceolata* to Fe and light. **a** Principal component analysis plot of *P. calceolata* proteomes (TMM of 2924 quantified proteins). Each symbol represents a single sample. Orange and blue indicate high light (HL) and low light (LL), respectively. Triangles indicate daytime points and circles show nighttime points. Black outlines indicate iron replete (+Fe), solid fill with no outline indicates iron resupply (ReFe), and no fill indicates iron deplete (−Fe). ReFe points are labeled with hrs since the iron spike. **b** Scaled day and night abundance of certain iron-sensitive proteins. For each protein, bars show the mean normalized abundance (mean ± SD) for Day and Night samples under high light (HL) and low light (LL) (facets). Individual biological replicates (*n* = 3) are overlaid as black points. Three iron conditions are shown (+Fe, −Fe, ReFe), with −Fe displayed as an open bar and +Fe/ReFe as filled bars. Vertical asterisks indicate significant differences relative to the −Fe reference within the same light regime and time period (*=*p* < 0.05, **=<0.01, ***=<0.001, two-sided unpaired t-tests, equal variance) difference from the −Fe value at the same time of day. Exact *p*-values and protein annotations are given in a Source Data file. **c** Sum of protein abundances for all detected PSI and PSII subunits in proteomic samples. Error bars represent +/− 1 SD, and the dotted line shows linear regression with 95% confidence interval shown in light grey. **d** Bars show the mean percent allocation of KEGG Orthology (KO) pathway-associated protein abundance to +Fe versus −Fe within each pathway and panel (HL Day, HL Night, LL Day, LL Night). Allocation is computed per biological replicate so that (+Fe + −Fe) = 100% within each pathway and panel. Black points show replicate-level allocations (*n* = 3 independent cultures); error bars show mean ± 1 SD across replicates. Circles at zero scale with mean total pathway abundance. Asterisks denote pathways with significant +Fe vs −Fe differences by limma (BH-adjusted FDR < 0.05) within the corresponding panel, and exact p values and other source data are provided in a Source Data file.

Proteins were analyzed from day (0 or 23 hrs) and night (11 hrs) time points for replete, deplete, and resupply cultures at both light levels. Resupply cultures were sampled 11 (night) and 23 (day) hrs after the spike; therefore, the night sample was the earliest after the Fe addition, and Fe deplete conditions. Fe availability resulted in similar patterns of proteome remodeling at high and low light. Time of day had little impact on proteomic profiles (Fig. 3a, Supplementary Fig. 6); the major differences were between Fe and light levels (Fig. 3a, Supplementary Figs. 3d, 7, 8). Nighttime samples from the low light resupply were more similar to Fe-deplete samples, while high light resupply had recovered to near Fe replete by 11 hrs post-spike (Fig. 3a). High and low light proteomes showed the greatest similarity at low Fe. Overall, proteomes appear less dynamic than transcriptomes and show less variation by time of day, with 43.9% of transcripts differentially expressed at day or night (Fig. 3a) as compared to <1% of proteins (Supplementary Fig. 6, Supplementary Data 9). To ask whether co-limitation exceeds the sum of single limitations, we estimated the Fe × Light interaction for each measured characteristic (physiology, RNA, and proteomics) and estimated the excess over additivity of the co-limited state. A positive interaction indicates synergy (co-limitation stronger than additive); a negative interaction indicates antagonism/buffering (co-limitation weaker than additive); values near zero indicate additivity (Supplementary Table 1, Supplementary Data 10–12). Across RNA, and proteins, most traits/genes/proteins showed no significant interaction (RNA 98.5%, proteins 98.4%), and among the features with significant interactions, negative terms outnumbered positive ones (RNA 110 vs 32; proteins 32 vs 10). By contrast, physiology showed a higher fraction of interaction effects (34% significant overall; 6 positive, 4 negative), consistent with whole-cell metrics integrating many small changes across RNA and protein. Nevertheless, the modest magnitudes and mixed signs at the trait level indicate that Fe/light co-limitation is generally buffered rather than synergistically amplified in *P. calceolata*.

### Balancing Fe and energetic demands during colimitation

**Fig. 4:**
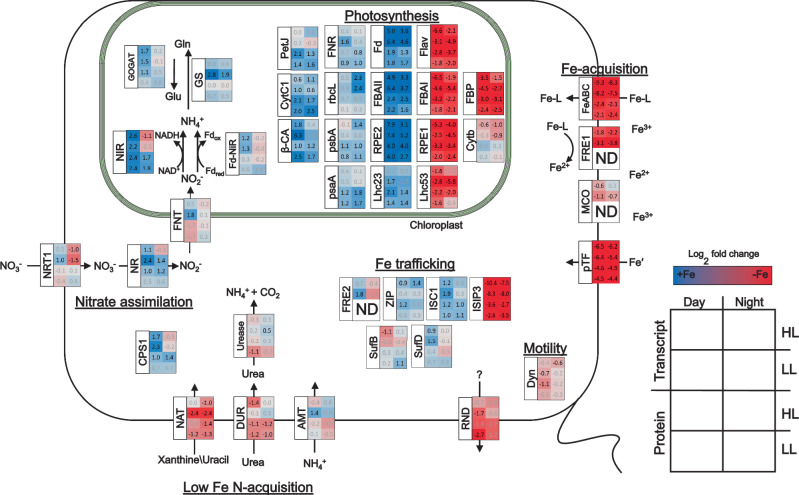
Iron- and light-induced state changes in the cellular functioning of *P.calceolata*. Cellular diagram summarizing impacts of light and Fe on key processes. Blue indicates higher abundance in Fe-replete cultures and red indicates higher abundance in low-Fe cultures; heatmaps show log2 fold change. Transcript differential expression was tested in edgeR using a negative binomial generalized linear model with likelihood ratio tests (two-sided); protein differential abundance was tested in limma using linear models with empirical Bayes moderation (two-sided). P values were adjusted for multiple comparisons using the Benjamini–Hochberg procedure; features with FDR < 0.05 are indicated in black text. AMT ammonia transporter, β-CA beta carbonic anhydrase, CPS1 carbamoyl-phosphate synthetase, Cytb cytochrome b, CytC1 cytochrome C1, DUR urea transporter, DYN dynein, FBA fructose-bisphosphate aldolase, FBP fructose 1,6-bisphosphatase, Fd erredoxin, Fd-NIR ferredoxin nitrite reductase, FeABC ferrichrome ABC transporter protein, Fe-L Fe bound to organic ligand, Flav flavodoxin, FNT formate/nitrite transporter, FRE ferric reductase, GOGAT gluatmate synthase, GS glutamine synthetase, ISIP3 iron starvation induced protein 3, Lhc light harvesting complex protein, MCO multicopper oxidase, NAT nucleobase-ascorbate transporter, NCS2 uracil-xanthine permease, NIR NADPH nitrite reductase, NRT1 nitrate transporter, PetJ cytochrome C6, psaA photosystem I reaction center protein A, psbA photosystem II reaction center protein A, pTF phytotransferrin, rbcL ribulose 1,5 bisphosphate carboxylase large subunit, rbcS ribulose 1,5 bisphosphate carboxylase small subunit, RND resistance-nodulation-division family transporter, RPE ribulose 5-phosphate 3-epimerase, SufB Fe-S cluster assembly protein B, SufD Fe-S cluster assembly protein D, ZIP zinc responsive transporter/iron responsive transporter-like protein. Source data are provided as a Source Data file.

Many of the Fe stress biomarkers known from other phytoplankton were present and Fe-responsive in *P. calceolata* transcriptomes. Transcript abundance for flavodoxin shows a pattern of up-regulation in all low Fe cultures (Fig. 2b, Supplementary Fig. 9), which provides evidence that Fe concentrations were low enough to induce Fe-sparing in both the low Fe and the chelator addition treatments. Flavodoxin transcript expression shows a diel fluctuation, and is only highly expressed in the daytime points of low Fe cultures (Fig. 2b, Supplementary Fig. 9). Expression was down-regulated within 15 minutes of Fe addition, and reached Fe replete levels by 3 hours. The flavodoxin protein lacked diel variation, was more abundant in low Fe treatments, and significantly decreased in abundance within the course of the experiment only in the high light treatment (Fig. 4, Supplementary Fig. 10). Two ferredoxin genes exhibited contrasting responses with respect to Fe (Supplementary Fig. 9). One ferredoxin gene, which was predicted to localize to the chloroplast, achieved maximum transcript abundance in high Fe cultures, did not display a diel pattern, and was upregulated by 3 hours after the Fe spike at both light levels. The other protein with a ferredoxin domain was expressed at low Fe and downregulated by the 15 minute time point at both Fe levels, but was not detected at the protein level. At both light levels, ferredoxin protein expression in high Fe cultures was replaced by flavodoxin in low Fe, and the reverse was observed following resupply (Fig. 4, Supplementary Fig. 10). Another well-characterized low Fe substitution is the Cu-containing plastocyanin for Fe-containing cytochrome c_6_^41^. In *P. calceolata*, this substitution was not detected, despite abundant copper (40 nM) in the culture media. Both plastocyanin and cytochrome c_6_ were upregulated in Fe replete cultures at both light levels without a diel pattern (Supplementary Fig. 9). Cytochrome c_6_ was detected in our proteomics dataset and, like its transcript, was expressed at high Fe (Fig. 4, Supplementary Fig. 10). When growth is limited by one essential nutrient, quotas of non-limiting nutrients may increase^42^, but high Cu quotas in Fe-limited cultures could further be explained by increased reliance on Cu-containing proteins or by transport mechanisms either poorly discriminate between metal cations or are co-regulated by a shared signaling pathway. Upregulation of transport mechanisms at low Fe could result in the accumulation of other metals, especially in media replete with nutrient metals. Additionally, if detoxification is likewise nonspecific, Fe-limited cells may repress export mechanisms to avoid losing Fe. Fe-limited diatoms have been shown to be more susceptible to copper toxicity^43^, and detoxifying phytochelatins are known to chelate a variety of transition metals^44^. ISIP3, a poorly characterized Fe-related protein^45^, was also overexpressed at low Fe (Figs. 3b, 4, Supplementary Fig. 9). ISIP3 contains the ferritin-related DUF305 domain, but also a cupredoxin/plastocyanin-like domain and may be related to increased Cu quotas in Fe-limited cells.

Many transporters in the *P. calceolata* genome are Fe sensitive. Notably, *pTF* is strongly expressed across the diel cycle in Fe-limited cultures only (Fig. 4, Supplementary Fig. 9), representing a strategy for acquiring dissolved inorganic Fe^46^. This pattern is also reflected in pTF protein abundances (Figs. 3b, 4). *P. calceolata* may also acquire organically complexed Fe using a siderophore receptor. BLASTP indicates significant similarity to the ferrichrome binding proteins of diatoms proteins (E = 9 × 10^−13^; 28% identity, 43% positives, 15% gaps over 258 aa, Supplementary Fig. 12d)^47^. This protein was expressed in low Fe cultures and decreased when Fe was added, and may explain growth in the presence of DFOB, which induced extremely low free Fe levels (Fig. 4, Supplementary Fig. 11). Uptake assays confirmed that *P. calceolata* can acquire DFOB-chelated Fe, and Fe-limited cells showed higher uptake rates (Supplementary Fig. 12). Transcripts for zinc regulated transporter/Fe regulated transporter-family proteins (ZIP) were expressed in all cultures potentially indicating a role in intracellular trafficking of Fe^48^ but not specific to low Fe conditions (Fig. 4, Supplementary Fig. 9). ZIP proteins were upregulated in Fe-replete cultures implying functions other than Fe uptake (Figs. 3b, 4, Supplementary Fig. 11). Finally, transporters of the resistance-nodulation-division (RND) family were upregulated in low Fe cultures (Fig. 4, Supplementary Figs. 9, 11). RND transporters are large proteins that pump a variety of substrates across the outer membrane in bacteria^49,50^ and are also involved in the efflux of heavy metals^51^. It could be that these transporters are involved in the efflux of excess non-Fe metal ions acquired through non-specific metal uptake in media replete with other nutrient metals, but this remains a hypothesis until future work directly characterizes these proteins’ substrates and activities.

Mitochondrial genes were impacted by Fe limitation and light limitation, and were slow to respond to Fe resupply. NADH-ubiquinone oxidoreductase subunits of mitochondrial complex I were almost highly expressed in the high-light Fe-replete condition and showed recovery in Fe resupply treatments (Supplementary Fig. 9). The same was true of cytochrome c oxidases predicted to be localized to the mitochondrion. Cytochrome P450 proteins were also more abundant during the day in high Fe cultures (Fig. 3d).

Previously, a large portion of dynein transcripts in marine ecosystems has been attributed to pelagophytes^25^. At high light, low Fe cultures had the greatest dynein transcript expression, and Fe resupply reduced their expression to the levels seen in Fe-replete cultures (Fig. 4, Supplementary Fig. 13, Supplementary Data 13). At low light, all cultures showed a similar degree of dynein expression, except Fe resupply, which downregulated these genes (Supplementary Fig. 13). Most dynein genes expressed at low Fe were classified as axonemal heavy chain molecules^52^ (Supplementary Data 13). The function of these Fe-sensitive dyneins is highly coordinated with the light:dark cycle. The other major groups of motor proteins, kinesin and myosin, were similarly expressed at night but less likely to be expressed at low Fe (Supplementary Fig. 13). Dynein proteins were also detected and showed upregulation at low Fe in high light, and little sensitivity to Fe concentration at low light (Supplementary Fig. 10). These results indicate that dynein serves a distinct role at low light and/or low Fe that is not shared with other motor proteins. More broadly, these molecular responses demonstrate capacity for stringent Fe economy, yet comparative fitness under co-limitation relative to co-occurring taxa remains to be established and will require direct interspecific competition experiments under controlled Fe/light regimes.

### Impacts on primary C metabolism

RuBisCO transcript abundance was coordinated with daylight, and was highest in the Fe-replete cultures (Fig. 4, Supplementary Fig. 9). Fe resupply only marginally increased RuBisCO expression, and mostly in the low light cultures. Other Calvin-Benson cycle genes responded to Fe. Class I fructose bisphosphate aldolase (*Fba*) was preferentially expressed in low Fe day samples, while multiple class II Fbas were expressed at high light during the day (Fig. 4, Supplementary Fig. 9). Resupply cultures largely made the switch in gene expression from class I to class II within 3 hours (Supplementary Fig. 9). One fructose 1,6-bisphosphatase (*Fbp1*) gene expressed during the night was also mostly expressed at high Fe (Fig. 4, Supplementary Fig. 9). Another copy of this gene was expressed during the day at low Fe and quickly downregulated following Fe resupply. Similarly with ribose 5-phosphate 3-epimerase (Rpe), different copies were expressed at replete and deplete Fe levels, though both mostly during the day (Fig. 3b, 4, Supplementary Fig. 9). These photosynthetic proteins, which are upregulated at low Fe, are components of the regenerative phase of the Calvin-Benson cycle. Class I Fba proteins are expressed in many Fe-limited phytoplankton species, including cyanobacteria^53,54^, but the metabolic function of this is still unknown. It may be a substitution for class II Fbas, which are upregulated at high Fe and require a metal cofactor, but this is thought to be zinc^55^. FBP is not known to be overexpressed in Fe-starved diatoms, yet it is one of the most Fe-sensitive proteins in *P. calceolata* (Supplementary Fig. 7). Transcripts for the low Fe-expressed Fbp (*Fbp2*) peak during the day, which contrasts with the night-expressed Fbp in *P. tricornutum*^32^. At night in *P. calceolata*, a second Fbp (*Fbp1*) peaks in expression as it does in *P. tricornutum* (Supplementary Fig. 10). Rpe is a metalloenzyme that binds two divalent metal cofactors and is a component of both the Calvin-Benson cycle and the pentose phosphate metabolic pathway^56^. Both homologs are expressed during the day, but Rpe1 at low Fe and Rpe2 at high Fe (Figs. 3b, 4, Supplementary Fig. 9). The transcription of low-Fe Fba, Fbp, and Rpe is temporally coordinated (Fig. 2e, Supplementary Fig. 9), which is evidence that they are working together regenerating ribulose 1,5-bisphosphate for RuBisCO. Expression patterns of all these proteins follow similar patterns but with less variation by time of day, and slower changes after Fe addition (Fig. 2e, Supplementary Fig. 10). Most other genes associated with the Calvin-Benson cycle were primarily expressed in Fe-replete and resupply treatments at both light levels (Fig. 4, Supplementary Fig. 9).

Major photosynthetic proteins showed clear patterns relating to Fe and light availability (Fig. 3d, Supplementary Fig. 14). RuBisCO is most highly expressed when both Fe and light are available and at high light Fe-limitation results in much lower expression, while at low light Fe limitation did not impact expression (Fig. 4, Supplementary Fig. 14). No variation in rbcL abundance was observed between day and night samples and rbcL protein abundances recovered by the 11 hour time point (Fig. 4, Supplementary Fig. 14). Reaction center subunits from PSI and PSII were much more abundant in low light cultures when Fe was present (Fig. 3c, Supplementary Fig. 14). Despite differences in PSI and PSII quantity, the ratio of these proteins did not change with different treatments (Fig. 3c).

### Fe and light impacts on N source preference

Nitrogen metabolism was also impacted by Fe concentration, but unlike C, the impact on cellular N quotas was only evident in cells experiencing iron/light co-limitation (Supplementary Fig. 1e, f). At high light, high Fe and resupply cultures upregulated transcription of many components of the nitrogen assimilation pathway (Fig. 4, Supplementary Fig. 9). Fe resupply triggered gene expression of both ferredoxin and NADPH-dependent nitrite reductases, nitrite and nitrate transporters, urea and ammonia transporters, and urease. Glutamine synthetase (GS) was induced by Fe addition, as were carbamoyl phosphate synthetases (CPS) and glutamine oxoglutarate aminotransferases (GOGAT, Supplementary Fig. 9). Most nitrogen compound transporters were expressed primarily at night, while assimilation genes were expressed in the day or constitutively (Supplementary Fig. 9). These findings suggest low Fe induced impairment of nitrate uptake and assimilation, and potentially the urea cycle. The influence of Fe availability was also apparent in inventories of proteins related to nitrogen uptake and assimilation (Fig. 4, Supplementary Fig. 15). Nitrate and nitrite reductases were expressed primarily in high Fe cultures (Figs. 3b, 4, Supplementary Fig. 15). Urea transport was significantly overexpressed in low Fe cultures and decreased after Fe was added (Figs. 3b, 4). Urease showed the same pattern but only at low light (Fig. 3b). Nitrogen assimilation proteins GS and GOGAT were repressed in low Fe cultures at both light levels (Fig. 4), and Fe replenishment resulted in recovery of these proteins by the end of the experiment (Supplementary Fig. 15).

Diatom nitrogen metabolism is largely nitrate fueled^57–59^, which also appears to be the case for *P. calceolata* when Fe is available. Nitrate transport and assimilation in the chloroplast are upregulated regardless of light availability when bioavailable Fe is present. Diatoms also possess an ornithine-urea cycle, which is considered a central C and N hub that links catabolic and anabolic metabolism by efficiently recycling ammonium and bicarbonate derived from protein turnover^60^. *P. calceolata* contains urea cycle enzymes^61^ (Fig. 4), affording it the same metabolic flexibility, which is thought to confer a competitive advantage during episodic nutrient input^60^. Diatoms maintain expression of urea cycle proteins at low Fe^32,62^, while in *P. calceolata* the urea cycle is preferentially expressed at high Fe alongside nitrate assimilation (Fig. 4). At low Fe, *P. calceolata* utilizes other strategies to minimize Fe costs, including exploitation of reduced and/or organic nitrogen sources. Reduction of nitrate to ammonia requires two Fe-containing enzymes^63^, and significant ATP and NAD(P)H^64^. This investment of Fe could be circumvented via acquisition of ammonium, urea, amino acids, and purines. *P. calceolata* contains transporters for all these substrates, and in our cultures, urea uptake and assimilation were upregulated in Fe-deplete conditions (Fig. 4). Diatoms require more Fe when grown on nitrate than when grown on ammonium, presumably due to Fe-containing nitrate and nitrite reductases^65^ which *P. calceolata* downregulates during Fe-limitation. Together with SCML metatranscriptomes showing enrichment of these pathways, our results support (but do not quantify) *P. calceolata*’s contribution to nitrate-supported production; species-resolved uptake rates in situ remain a target for future work.

### *P. calceolata* in the marine environment

**Fig. 5:**
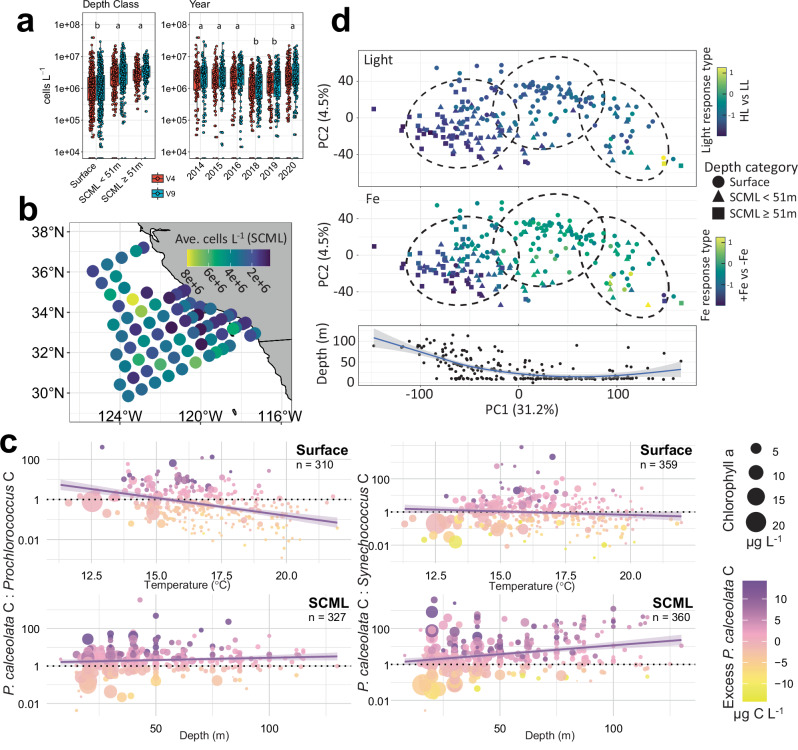
Environmental abundance, carbon contribution, and in situ expression signatures of *P. calceolata* in the California Current ecosystem. **a** Cells per L of *P. calceolata* as determined by abundance of V4 and V9 regions of the 18 s rRNA gene in 806 samples from the CCE, shown according to depth category (surface, deep and shallow SCML) and by year. Box plots show the median (center line), interquartile range (box; 25th–75th percentiles), and whiskers extending to 1.5 × IQR; points are individual samples. Letters denote significant difference among means (*p* < 0.05, Kruskal-Wallis Test). **b** Average concentration of *P. calceolata* cells in the CCE across the NCOG dataset as determined by abundance of the V9 region of the 18 s rRNA gene. **c** Comparison of contributions to POC by *P. calceolata, Prochlorococcus* and *Synechococcus* in surface and SCML samples across the CCE. Ratios of estimated C per L are given on the vertical axis, and points are colored by the difference between estimated C in µg C L^-1^. Point size corresponds to total chlorophyll *a* concentration. A generalized linear model with 95% confidence interval around the fitted mean is shown with the purple line and shading. **d** PCA was constructed using the expression of all *P. calceolata* genes in NCOG metatranscriptomes. Points colored by ratios of transcript abundance of genes categorized by response type. Depth of the samples along PC1 is shown below. A generalized linear model with 95% confidence interval around the fitted mean is shown with the blue line and shading. Source data are provided as a Source Data file.

We quantified *P. calceolata* cell abundance in the California Current Ecosystem (CCE) using both the V4 and V9 regions of the 18S rRNA gene and estimated two 18S copies per cell. These sequences are derived from the NOAA-CalCOFI Ocean Genomics (NCOG^66,67^) dataset spanning 6 years, multiple depths, and physicochemical environments. These results show *P. calceolata* to be a ubiquitous phytoplankton species averaging ~2 million cells per liter in surface waters and ~3.7 million in SCML communities (Fig. 5a, b, Supplementary Data 14). Higher abundances were observed from 2014–2016 and 2020, corresponding to warmer periods including an unprecedented heatwave^68–70^ associated with El Niño (Fig. 5a), and the 2014-2016 period saw on average 16% more cells in surface waters and 39% more in SCML communities (Fig. 5a, Supplementary Data 14). Using our measurements of cellular C, we estimate the contribution of *P. calceolata* to total particulate organic C (POC) in the CCE and compare this to the cyanobacteria *Prochlorococcus* and *Synechococcus. P. calceolata* frequently accounts for more C than either, especially in cooler surface water (Fig. 5c). We performed a sensitivity analysis (Supplementary Fig. 16) that recomputes *P. calceolata*:cyanobacteria carbon ratios across a range of cellular C quotas and plausible ploidy (1–8 genomes cell^−1^; two 18S copies genome^−1^). Despite variation (especially with ploidy), the conclusion that *P. calceolata* is a major POC contributor, particularly in SCMLs, is consistently supported. We also surveyed the gene expression of *P. calceolata* in the NCOG dataset (*n* = 220) and found genes that corresponded to low Fe and low light within our experiment to be overrepresented in SCML samples, supporting that our experiment characterized environmentally relevant responses to light and Fe (Fig. 5d). While the patterns above are consistent with success under Fe/light limitation typical of SCMLs, we do not ascribe *P. calceolata’s* prevalence to Fe economy alone. Additional traits likely contribute, including very small cell size and high surface-area:volume ratios that favor resource acquisition^71^. Other processes, such as access to organic nutrient pools and trophic dynamics (e.g., grazing susceptibility), could also play roles, though we do not evaluate them directly here.

## Discussion

A collection of known low Fe strategies enables *P. calceolata* to grow at bioavailable Fe concentrations substantially lower than those found in even the most Fe-depleted marine environments^72^. Many proteins used by *P. calceolata* under low Fe conditions (such as flavodoxin, pTF, and ISIP3) are also found in diatoms and serve roles in Fe-sparing, Fe acquisition, and intracellular Fe trafficking^32,48,73^. Fe acquisition from siderophores occurs through an independent uptake pathway. Small cell size further benefits *P. calceolata* by minimizing Fe quotas and increasing surface area-to-volume ratios, traits predicted to confer a selective advantage under Fe/light co-limitation^29,74,75^. *P. calceolata* is among the smallest of eukaryotes, and its biomass decreases substantially under Fe limitation (Supplementary Fig. 1c, d), further reducing Fe demand. Light-reaction proteins in *P. calceolata* showed no diel oscillation in abundance, suggesting an absence of detectable Fe recycling between daytime and nighttime processes^76^. However, we cannot exclude the possibility that Fe exchange occurs on timescales or in protein pools not captured by our sampling, or that Fe cofactors are exchanged without reduction in protein inventories. While Fe quotas closely track bioavailability in culture, Fe:C ratios remain low relative to certain diatoms known for luxury Fe uptake and ferritin-based storage^77,78^. *P. calceolata*, which lacks ferritin^27^, instead prioritizes efficient utilization and Fe sparing, rather than opportunistic uptake and storage, which allows it to populate SCML environments where nitrate is available but Fe is scarce.

Motility and osmotrophic capabilities likely contribute to its success in low Fe marine environments. *P. calceolata* is motile; it may have the capability to navigate towards hotspots of organic matter rich in fixed C and N^79^. If any such chemotaxis occurs, it could also give *P. calceolata* preferential access to the labile particulate Fe pool, which typically far exceeds dissolved Fe concentrations^80,81^. Certain dynein proteins, which contribute to motility in other phytoplankton^82^, are upregulated at low Fe or light when photosynthesis is impaired (Fig. 4, Supplementary Fig. 13). These observations help explain how *P. calceolata* endures as a principal marine alga across diverse marine environments. However, resolving the details of protein function in this non-model organism, including novel transporters and a low-Fe expressed ferredoxin protein, may further reveal adaptation to Fe and light-limited marine environments. By coupling metatranscriptomic profiling with physiological measurements, this study advances our ability to link molecular regulation with carbon allocation strategies in situ. Our factorial analysis shows that co-limitation rarely produces synergy in *P. calceolata*; instead, Fe and light effects are mostly additive, with antagonistic interactions more frequent than synergistic ones (Supplementary Table 1). We interpret this as an indication of adaptation to Fe-/light-poor niches that preserves photosynthetic function and Fe economy when both constraints co-occur. Together, our findings indicate that *P. calceolata* is well adapted to Fe- and light-limited SCML conditions, and they highlight the value of integrating molecular, physiological, and biogeochemical data to connect cellular function to ecosystem processes. Determining whether these traits confer a competitive growth advantage over co-occurring taxa will require targeted interspecific comparisons under Fe/light regimes typical of SCML environments.

## Method

### Low Fe culturing

*P. calceolata* (CCMP1756) was obtained from the National Center for Marine Algae and Microbiota (NCMA). Cultures were grown in Aquil^83,84^ synthetic seawater, which was treated with Chelex 100 resin (Bio-Rad Laboratories) to remove contaminating Fe, and then microwave sterilized. Macronutrients (nitrate and phosphate) were also treated with Chelex 100, filtered through an acid-cleaned 0.2 µm PVDF filter, and added to sterile media at f/2 concentrations^85^. Vitamins stocks of B_1_ (thiamine), B_7_ (biotin), and B_12_ (cyanocobalamin) were filter sterilized and added to f/2 concentrations in the final media. Trace metal stocks without Fe were prepared according to Sunda and Huntsman (1995), and final media contained 100 µM EDTA at pH 8.1. Fe-replete cultures were amended with 100 nM FeCl_3,_ and Fe-deplete cultures contained no added Fe, but background Fe contamination in the media was measured at 0.360 ± 0.039 nM using a sulfite reduction luminol chemiluminescence flow injection method^86^. Cultures were grown at 23 °C and exposed to a 14:10 light:dark regime with either 165 or 20 µmol quanta m^-2^ s^-1^ in high light and low light treatments, respectively. Fe-replete and deplete cultures were acclimated to their conditions (Fe and light) for at least two weeks ( > 10 generations) before experimental cultures were inoculated. Average daily free Fe (Feʹ) concentrations were calculated for high light and low light treatments following Sunda et al. 2005^87^. Fe replete cultures contained 98 pM Feʹ at high light and 70 pM Feʹ at low light. Fe-deplete cultures contained 0.35 pM Feʹ at high light and 0.25 pM Feʹ at low light. Addition of 100 nM DFOB reduced calculated Feʹ at equilibrium to 5.61 fM at high light and 5.58 fM at low light, using ${K}_{{FOB},{F}{e}^{{\prime} }}^{{cond}}={10}^{11.8}$^88^. All cultures were maintained in polycarbonate bottles, which were cleaned by a 24 hour detergent (Citranox®) soak followed by a two-week treatment in 1 N trace metal grade HCl. All media preparation, manipulations, and sampling were performed in a class-100 HEPA-filtered laminar flow workbench.

Acclimated pre-cultures (Supplementary Fig. 1a) were used to inoculate 2 L of Aquil media in 2.7 L polycarbonate bottles for the diel experiment. Biological triplicate bottles were inoculated for each treatment. Fe replete pre-cultures were used for the Fe replete diel cultures, while Fe deplete cultures were used for Fe deplete, resupply and DFOB diel cultures. Diel cultures were grown under experimental conditions until the early exponential phase (~1 × 10^6^ cells mL^−1^) when the sampling program commenced.

### DNA sequencing

The genomic DNA used in the preparation of sequence libraries was derived from a pure axenic culture of *P. calceolata* CCMP1756 strain grown in natural seawater media with f/2 nutrient additions^85^. Cells were grown to the exponential growth phase and then harvested for DNA isolation. Pelleted cells were lysed in CTAB buffer (2% CTAB, 100 mM Tris-HCl pH 8.0, 20 mM EDTA, 1.4 M NaCl, with 2% PVP added immediately before use) supplemented with RNases (per 0.5 mL CTAB, 3 µL RNase A, 20 mg mL^−1^; 2 µL RNase T1, 1000 U µL^−1^). Lysates were incubated 20 min at room temperature followed by 30 min at 65 °C, extracted with an equal volume of chloroform:isoamyl alcohol, and centrifuged (10,000 × g, 20 min, RT). The aqueous phase was precipitated with an equal volume of isopropanol at -20 °C overnight, pelleted (10,000 × g, 20 min, 4 °C), washed twice with cold 70% ethanol, air-dried, and resuspended in TE buffer. The sequences used for the assembly consisted of both short and long reads and were generated from different sequencing platforms in single-end, paired-end (PE), and mate-pair (MP) modes. These included HiSeq Illumina 2 × 100 PE (insert size of 200 bp; SRR1197260) [Illumina, Inc.], HiSeq Illumina 2 × 100 MP (insert size of 3 Kbp; mate pairs submitted separately to SRA - SRR1197488 & SRR1197261), 454 GS FLX Titanium MP (insert size of 8 Kbp; SRR1197259) [Roche] and PacBio RS II [Pacific Biosciences] sequences (SRR19123260, SRR19123263, SRR19123226, SRR19123210, SRR19123240, SRR19123266).

### Genome assembly

The nuclear genome of *P. calceolata* CCMP1756 was assembled to 31.28 Mb, with a 38.98 kb mitochondrial genome (Supplementary Figs. 17, 18) and a 90.59 kb plastid genome (Supplementary Figs. 19, 20). The preliminary assembly, generated with ALLPATHS_LG (Broad Institute) using 15% each of trimmed and error-corrected Illumina PE (n = 40 × 106) and Illumina MP (*n* = 20 × 106) and 100% of 454 Flex Titanium MP (*n* = 395,212) sequences from the original yield, resulted in 231 scaffolds with an N50 of approximately 2 million bases. The estimated size from the scaffolded genome, including the missing bases, was 31.7 Mb (Data not shown). Subsequently, since the gaps in the ALLPATHS-assembled scaffolds potentially contained the missing organelle (mitochondrial and chloroplast) genomes, the longer PacBio sequences were generated and assembled with Celera WGS Assembler (http://wgs-assembler.SourceForge.net), which uses the overlap-layout-consensus method. As the pre-assembly correction can perpetuate bias from evidence reads and mis-correct polymorphic repeats, the assembly with PacBio sequences was generated without prior read correction as described in the following steps: A) preprocessing: using FastqToCA utility of Celera assembler and a k-mer size of 17 and an overlap base error rate of 0.35 (35%), chimeric and spur sequences were removed while avoiding the initial trimming of low-quality bases, B) sequence selection: using a custom script, the smallest set of the longest PacBio subreads were extracted providing ~15X genome coverage - based on the expected genome size of 30 Mb, C) assembly: the PacBio (15X coverage, *n* = 87,803) along with the 454 sequences (8 Kb mate pairs, *n* = 197,606; single reads, *n* = 386,998) were assembled with Celera assembler using runCA command with k-mer size option of 17 and an overlap error rate of 0.30. D) consensus: any base calling errors in the assembled sequences were corrected by initially mapping the trimmed Illumina 2×100 PE sequences to the scaffolds using clc_ref_assemble_long program available from the CLC Assembly Cell package (v.4.1, Qiagen) and subsequently processing the resulting alignments by the find_variations program from the same CLC package. The polished consensus sequence from the final list of 193 scaffolds yielded a genome coverage of 31.3 Mb with an N50 of 1,314,343 bases. The organellar genomes (plastid and mitochondrial) were assembled using an iterative read-mapping/contig extension approach, using short reads (SRR1197259, SRR1197260, SRR1197261, SRR1197488) for assembly, seeded by fragments matched by BLASTN searches of uncultured *Pelagomonas* plastid genome (JX297813.1) or *Aureococcus anophagefferens* mitochondrial genome (MK922345.1). Briefly, raw reads were mapped to seed contigs with HISAT2 v2.1.0 (-k 2)^89^, extracted in FASTQ format by SAMtools v1.8^90^ and BEDTools v2.26.0^91^, and then assembled by SPAdes v 3.11.1 (-k 21,33,55,77,99)^92^. The resulting contigs were used in the next rounds until scaffold completion. Organellar genomes were annotated by MFannot^93^.

### Gene models

The gene models were generated from the assembled scaffold sequences using the iterative pipeline implemented by Maker2 genome annotation program, v3.01.03^94^ as follows: (A) in the initial step, the repeat elements and the low-complexity regions within the genome sequences were masked by repeatMasker and dust/seg programs available in Maker2; (B) the preliminary gene models were deduced from mapping the transcripts generated from the *Pelagomonas* meta-transcriptome samples to *Pelagomonas* genomic sequences and by inference from homology between the translated transcripts from *Pelagomonas* meta-transcriptome and the proteins from multiple taxonomically related species obtained from Uniprot repository (manually curated Swiss-Prot and the subsets from Stramenopiles, Pelagomonadales, Sarcinochrysidales); C) the preliminary gene models and the splice-junctions characterized from mapping the transcripts and proteins from the previous step were used as seed models to train SNAP^95^ which is one of the ab initio gene predictor programs available in Maker2 package. The resulting ab initio gene models in this round were used in tandem with the mapping-based models to retrain SNAP, resulting in an improved accuracy of its prediction; D) additionally, an eukaryote-specific ab initio gene predictor Augustus^96^ was used along with the closest available training set from the alveolata species *Tetrahymena thermophila* for augmenting the gene models generated from the *Pelagomonas* genome. The ab initio models, which did not overlap with the mapping (evidence)-based Maker models, were verified against the PFAM database with InterProScan^97^ and considered valid only if they showed significant alignment. The analysis resulted in a final curated list of 12,715 gene models.

### Transcriptomics

Cells were harvested over the diel cycle for transcriptomic analysis. Cells were pelleted via centrifugation, flash frozen in liquid nitrogen, and stored at −80 °C. RNA extraction was performed using the TRIzol^TM^ Reagent (Thermo Fisher Scientific), and DNA was excluded with DNase I (TURBO DNA-free^TM^ kit, Thermo Fisher Scientific). RNA was purified using Agencourt RNAClean XP beads (Beckman Coulter), and ribosomal RNA was depleted using a Ribo-Zero rRNA Removal Kit (Illumina). cDNA libraries were prepared with a ScriptSeq v2 RNA-seq kit (Illumina) and quality evaluated on an Agilent 2200 TapeStation instrument. Sequencing of 120 cDNA libraries was performed on the Illumina MiSeq platform. Transcripts were assembled with the CLC Genomics Workbench software (Qiagen) using the RNAseq Annotation Pipeline v0.4 (RAP) as described previously^98^. Maker2 software was used to deduce gene models by mapping RAP-annotated transcripts to the *P. calceolata* genomic sequence, inference by homology to proteins from *P. calceolata* RAP assembly (translated transcripts) and proteins from multiple taxonomically related proteins, SNAP^2^ ab initio prediction^99^ and ab initio prediction by Augustus^100^ using proteins from closely related, well-annotated species. The read-mapping part of the RAP pipeline was used to assess raw read counts of modeled transcripts. Differential expression (DE) was determined through a series of comparisons using edgeR^101^ with a generalized linear model. Raw read counts were filtered to remove lowly expressed genes, normalized using the trimmed mean of M-values (TMM) method, and modeled with tagwise dispersion estimates. Pairwise contrasts were constructed to test the main effects of Fe and light, while controlling for time of day. Multiple testing correction was performed using the Benjamini-Hochberg false discovery rate (FDR) procedure, and genes with FDR ≤ 0.05 were considered significantly differentially expressed, for example, the +Fe response type is assigned to genes significantly upregulated in the Fe-replete cultures over Fe-deplete, controlling for time of day and light treatment (Supplementary Fig. 3a, Supplementary Data 15). Counts of responsive genes and the intersection of response types were visualized using the UpSetR software^102^ (Supplementary Fig. 3b,c). For the Fe/light interaction, genes were filtered to CPM > 1 in at least two samples, followed by TMM normalization. We restricted to the four factorial conditions (LL/HL × -Fe/+Fe) and excluded resupply (ReFe) and chelator (DFOB) samples. A generalized linear model was fit per gene with a factorial design including the interaction and a diel covariate (log2 counts ~ Light * Fe + TimeOfDay), with TimeOfDay ∈ {Day, Night}. Using treatment coding with LL and -Fe as baselines, the interaction was evaluated via glmFit + glmLRT on the LightHL:Fe+Fe coefficient (difference-of-differences: (HL-LL at +Fe) - (HL-LL at -Fe)). P-values were Benjamini-Hochberg adjusted. For each gene, we report: interaction log2 effect, LR statistic, P.Value, FDR, and logCPM (Supplementary Data 11). By convention, positive values indicate synergy (co-limitation exceeds additivity), negative values indicate antagonism/buffering, and values near zero indicate additivity.

Weighted gene co-expression network analysis (WGCNA) was used to identify transcriptional modules with shared diel and treatment-dependent expression dynamics^103^. Normalized log_2_ expression values were obtained from the voom transformation (limma v3.58)^104^ of read-count data generated by edgeR^101^. Genes with zero variance across samples were excluded prior to network construction. A signed adjacency matrix was computed in WGCNA (v1.73)^103^ using soft-thresholding power = 3, determined by the pickSoftThreshold() function to approximate scale-free topology (R² > 0.8). The topological overlap matrix (TOM) was calculated, and hierarchical clustering of genes based on TOM dissimilarity was performed using the blockwiseModules() function with minimum module size of 30 and a dynamic tree cut. Modules with highly correlated eigengenes (r > 0.8) were merged to yield the final set of modules, each assigned a unique color label.

Functional enrichment analysis of each module was performed using gene ontology (GO) terms derived from eggNOG-mapper (v2.1)^105^. Protein sequences from the annotated gene models were supplied to eggNOG-mapper using the eukaryotic database (taxid 2759), and GO terms were extracted from the resulting annotations. For each module, a 2×2 Fisher’s exact test was used to identify significantly overrepresented GO categories relative to the background of all annotated genes. P-values were adjusted for multiple testing using the Benjamini-Hochberg false discovery rate (FDR) procedure. Enriched GO terms and descriptions were used to annotate modules in Supplementary Data 7.

To quantify how strongly each co-expression module responded to diel cycling or iron availability, we computed correlations between module eigengenes (MEs) and experimental trait variables. For time-of-day effects, diel traits included the sine and cosine transforms of sampling time (sin24 hr, cos24 hr) as continuous circadian encodings, as well as a categorical day/night factor (day = 15 min, 3 h, 23 h; night = 11 h and 15 h). Iron treatments were encoded as four binary variables corresponding to the experimental conditions ( + Fe, -Fe, DFOB, ReFe). For each module, we calculated Pearson correlations between its eigengene and each trait. “Diel strength” was defined as the signed correlation (r) with the largest absolute value among the three diel traits; “Fe strength” was defined analogously as the signed correlation with the largest absolute value among the four iron-treatment variables. This approach captures both the direction (positive or negative relationship) and magnitude of each module’s strongest diel or iron response.

### Proteomics

Cells for proteomic analysis were pelleted by centrifugation, flash frozen in liquid nitrogen, and stored at −80 °C until extraction. Cell pellets were resuspended in methanol with a ribitol internal standard, and lysis was performed by freeze-thaw. After centrifugation, the supernatant was removed, and pellets were resuspended in SDS extraction buffer, heated to 95 °C, sonicated and debris removed, and proteins precipitated and washed with acetone. These pellets were then dissolved in 100 µl, 8 M urea, 50 mM Tris HCl, pH 8.5 at room temperature and reduced with 10 mM DTT at 54 °C in a thermomixer with 400 rpm shaking for 20 minutes. Samples were alkylated with 15 mM iodoacetamide at room temperature in the dark for 30 minutes then quenched with a second addition of DTT. Samples were made up to 200 µl with 10 mM TrisHCl, pH 8.5, and protein concentrations determined by micro BCA assay (Thermo Scientific). 120 µg aliquots of each sample were diluted with 4 M urea, 10 mM TrisHCl to give equal volumes and then further diluted with 10 mM TrisHCl to give a final concentration of 0.8 M Urea. Samples were digested with 1.2 µg trypsin at 37 °C for 5 h, followed by a second trypsin addition and incubation at 37 °C for 12 h. Following digestion, samples were acidified with 3.5 µl formic acid on ice and desalted using 50 mg Strata C18-E (55 µm, 70 Å) SPE columns (Phenomonex) to remove any primary amines and then brought to dryness in a vacuum centrifuge (Eppendorf).

Samples were labeled for Tandem Mass Tag (TMT) isobaric labeling cased quantification (Thermo Scientific, TMT10plex). Peptides were suspended in 120 µl of 50 mM HEPES, pH 8.5, to give 1 µg/µl total protein, and 70 µg aliquots of each sample were transferred to 2 mL tubes. Two internal reference standards were prepared by mixing 5 µl of each sample to give pool 1 (210 µl) or 8.3 µl of each sample to give pool 2 (350 µl). Each TMT reagent (1.6 µg per channel – two kits combined) was dissolved in 82 µl anhydrous acetonitrile. 16 µl (0.31 mg) of TMT labelling reagent was added to each sample; 48 µl and 80 µl were added to pool 1 and pool 2, respectively. Samples were vortexed briefly, centrifuged, and incubated at room temperature for 1 h. The labelling reaction was quenched with 2 µl 5% NH_4_OH. The 42 labeled samples were mixed to give five independent TMT 10-plex sets, each containing at least one internal reference channel (channel 9 or 10). Samples were then diluted with 1% formic acid to acidify and reduce the acetonitrile concentration to <5%. Each TMT set was desalted as described above. Note that TMT set E contains 70% of the desired protein amount for pool 2 (channel 10) due to insufficient volume.

Each TMT set was fractionated by high pH C18 chromatography using an Onyx C18 100 × 4.6 mm column (Phenomonex) with a 30 minute linear gradient from 5% to 30% solvent B at flow rate of 1 mL/min (solvent A, 95% water, 5% acetonitrile, 10 mM ammonium formate pH 9; solvent B, 95% acetonitrile, 5% water, 10 mM ammonium formate pH 9). 60 × 0.6 mL fractions were collected and concatenated (e.g., 1, 11, 21, 31, 41, 51) to give 10 fractions for each TMT set. Fractions were brought to dryness by vacuum centrifuge and re-suspended in 50 µl 0.5% formic acid, 3% acetonitrile.

Nanoflow LC-MS/MS was conducted using a Dionex Ultimate 3000 UHPLC (Thermo-Scientific, San Jose, CA) interfaced to the Thermo nanoflow source of an Orbitrap Velos Pro (Thermo Scientific). The ten fractions from each TMT set were analyzed in duplicate. 1 µl (1.4 ug) of each sample was injected directly onto 30 cm×0.075 mm ID, Proteo C18, 4 µm, 90 Å column, self-packed in a Picofrit fused silica nanospray emitter (New Objective, Woburn, MA). Two-hour runs were performed with a flow rate of 0.3 µL/min during sample loading and equilibration and 0.25 µL/min during sample elution. The gradient was 5% to 25% acetonitrile (0.1% formic acid) over 46 min, then 25% to 55% acetonitrile over 25 min followed by 7 min at 95% acetonitrile.

Mass spectrometry was performed in data-dependent mode using a lock mass of 445.12003 m/z for internal mass calibration. The ion spray voltage was 1.6 kV and the heated capillary temperature was 275 °C. Advanced gain control (AGC) targets were 1e6 and 5e4 for Orbitrap full MS and MSn scans, respectively, and 1e4 for ion trap MSn scans. A single microscan was performed for each MS event with maximum injection times of 250 ms for Orbitrap scans and 150 ms for ion trap MSn scans.

Orbitrap survey scans (MS1) were performed over a scan range of 300-2000 m/z at 30,000 resolution. Ion trap MS2 scans were performed for the top 10 most intense precursor ions (MS1) using collision induced dissociation (CID) with 35% normalized collision energy and a precursor isolation window of 2 m/z. MS2 scans were only collected on peptides with charge states of 2+, 3+, 4+ and 5+ with a minimum MS1 intensity threshold of 3e4 counts. Relative reporter ion abundances were determined from MS3 scans. The most intense fragment ion from each MS2 scan was selected for high-energy collision dissociation HCD with 65% normalized collision energy, and scanned in the Orbitrap at 15,000 resolution.

Proteome Discoverer version 2.2.0.338 was used to conduct database searches and to quantify reporter ions across samples. A single database search was performed for all samples (50 samples times 2 replicate injections) against our 12,715 *P. calceolata* gene models concatenated with common protein contaminants (cRAP) using SequestHT. Precursor and fragment ion mass tolerances were 15 ppm and 0.8 Da respectively. Enzyme specificity was full trypsin with two missed cleavages allowed. Fixed modifications were peptide N-terminus and Lys TMT10plex (+229.163 Da) and carbamidomethyl cysteine (+57.021 Da). Variable modifications were oxidation Met, N-Terminal Gln to pyro-Glu, N-terminal protein Met-loss, and or Acetylation. A false discovery rate (FDR) of 1% was estimated using decoy database searches and validated using Percolator with a delta Cn of 0.05^106^. Only peptides below a co-isolation threshold of 40% were used for peptide quantification. The reporter ion integration mass tolerance was 0.003 Da. Reporter abundances were reported as signal-to-noise values. To calculate protein abundance across a given TMT set, all peptides from the 10 LC fractions and two replicate injections were considered. For protein quantification, all unique peptides matching to a given protein, plus the shared peptides allocated according to Occam’s razor, were averaged.

TMT-based protein abundances were exported from Proteome Discoverer, processed, and normalized using R according to Plubell et al.^107^. Briefly, we first normalized for sample loading and labelling reaction efficiency, second to compare across the two TMT experiments, and third to scale using the weighted trimmed means as recommended by Mills et al.^108^ and described by Robinson and Oshlack^109^. Only proteins observed in all experimental TMT channels can be normalized by this procedure, and rows (proteins) containing missing values must be removed. Certain samples in our dataset gave overall lower protein abundances and contained many more missing values than other samples. Collection of these samples differed from others (filters were used instead of centrifugation), and in one case, a separate protein digest was performed after spilling some of the original digest. Including these proteins in the analysis gives a limited number of quantifiable proteins. Therefore, we removed/filtered seven low-abundance samples from the whole to yield a significantly larger list of quantified proteins. Lastly, using the un-normalized, filtered data set mentioned above, we imputed missing values with ½ of the row minimum for that protein (with the exception of the internal reference standards that were not imputed) as reported by Webb-Robertson et al.^110^. To test the effect of filtering and imputation on our proteome results, we compared protein abundance values generated in the TMT experiment with different filtering and imputation strategies prior to normalization. Alternate imputation strategies were performed using NAguideR^111^ and the best-scoring methods were selected. The methods applied were robust sequential imputation (impseqrob)^112^, sequential imputation (impseq)^113^, Glmnet Ridge Regression (GRR) https://github.com/WangLab-MSSM/DreamAI. These tests demonstrate that filtering alone, without imputation or more advanced imputation methods do not greatly affect the derived protein expression values or their relative expression across treatments (Supplementary Data 16).

Differential protein abundance was analyzed using the limma package^104^ on the normalized and imputed protein-intensity matrix. The experimental design was modeled as a three-factor linear model, allowing estimation of main effects within a unified framework. Empirical Bayes moderation was applied to improve variance estimates across proteins, and multiple testing correction was performed using the Benjamini-Hochberg false discovery rate (FDR) procedure, with proteins considered differentially abundant at FDR ≤ 0.05.

Pairwise contrasts were extracted for the major biological comparisons discussed in the text, including Fe effects within each light level and time of day, light effects under Fe-replete and Fe-deplete conditions, and day-night differences within each Fe and light treatment. The number of significantly changing proteins is summarized in volcano plots for all major contrasts (Supplementary Fig. 6-8). To test for Fe/light interaction in the proteomics data, reporter intensities were log2-transformed and quantile-normalized across samples; non-varying proteins were removed. For each protein, we fit a linear model with empirical-Bayes moderation (trend, robust): log2(intensity)∼Light×Fe+Time, with light (HL, LL), Fe (-Fe, +Fe), and time (Day, Night). The Fe × Light interaction was estimated from the LightHL:Fe+Fe coefficient using contrasts.fit and eBayes. We report, per protein, the interaction log2 fold-change, moderated t-statistic, P.Value, adj.P.Val (BH FDR), AveExpr, and B. A positive interaction indicates that the HL-LL difference is larger at +Fe than at −Fe (synergy), whereas a negative interaction indicates buffering (antagonism). Day/Night was included as a covariate; Fe-resupply and chelator perturbation samples were excluded from the interaction test.

### Photophysiology

Fv/Fm of cultures was measured over the course of the diel experiment using a WATER-ED PAM (Heinz Walz GmbH). 2 mL aliquots from all cultures were dark-adapted for 20 minutes before Fv/Fm measurements.

### Cell counts

*P. calceolata* cells were quantified using a Quanta bench-top flow cytometer (Beckman-Coulter), which was validated by comparison to manual cell counts conducted with a hemocytometer.

### Carbon and nitrogen cellular quotas

Cellular inventories of carbon and nitrogen were quantified using an ECS 4010 CHNSO Analyzer (COSTECH Analytical Technologies, Inc.). Cultures were filtered onto combusted 25 mm GF/F filters, which were then acid fumigated using concentrated HCl and then dried for 48 hours at 60 °C. Portions of each filter were packed into tin capsules and combusted at 1000 °C. CO_2_ and N_2_ gases resulting from combustion were quantified by thermal conductivity, and filter blanks were analyzed alongside samples.

### Chlorophyll measurements

Chlorophyll *a* content of cells was determined using standard fluorometric techniques^114^. Cultures were filtered onto 25 mm GF/F filters, extracted overnight in 90% acetone, and fluorescence was measured using a Turner 10-AU fluorometer before and after acidification alongside quantitative standards and blanks.

### Inductively coupled plasma mass spectrometry

Cells for elemental analysis were filtered onto acid-cleaned 47 mm polycarbonate filters using a Teflon filter rig inside a laminar flow hood. After filtration, cells were rinsed using an oxalate-EDTA solution to remove extracellular metals^115^, and filters were placed in acid-cleaned 1.5 mL polypropylene vials. Filters were digested in 20 mL quartz beakers using 2 mL 7.9 N Optima^TM^ grade nitric acid (Fisher Chemical), heated to 80 °C for 4 hours. Filters were then removed using clean Teflon forceps, placed in their original 1.5 vial, and rinsed three times with trace metal clean 18.2 MΩ·cm Milli-Q water. Rinses were collected in the quartz beakers, which were brought to dryness overnight and resuspended in 6 mL 1 N nitric acid with a 10 ppb Rh internal standard. Unused acid-cleaned filters were digested as blanks. Sample digests were analyzed on the ThermoElement XR magnetic sector ICP-MS at the Institute of Marine Sciences, University of California, Santa Cruz.

### Statistical analysis of physiological measurements

Physiological parameters (cellular C, N, Chl a, Fe, and Cu contents and derived ratios) were measured in biological triplicate cultures for each combination of light level, Fe treatment, and major timepoint (3, 15, and 23 h). All analyses were conducted in R (v4.3)^116^114 using the tidyverse^117^115, multcompView^118^, and stats packages. For each parameter, pairwise Welch’s t-tests were performed among Fe treatments within the same light level and timepoint to evaluate treatment effects while accounting for unequal variances among groups. Resulting P-values were adjusted for multiple comparisons using the Holm method, and compact letter displays were generated to denote groups that were not significantly different from each other (α = 0.05). To evaluate the effect of light level, additional pairwise Welch’s t-tests were performed between high-light (HL) and low-light (LL) treatments at each Fe condition and time point. Mean values and standard deviations were calculated from biological replicates, and data were visualized using ggplot2^119^.

For each endpoint, we tested whether the double-limited condition (LL & −Fe) exceeded the additive expectation from single limitations by estimating the Fe × Light interaction in a factorial model. For each physiological trait and time point (3, 15, 23 h), we analyzed only the Fe-replete (+Fe) and Fe-limited (−Fe) treatments under high light (HL) and low light (LL). We fit a two-way linear model with interaction for each trait/timepoint independently: Value ~ Light × Fe, with LL and -Fe as reference levels. The Fe × Light interaction term (“LightHL:Fe+Fe”) quantifies the difference-of-differences: (HL-LL at +Fe)-(HL-LL at -Fe). We report the interaction estimate (with standard error, t-statistic, and p-value), interpreting positive values as synergy (the HL-LL difference is larger at +Fe) and negative values as buffering/antagonism (the HL-LL difference is smaller at +Fe). P-values were FDR-adjusted within each time point (Benjamini-Hochberg). For context, we also provide the four cell means (HL+Fe, LL+Fe, HL-Fe, LL-Fe) and the signed difference-of-differences (Supplementary Data 10).

### CCE abundance, carbon estimates, and gene expression

*P. calceolata* 18S rRNA gene abundances for both the V4 and V9 region, as well as transcript abundances, were obtained from a subset of DNA and RNA samples from the NCOG dataset, which encompasses wide-ranging variability in the California Current Ecosystem^66,67^. To estimate absolute abundances (copies L^−1^), genomic DNA from *Schizosaccharomyces pombe* was used as an internal standard during DNA extraction^120^. The number of *Pelagomonas* reads was divided by the ratio of *S. pombe* reads to the number of *S. pombe* 18S copies added (1.29 × 10^7^ to 2.79 × 10^7^ depending on the sample). The total number of copies was then normalized to the volume filtered. To assess the impact of uncertainty in cellular carbon content and genome copy number on estimated biomass ratios, we calculated *P. calceolata*:cyanobacteria particulate carbon ratios under a range of plausible physiological assumptions. Cell abundances of *P. calceolata* were inferred from 18S rDNA V9 copy numbers quantified from DNA samples. Conversion of *P. calceolata* 18S copies to cell numbers assumed two 18S rDNA copies per genome^27^ and genome ploidy levels ranging from 1 to 8 (i.e., 2–16 total 18S copies per cell). Cellular carbon content (quota) was varied across empirically reasonable ranges derived from culture measurements: *P. calceolata* = 1200–1800 fg C cell^−1^; *Prochlorococcus* = 32–60.9 fg C cell^−1^ ^121,122^; *Synechococcus* = 92.4-132 fg C cell^−1^ ^121,123^. For each scenario, *P. calceolata* and cyanobacterial carbon per liter were calculated by multiplying cell abundance by the respective quota, and their ratio was computed for both surface and subsurface chlorophyll maximum layer (SCML) samples. Violin plots were used to visualize the distribution of *P. calceolata*:cyanobacteria carbon ratios under each set of assumptions. In all scenarios, *P. calceolata* accounted for more carbon per liter than *Prochlorococcus* and/or Synechococcus in a significant number of samples (Supplementary Fig. 16). Main text figures used the mean experimentally measured *P. calceolata* carbon quota of 1251 fg C cell^-1^, and assumed 2 18S copies per genome and one genome per cell (Fig. 5)*. Prochlorococcus* and *Synechococcus* abundances were determined by flow cytometry using previously reported methods^124^, and converted to C using estimates of 32 and 101 fg C cell^-1^, respectively^121,123^, which have been used to estimate cyanobacterial C in this region^125^.

To examine *Pelagomonas* gene expression, poly-A selected metatranscriptomes were taxonomically annotated with PhyloDB v1.076^67,98^, using the lineage probability index (LPI) framework of Podell & Gaasterland (2007^126^). PhyloDB includes 11 additional pelagophyte references, and the assembled ORFs annotated as *P. calceolata* had a high mean LPI (96%), supporting confident species-level assignment. Predicted proteins from ORFs classified as *P. calceolata* were aligned to the *P. calceolata* genome with DIAMOND BLASTP (E-value ≤ 1e-3), and only reads mapping to these annotated ORFs were counted toward *P. calceolata* expression. Read mapping used Qiagen CLC Assembly Cell v5.2.1.216695-202010091408 (paired-end; default settings). Under these defaults, ties (equal-score multi-hits) are randomly assigned to a single locus and therefore are not double-counted. Across all reads, the average multi-mapping rate was 9.9%, representing a small fraction of the total; these include both within-genome multi-hits and occasional cross-taxon ties, but the latter are minimized by the ORF-level taxonomic filter. Gene abundances were then summed and normalized with DESeq2 v1.42.1^127^. Consistent with these assignments, 18S rRNA surveys (V4 and V9) in the same samples indicated that *P. calceolata* comprised ~64% and ~71% of Pelagophyceae reads, respectively, further reducing the probability that non-*P. calceolata* transcripts were misattributed.

### Iron uptake rate measurements

Uptake of Fe complexed to the model hydroxamate siderophore desferrioxamine b (DFOB) was measured using radiolabeled ^59^Fe uptake assays, as described previously^47^. *P. calceolata* cells were grown in Aquil media at limiting and replete Fe concentrations (same concentrations of added Fe as the diel experiment). *Phaeodactylum tricornutum* cultures (included as a positive control known to express an ortholog of the *P. calceolata* siderophore binding protein^47^) were grown at ~15 pm Feʹ, 7.5 nM total Fe in Aquil media. Cell concentrations were limited to less than 5 × 10^5^ cells mL^-1^ (5 × 10^4^ cells mL^-1^ for *P*. tricornutum) to avoid changes in physiology and media pH. ^59^Fe was incubated overnight with DFOB at a 4:5 molar ratio (Fe:DFOB) in high purity water at pH 3, then adjusted to pH 8 with trace metal-grade NaOH. Precomplexed ^59^Fe-containing FOB was added to standard Fe-free Aquil media at 10x final assay concentration (2.5 nM FOB) and again equilibrated overnight. At the beginning of the experiment, ^59^Fe-DFOB media was added to cultures to obtain the final assay Fe-DFOB concentration of 250 pM. Uptake assays were shielded from actinic light, filtered onto 2.0 μm PTFE filters, washed with an oxalate EDTA solution, and preserved in Ecolite (MP Biomedicals). Uptake rates were determined from time-series measurements of intracellular iron content (amol Fe cell^-1^) for each culture treatment. Data were analyzed in R (v4.3)^116^ using the tidyverse^117^, lme4, lmerTest^128^, and emmeans^129^ packages. For each culture, intracellular Fe was regressed against incubation time (min) to estimate the rate of Fe accumulation (slope = uptake rate). A linear mixed-effects model was used to account for replicate structure. Culture-specific slopes (amol Fe cell^-1^ min^-1^) and 95 % confidence intervals were obtained with emmeans::emtrends(), and pairwise comparisons were performed with Tukey adjustment. Rates were visualized as bar plots with confidence intervals and compact letter displays indicating statistically distinct groups (α = 0.05). Figures were generated with ggplot2^119^.

### Reporting summary

Further information on research design is available in the Nature Portfolio Reporting Summary linked to this article.

## Supplementary information

Supplementary Information

Descriptions of Additional Supplementary Files

Supplementary Data 1

Supplementary Data 2

Supplementary Data 3

Supplementary Data 4

Supplementary Data 5

Supplementary Data 6

Supplementary Data 7

Supplementary Data 8

Supplementary Data 9

Supplementary Data 10

Supplementary Data 11

Supplementary Data 12

Supplementary Data 13

Supplementary Data 14

Supplementary Data 15

Supplementary Data 16

Reporting Summary

Transparent Peer Review file

## Source data

Source Data

## Data Availability

Genomic data generated in this study have been deposited with the NCBI accession JBNOXR000000000. Raw sequencing reads used to generate the genome assembly have been deposited under the following accessions: SRR1197260; SRR1197488; SRR1197261; SRR1197259; SRR19123260; SRR19123263; SRR19123226; SRR19123210; SRR19123240 & SRR19123266. Pre-existing sequencing data were used to assemble the plastid and mitochondrial genomes: JX297813.1 (plastid) & MK922345.1 (mitochondrial). Transcriptomic data are included in BioProject PRJNA193556. The mass spectrometry proteomics data have been deposited to the ProteomeXchange Consortium via the PRIDE partner repository with the dataset identifier PXD065159 and 10.6019/PXD065159. The NCOG^66,67^ DNA and RNA sequence data analyzed in this study have been deposited in the National Center for Biotechnology (NCBI) sequence read archive under the BioProject accession numbers PRJNA555783, PRJNA665326, and PRJNA804265. ICP-MS data are available in Supplementary Data 1. All other data used in the study are deposited in the Zenodo repository 10.5281/zenodo.18718641 [https://zenodo.org/records/18718641]^130^, including Supplementary Data 1–16. Source data are provided with this paper.

## Code availability

Custom R scripts used for RNA-seq and proteomic statistical analyses, including edgeR and limma workflows, are available at the Zenodo repository under 10.5281/zenodo.17859961 [https://zenodo.org/records/18718641].
