# Supplementary material for: Molecular and physiological acclimation to low light and iron scarcity in a globally abundant oceanic pelagophyte
Source: Nat Commun. 2026 Apr 20;17:5480. doi: 10.1038/s41467-026-71628-0 (PMC13284251; doi:10.1038/s41467-026-71628-0)
Supplement: Supplementary file 1 — Supplementary Information [file 41467_2026_71628_MOESM1_ESM.pdf]

Supplementary material for:

Molecular and physiological acclimation to low light and iron scarcity in a globally abundant oceanic pelagophyte

Coale, T. H.[1], Lampe, R. H.[2,3], Tan, M[2]., Rowland, E.[4], Füssy, Z.[2,3,5], Venepally, P.[2], Zheng, H.[2], McCrow, J.[2], Bertrand, E. M.[4] & Allen, A.E.[2,3]

[1]Ocean Sciences Department, University of California, Santa Cruz, Santa Cruz, CA 95064

[2]Microbial and Environmental Genomics, J. Craig Venter Institute, 4120 Capricorn Lane, La Jolla, CA, 92037, USA
[3]Integrative Oceanography Division, Scripps Institution of Oceanography, University of California San Diego, 9500 Gilman Drive, La Jolla, CA, 92093 USA
[4]Department of Biology, Dalhousie University, 1355 Oxford St, Halifax, NS, Canada B3H 4R2
[5]Faculty of Science, Department of Chemistry, University of South Bohemia, 370 05 České Budějovice, CZ

This file includes:

Supplementary Figures 1-20
Supplementary Table 1

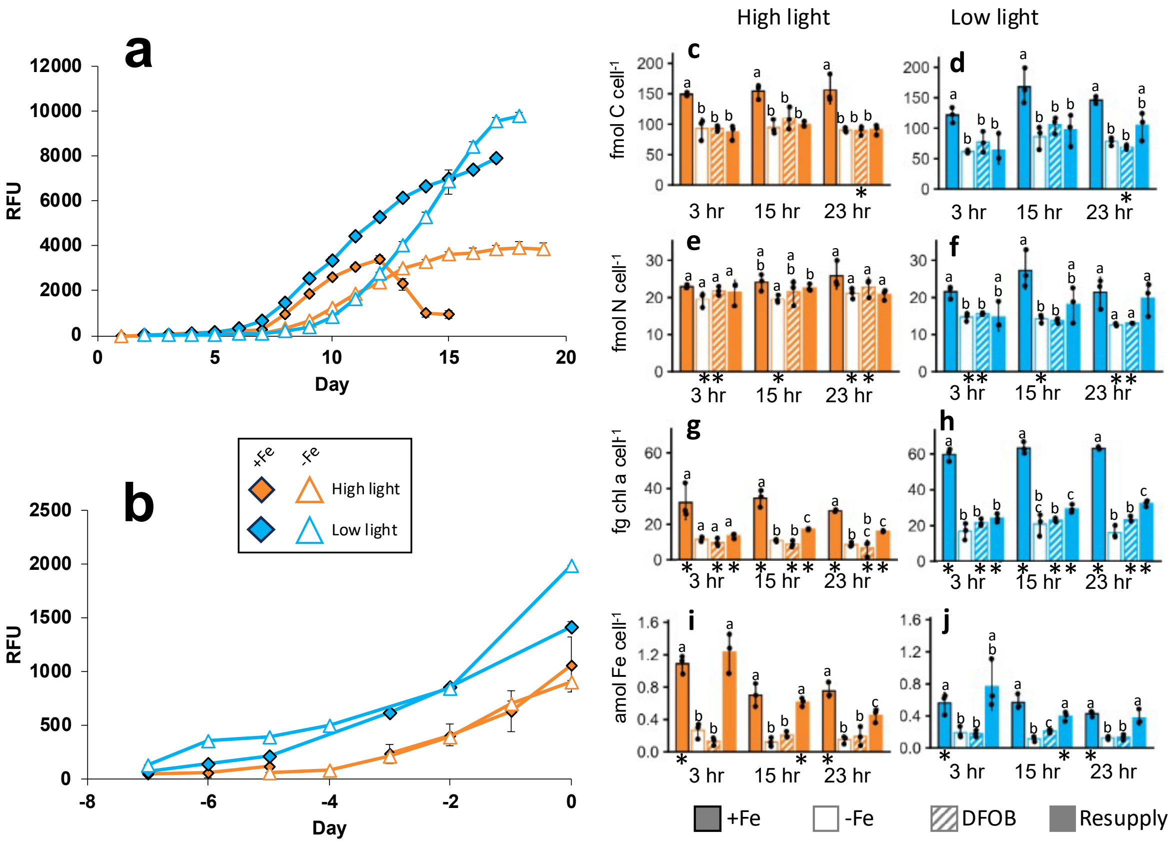

Supplemental Fig.1 Acclimation and cellular contents in Fe/light limited *P. calceolata*. **a**, Growth in pre-experiment cultures used to determine exponential growth phase by fluorescence (relative fluorescence units, RFU). Points represent mean RFU of triplicate cultures with error bars showing ± 1 SD. **b**, Growth of cells in experimental bottles prior to initiation of the experiment at Day 0. Points represent mean RFU of triplicate cultures with error bars showing ± 1 SD. **c-j,** Cellular quotas at the three major time points for carbon (**c,d**)**,** nitrogen (**e, f**) , chlorophyll *a* (**g, h**) , and Fe (**i, j**) . High light treatments are in orange, low light in blue. Bars show mean of biological triplicate (n = 3 per treatment) cultures with error bars representing ± 1 SD. Letters above bars denote groups that are not significantly different from each other, based on pairwise comparisons within the same timepoint and light level (two-tailed Holm-adjusted $P < 0.05$), Asterisks (*) indicates significant differences between the light levels at the same timepoint and Fe treatment (two-tailed Welch's t-test, $P<0.05$). Individual biological replicates (n=3) are plotted as black dots. Source data are provided as a Source Data file.

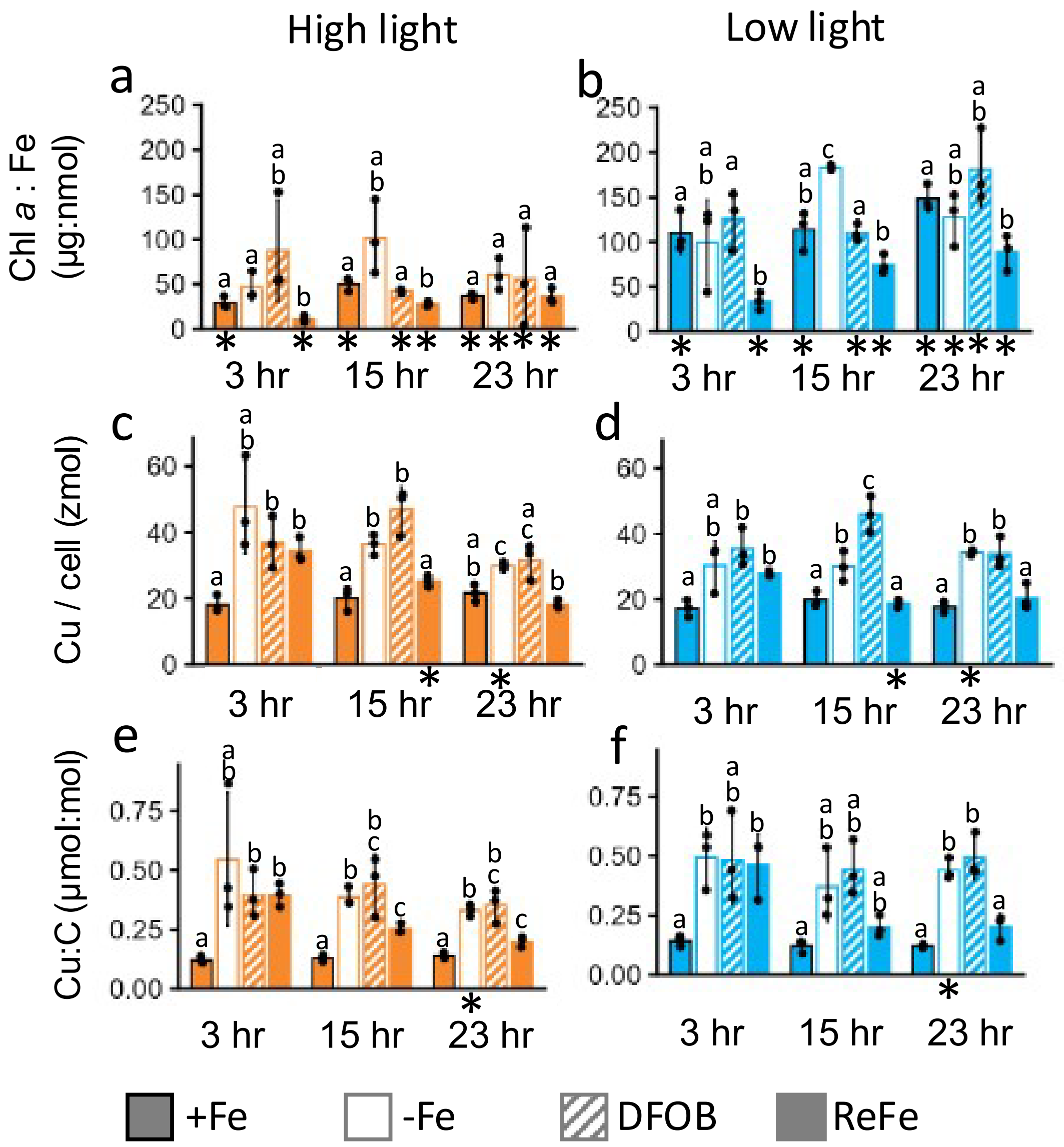

Supplemental Fig.2 Trace metal utilization in Fe/light limited *P. calceolata*. Ratios of chlorophyll *a* to iron (**a,b**), cellular copper quotas (**c,d**), and ratios of copper to carbon (**e,f**) at the three major time points. High light treatments are in orange, low light in blue. Bars show mean of biological triplicate (n = 3 per treatment) cultures with error bars representing ± 1 SD. Letters above bars denote groups that are not significantly different from each other, based on pairwise comparisons within the same timepoint and light level (2-tailed Holm-adjusted $P < 0.05$), Asterisks (*) indicates significant differences between the light levels at the same timepoint and Fe treatment (2-tailed Welch's t-test, $P<0.05$). Individual biological replicates (n=3) are plotted as black dots. Source data are provided as a Source Data file.

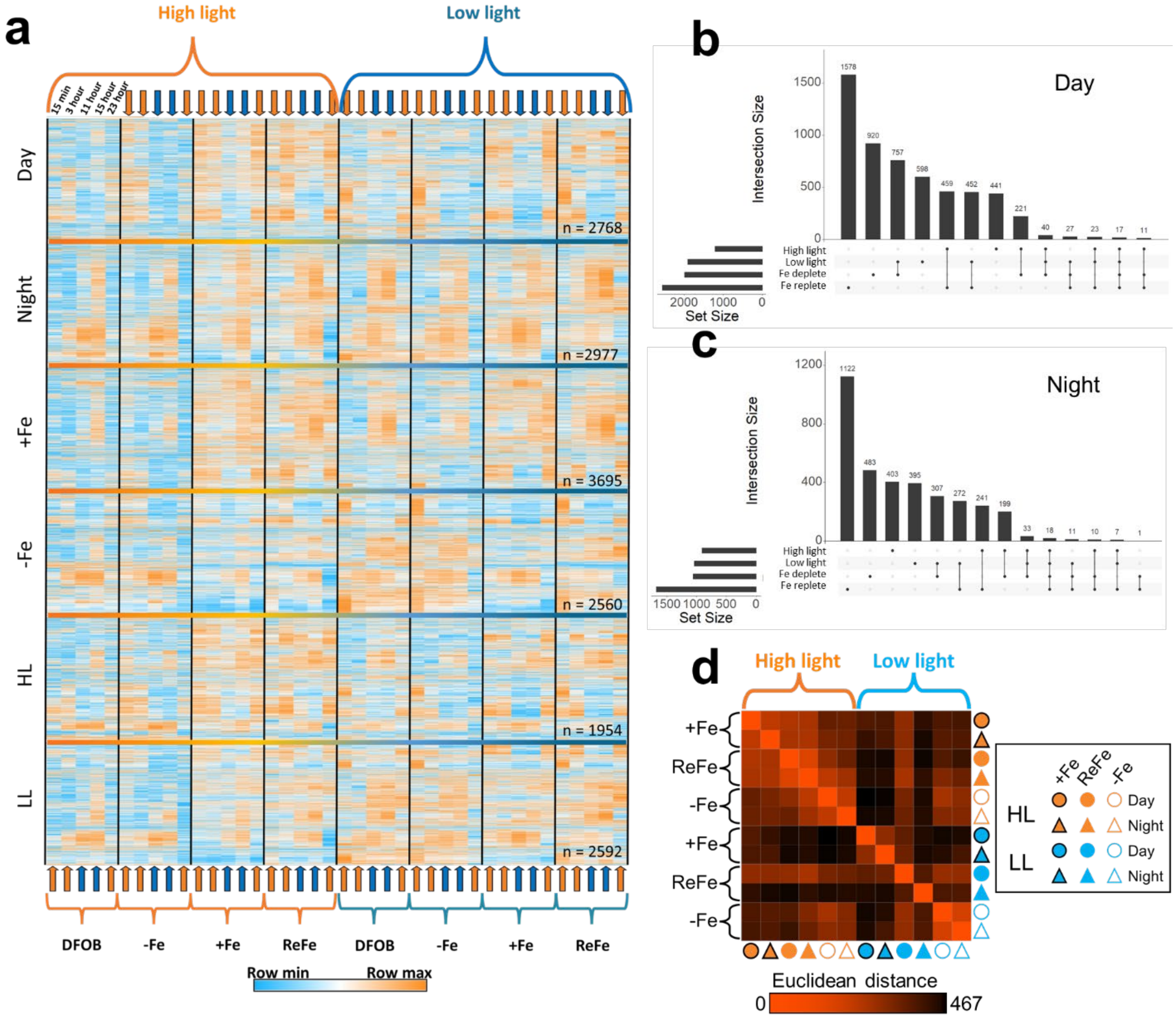

Supplemental Fig.3 Whole transcriptome patterns related to Fe, light and time of day. **a,** Heatmap showing average CPM transcript abundance (row-normalized) of differentially expressed genes across the diel cycle, grouped by EdgeR response type. Orange brackets indicate high light treatments and blue brackets show low light. Orange arrows show day time points and blue arrows show night time points. Numbers indicate total number of genes in each response type. **b,** UpSet plot showing intersections of Fe and light response types from day samples only. **c,** same as b but for night samples. **d**, Heatmap showing Euclidean distances between all protein samples. Mean normalized protein quantities for each set of triplicate samples were used to calculate distances. Source data are provided in Supplementary Data 2 and 15.

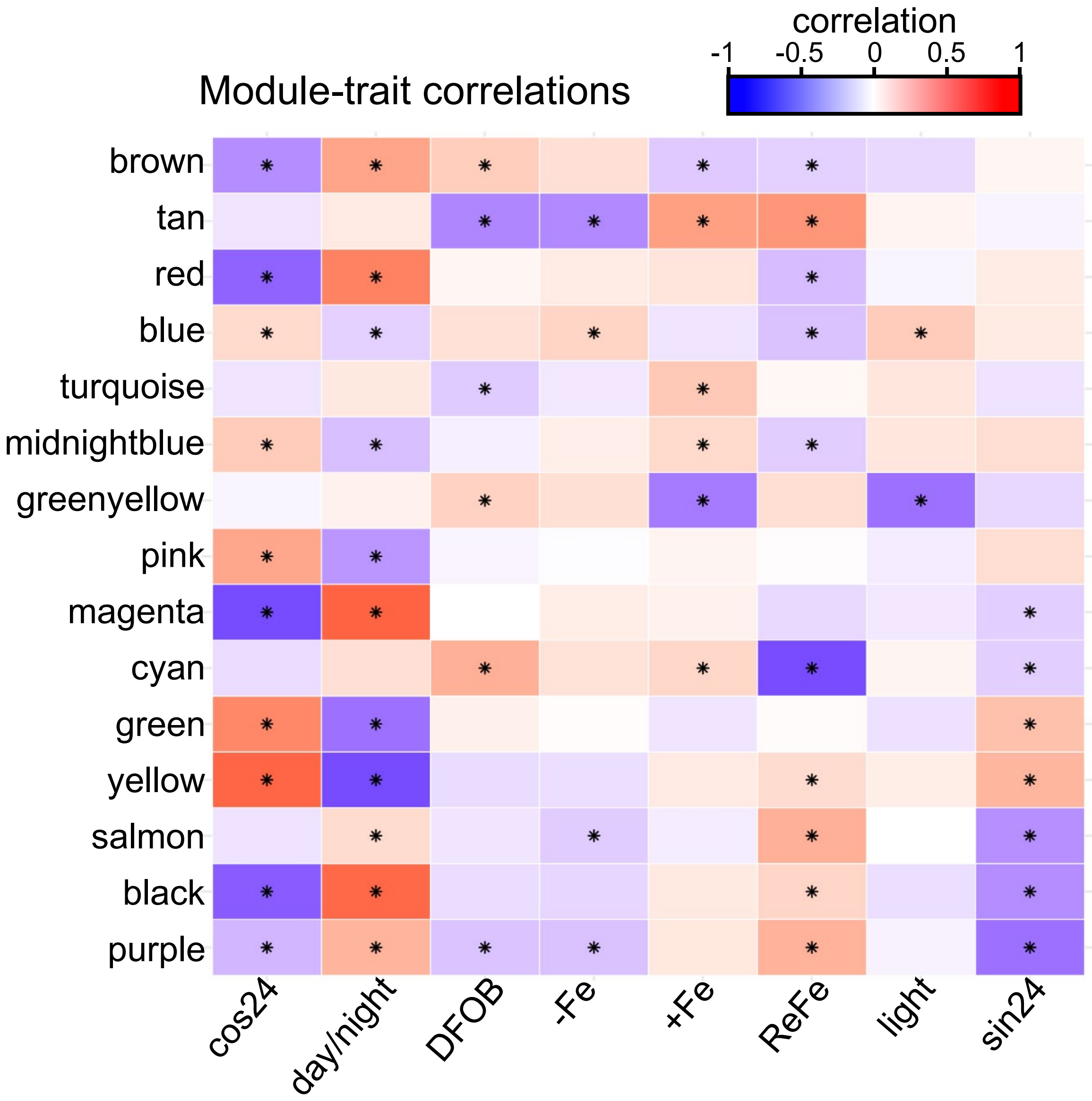

Supplemental Fig.4. Module-trait correlation heatmap from WGCNA analysis. Heatmap showing Pearson correlations between module eigengenes (rows) and experimental traits (columns), including diel variables (sin24, cos24, and day/dight classification) and iron/light treatments (+Fe, -Fe, ReFe, and DFOB). Each cell represents the strength and direction of correlation (red = positive, blue = negative), and black asterisks indicate statistically significant associations ($P < 0.05$). Module eigengenes summarize the first principal component of gene expression within each co-expression module and represent the dominant expression pattern for that gene cluster. Modules showing strong correlations with diel traits exhibit periodic expression consistent with diurnal regulation, while those correlated with Fe or light treatments respond to their availability regardless of direction. Source data are provided as a Source Data file.

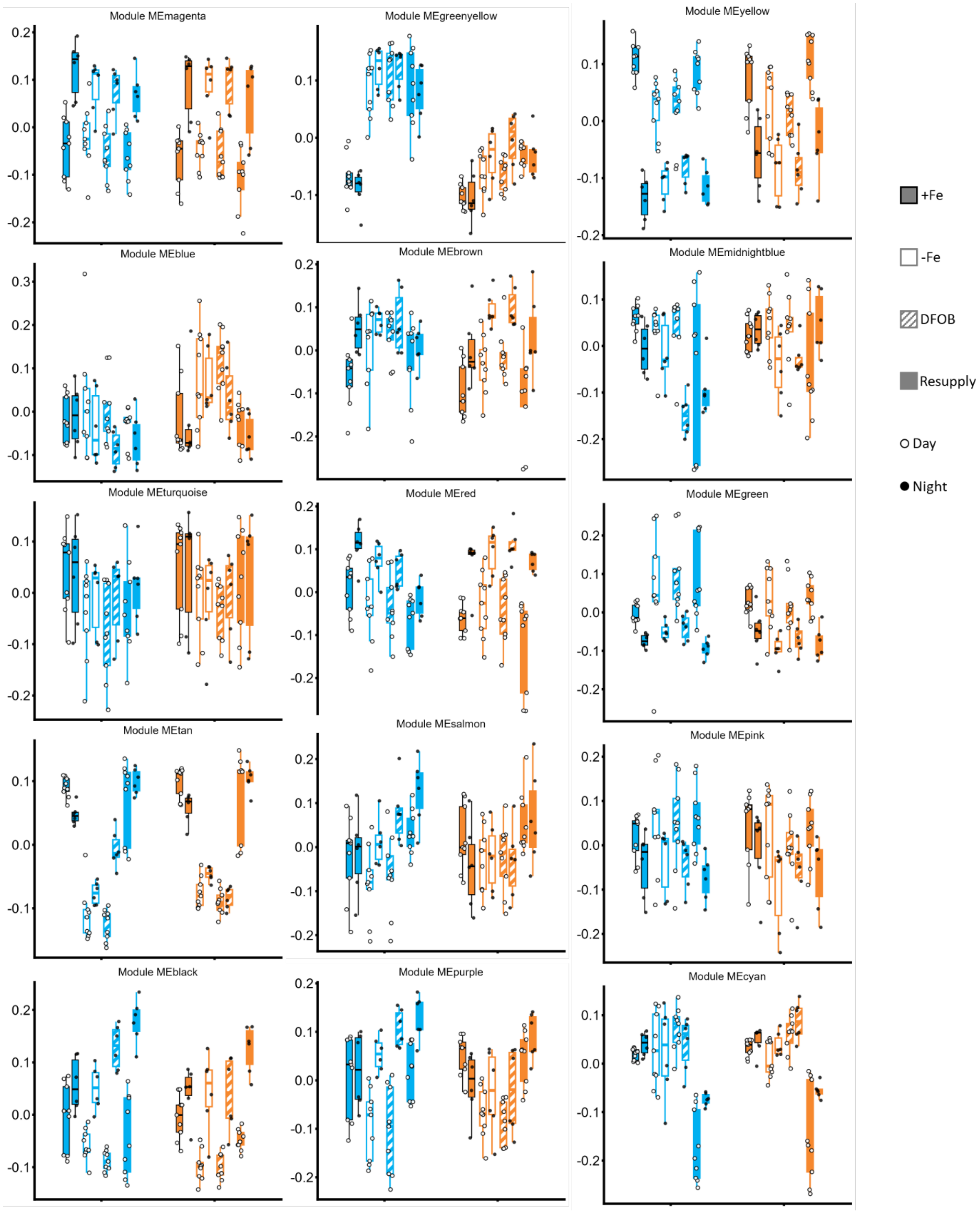

Supplemental Fig 5. WGCNA module eigengenes across iron, light, and diel conditions. Expression patterns of WGCNA modules across light, Fe, and diel conditions. Boxplots show module eigengene values (first principal component of module expression)

for each WGCNA module across experimental treatments. For each light level (LL, HL), module eigengenes are shown for all four Fe treatments (+Fe, -Fe, DFOB, ReFe) with separate boxes for Day (0, 3, 23 h; white points) and Night (11, 15 h; black points). Box colors and patterns indicate Fe treatment identity: +Fe (solid fill), -Fe (outline only), DFOB (diagonal color stripes), and ReFe (solid fill with colored border). Patterns and fill colors reflect the light level (orange for HL, blue for LL). Points represent biological replicates. Source data are provided as a Source Data file.

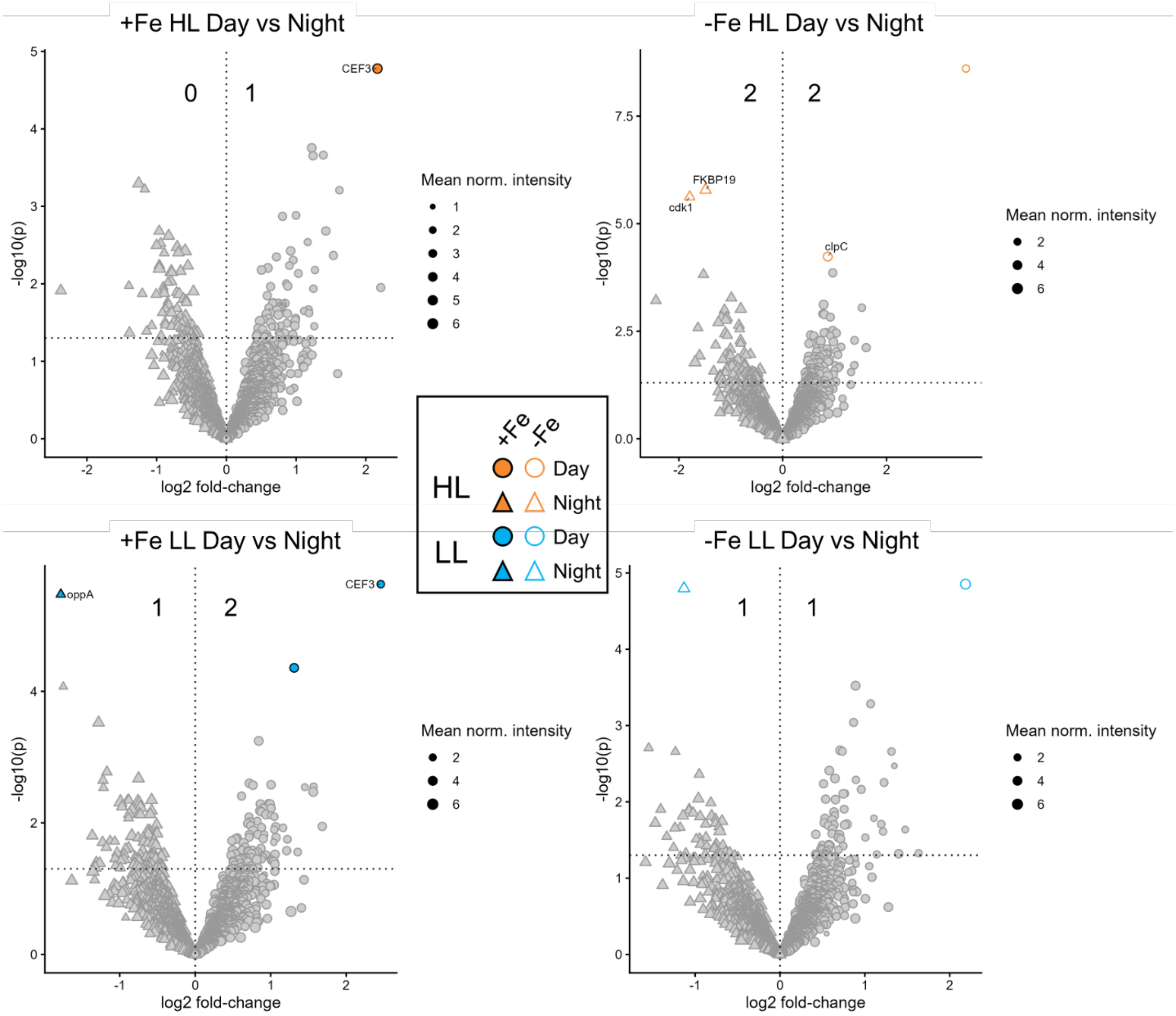

Supplemental Fig 6. Differential protein abundance in *P. calceolata* in day versus night samples. Volcano plots show results from limma-based differential expression analyses of normalized and imputed protein intensities. Each point represents a quantified protein; the x-axis shows $\log_2$ fold-change between conditions, and the y-axis shows –$\log_{10}$(raw p value). Proteins significant at FDR < 0.05 are highlighted according to experimental factors and up-regulated side. Count of significantly expressed proteins are indicated in each plot. Marker size is scaled by mean normalized protein intensity. oppA, periplasmic oligopeptide-binding protein; CEF3, elongation factor 3; cdk1, cyclin-dependent kinase 1; FKBP19, FKBP-type peptidyl-prolyl cis-trans isomerase; clpC, ATP-dependent Clp protease ATP-binding subunit. Source data are provided as a Source Data file.

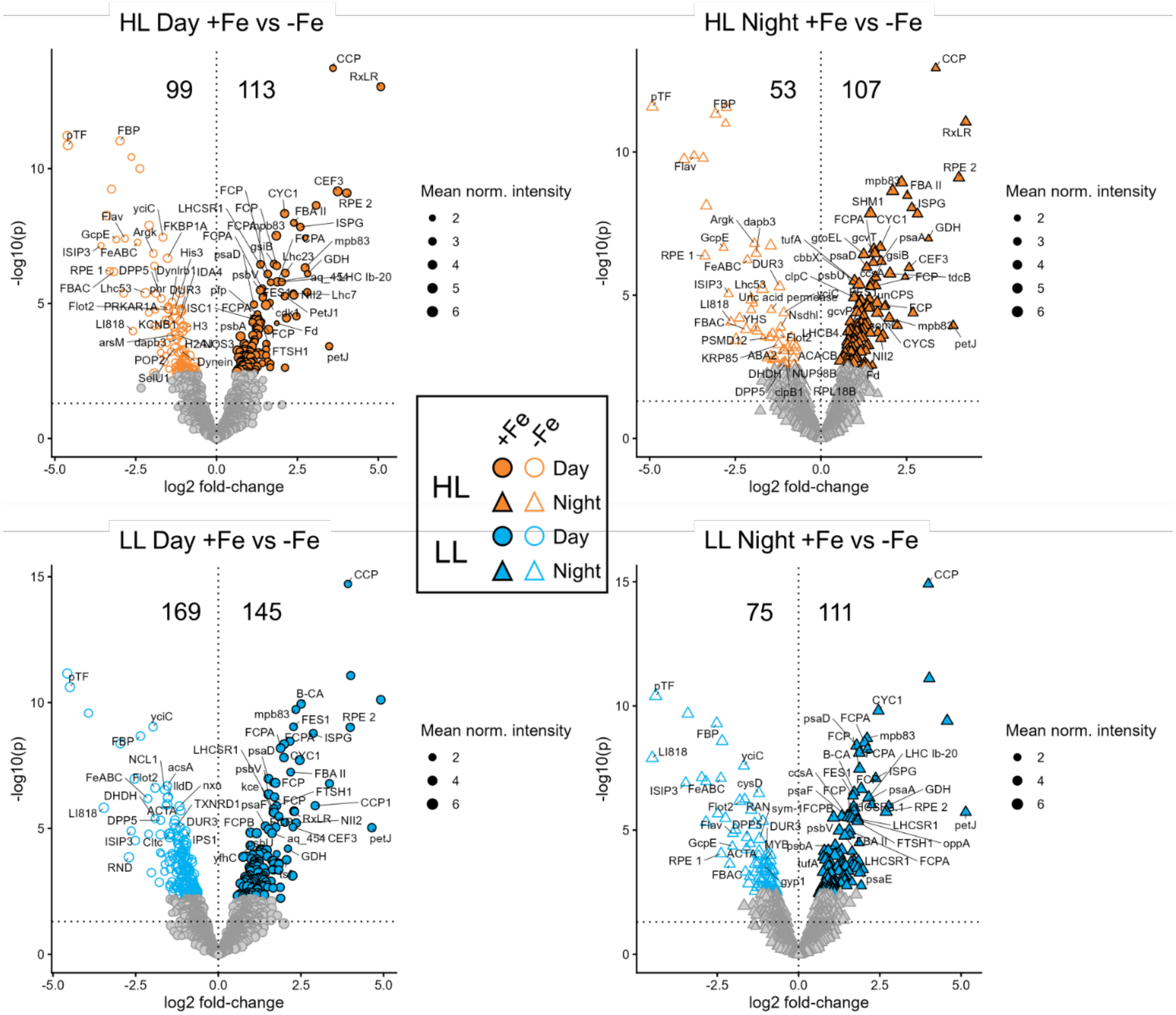

Supplemental Fig 7. Differential protein abundance in *P. calceolata* under Fe treatments. Volcano plots show results from limma-based differential expression analyses of normalized and imputed protein intensities. Each point represents a quantified protein; the x-axis shows $\log_2$ fold-change between conditions, and the y-axis shows –$\log_{10}$(raw p value). Proteins significant at FDR < 0.05 are highlighted according to experimental factors and up-regulated side. Count of significantly expressed proteins are indicated in each plot. Marker size is scaled by mean normalized protein intensity. AMT = ammonia transporter, β-CA = beta carbonic anhydrase, CPS1 = carbamoyl-phosphate synthetase, Cytb = cytochrome b, CytC1 = cytochrome C1, DUR = urea transporter, DYN = dynein, FBA = fructose-bisphosphate aldolase, FBP = fructose 1,6-bisphosphatase, Fd = erredoxin, Fd-NIR = ferredoxin nitrite reductase, FeABC = ferrichrome ABC transporter protein, Fe-L = Fe bound to organic ligand, Flav = flavodoxin, FNT = formate/nitrite transporter, FRE = ferric reductase, GOGAT = gluatmate synthase, GS = glutamine synthetase, ISIP3 = iron starvation induced protein 3, Lhc = light harvesting complex protein, MCO = multicopper oxidase, NAT = nucleobase-ascorbate transporter, NCS2 = uracil-xanthine permease, NIR = NADPH nitrite reductase, NRT1 = nitrate transporter, PetJ

= cytochrome C6, psaA = photosystem I reaction center protein A, psbA = photosystem II reaction center protein A, pTF = phytotrasferrin, rbcL = ribulose 1,5 bisphosphate carboxylase large subunit, rbcS = ribulose 1,5 bisphosphate carboxylase. Other protein annotations are found in Supplementary Data 3. Source data are provided as a Source Data file.

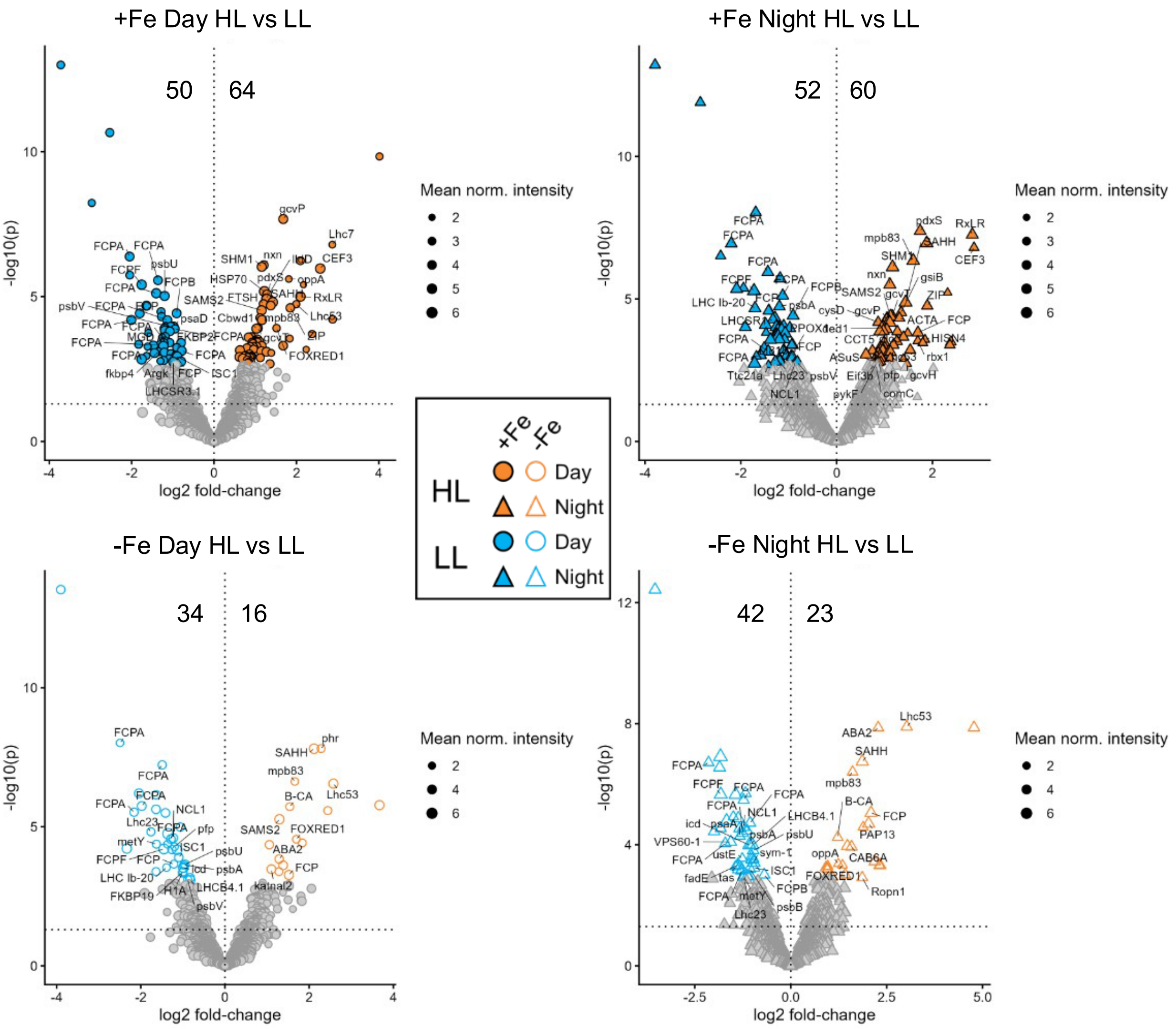

Supplemental Fig 8. Differential protein abundance in *P. calceolata* under light treatments. Volcano plots show results from limma-based differential expression analyses of normalized and imputed protein intensities. Each point represents a quantified protein; the x-axis shows $\log_2$ fold-change between conditions, and the y-axis shows –$\log_{10}$(raw p value). Proteins significant at FDR < 0.05 are highlighted according to experimental factors and up-regulated side. Count of significantly expressed proteins are indicated in each plot. Marker size is scaled by mean normalized protein intensity. AMT = ammonia transporter, β-CA = beta carbonic anhydrase, CPS1 = carbamoyl-phosphate synthetase, Cytb = cytochrome b, CytC1 = cytochrome C1, DUR = urea transporter, DYN = dynein, FBA = fructose-bisphosphate aldolase, FBP = fructose 1,6-bisphosphatase, Fd = erredoxin, Fd-NIR = ferredoxin nitrite reductase, FeABC = ferrichrome ABC transporter protein, Fe-L = Fe bound to organic ligand, Flav = flavodoxin, FNT = formate/nitrite transporter, FRE = ferric reductase, GOGAT = gluatmate synthase, GS = glutamine synthetase, ISIP3 = iron starvation induced protein 3, Lhc = light harvesting complex

protein, MCO = multicopper oxidase, NAT = nucleobase-ascorbate transporter, NCS2 = uracil-xanthine permease, NIR = NADPH nitrite reductase, NRT1 = nitrate transporter, PetJ = cytochrome C6, psaA = photosystem I reaction center protein A, psbA = photosystem II reaction center protein A, pTF = phytotrasferrin, rbcL = ribulose 1,5 bisphosphate carboxylase large subunit, rbcS = ribulose 1,5 bisphosphate carboxylase. Other protein annotations are found in Supplementary Data 3. Source data are provided as a Source Data file.

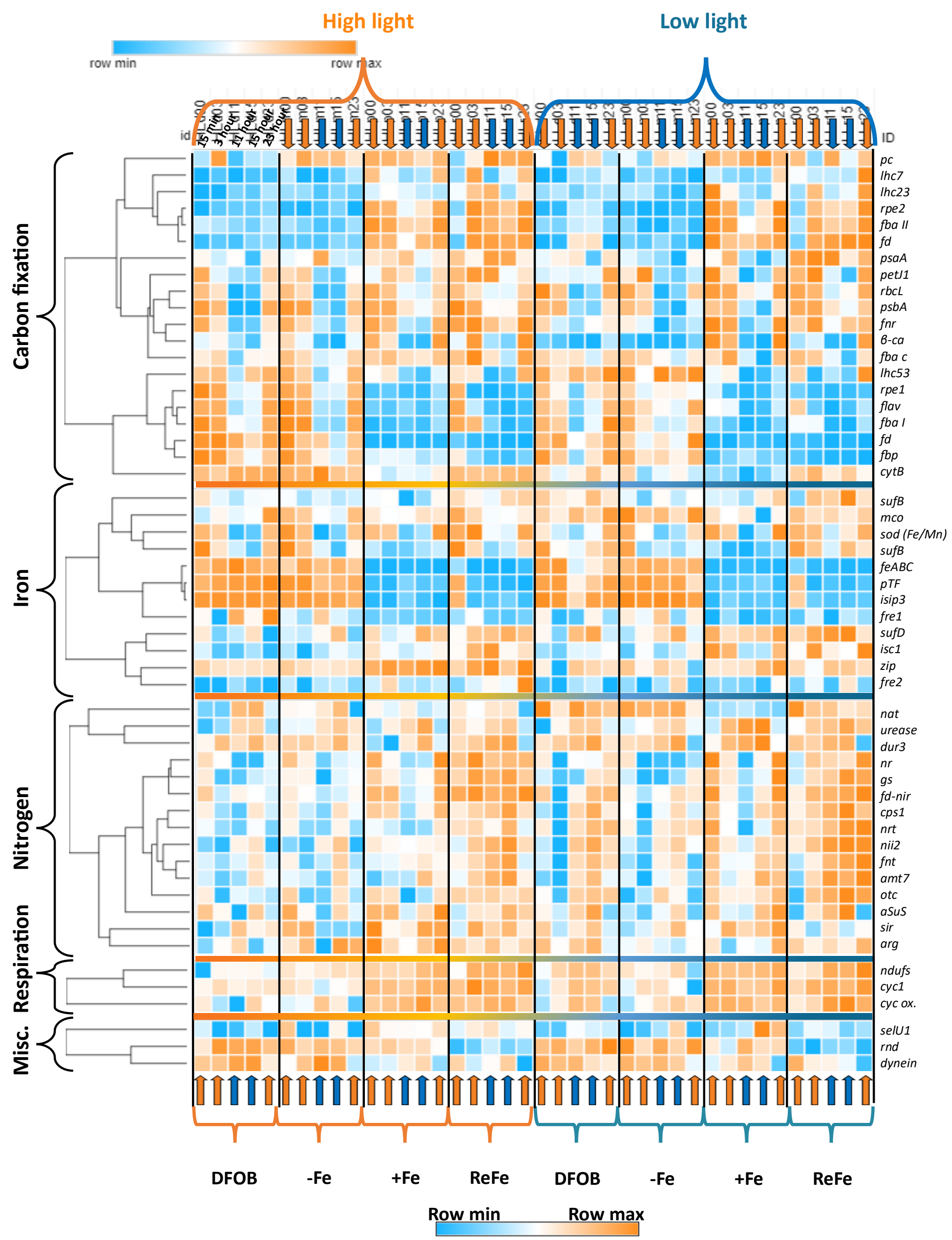

Supplemental Fig.9 Transcriptomic response of C, N and Fe metabolism. Heatmap showing average CPM transcript abundance of selected genes across the diel cycle. Orange brackets indicate high light treatments and blue brackets show low light. Orange arrows show day time points and blue arrows show night time points. *amt7*, ammonia transporter; *arg*, arginase; *aSuS*, argininosuccinate synthase; *β-ca*, carbonic anhydrase; *cyc ox.*, cytochrome c oxidase; *cyc1*, cytochrome c; *cytB*, cytochrome b6; *dur3*, urea-proton symporter; *fba I*, fructose bisphosphate aldolase class I; *fba II*, fructose bisphosphate aldolase class II; *fbac*, fructose bisphosphate aldolase class I, cytosolic; *fbp*, fructose bisphosphatase; *fd*, ferredoxin; *fd-nir*, nitrite reductase - ferredoxin; *sod*

(Fe/Mn), Fe/Mn superoxide dismutase; *feABC*, ferrichrome ABC transporter-like protein; *flav*, flavodoxin; *fnr*, ferredoxin NADP+ reductase; *fnt*, formate nitrite transporter; *fre*, ferric reductase ; *gcpE*, (E)-4-hydroxy-3-methylbut-2-enyl-diphosphate synthase; *gs*, glutamate synthetase; *isc1*, Fe-S assembly protein; *isip3*, iron starvation induced protein 3; *lhc23*, putative plastid light harvesting protein isoform 23; *lhc53*, putative plastid light harvesting protein isoform 53; *lhc7*, putative plastid light harvesting protein isoform 7; *mco*, multicopper oxidase ; *ndufs*, NADH:ubiquinone oxidoreductase; *ngit*, Neurotransmitter-gated ion-channel; *nii2*, nitrite reductase - NADH; *nr*, nitrate reductase; *nrt*, nitrate/nitrite transporter; *otc*, ornithine carbamoyltransferase ; *pc*, plastocyanin; *petJ1*, cytochrome c6; *psaA*, photosystem I core protein PsaA; *psbA*, photosystem II P680 reaction center D1 protein ; *pTF*, phytotransferrin; *rbcL*, ribulose-1,5-bisphosphate carboxylase/oxygenase large subunit; *rnd*, resistance-nodulation-division transporter; *rpe*, ribulose 5-phosphate 3-epimerase; *selU1*, selenoprotein; *sir*, nitrite/sulfite reductase - ferredoxin; *suf*, Fe-S assembly protein; *zip*, zinc responsive transporter/iron responsive transporter-like protein. Source data are provided in Supplementary Data 2 and 3.

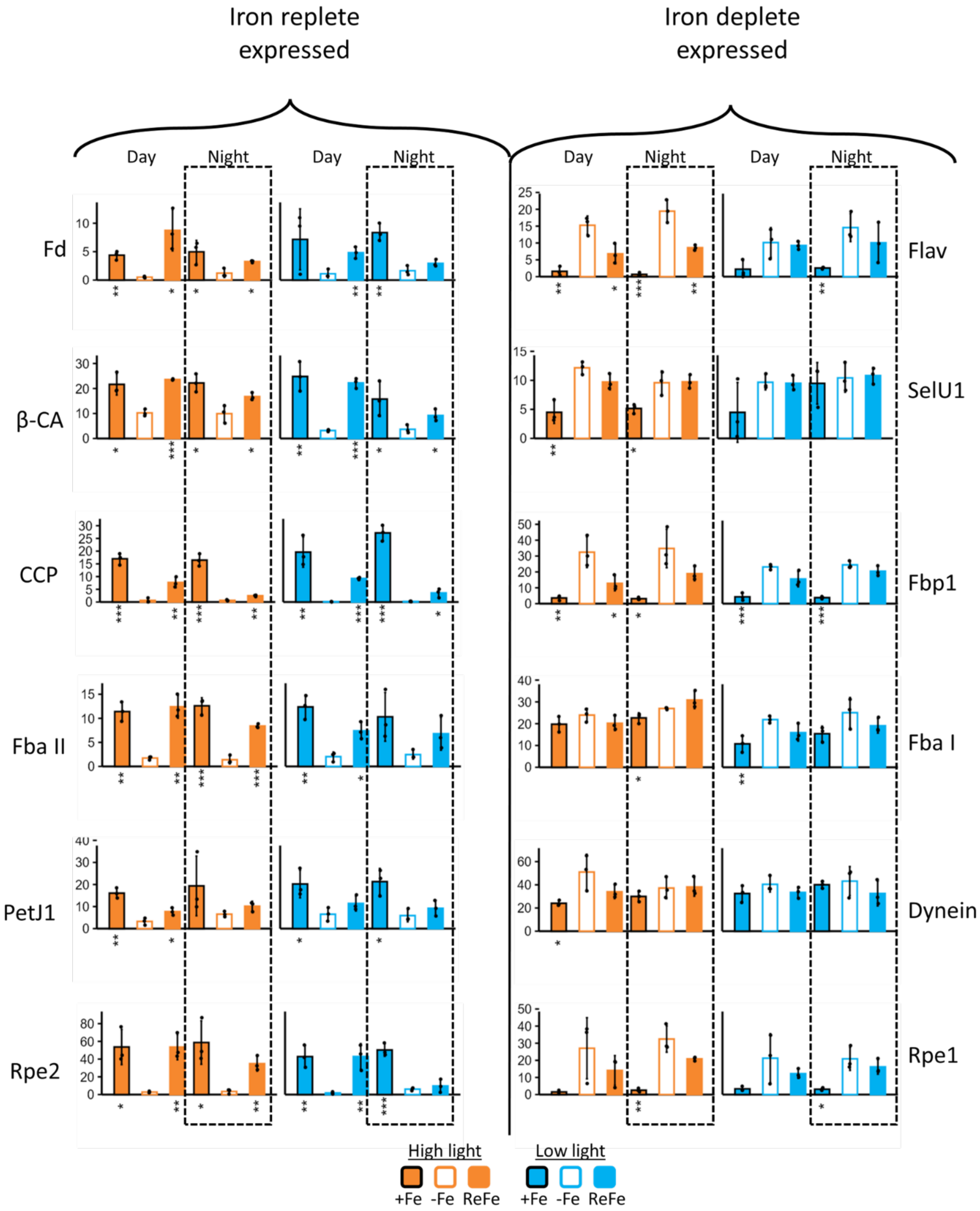

Supplemental Fig.10 Abundance of Fe-sensitive proteins in *P. calceolata*. Abundance of iron-sensitive proteins. For each protein, bars show the mean normalized abundance

(mean ± SD) for Day and Night samples under high light (HL) and low light (LL) (facets). Individual biological replicates (n=3) are overlaid as black points. Three iron conditions are shown (+Fe, −Fe, ReFe), with −Fe displayed as an open bar and +Fe/ReFe as filled bars. Vertical asterisks indicate significant differences relative to the −Fe reference within the same light regime and time period (*=$p < 0.05$, **=<0.01, ***=<0.001,two-sided unpaired t-tests, equal variance) difference from -Fe value at same time of day. Fd = ferredoxin, Fbp1 = fructose 1,6-bisphosphatase, CCP = cytochrome c peroxidase (mitochondrial), β-CA = β-carbonic anhydrase, SelU1 = selenoprotein U1, Fbp1 = fructose 1,6-bisphosphatase, Fba = fructose bisphosphate aldolase, PetJ1= cytochrome c_6, Rpe = ribulose 5-phosphate 3-epimerase, Flav = flavodoxin. Source data are provided as a Source Data file.

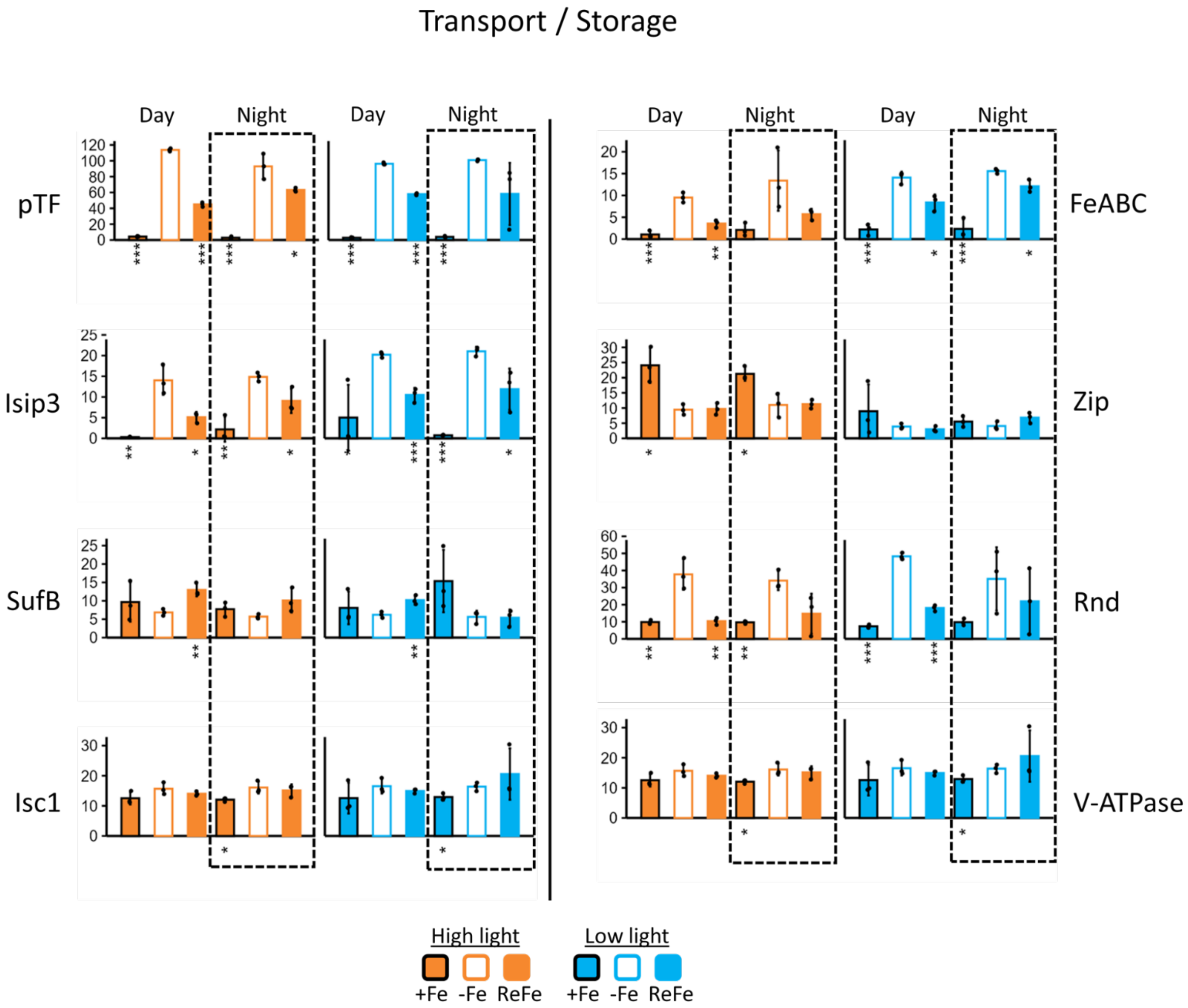

Supplemental Fig.11 Abundance of Fe-related proteins in *P. calceolata*. Scaled abundance of iron transport and storage related proteins. For each protein, bars show the mean normalized abundance (mean ± SD) for Day and Night samples under high light (HL) and low light (LL) (facets). Individual biological replicates (n=3) are overlaid as black points. Three iron conditions are shown (+Fe, −Fe, ReFe), with −Fe displayed as an open bar and +Fe/ReFe as filled bars. Vertical asterisks indicate significant differences relative to the −Fe reference within the same light regime and time period (*=p < 0.05, **=<0.01, ***=<0.001,two-sided unpaired t-tests, equal variance) difference from -Fe value at same time of day. pTF = phytotransferrin, Isip3 = iron starvation induced protein 3, FeABC = ferrichrome ABC transporter protein, Zip = zinc responsive transporter/iron responsive transporter-like protein, Mrp1 = multidrug resistance protein, SufB = chloroplast Fe-S

cluster assembly protein, V-ATPase = vacuolar proton pump, Isc1 = Iron sulfur cluster assembly protein, Rnd = resistance-nodulation-division transporter. Source data are provided as a Source Data file.

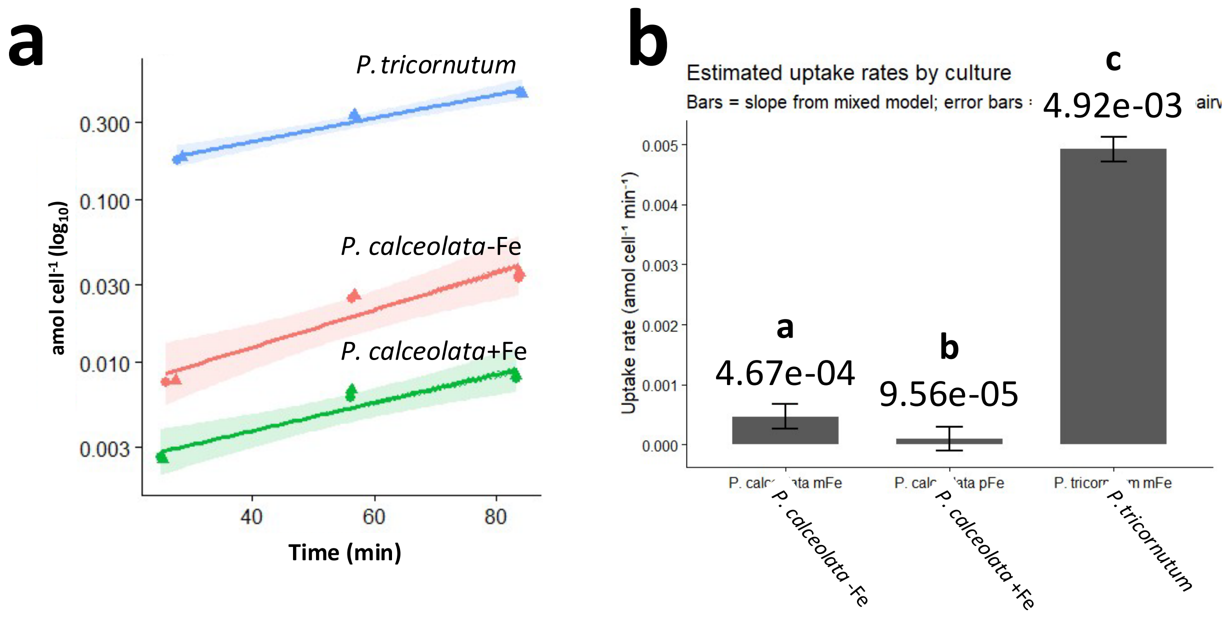

c

Contrast	Slope difference	SE	df	t-ratio	P-value
(*P. calceolata* -Fe) - (*P. calceolata* +Fe)	3.72E-04	1.19E-04	10.0016	3.123324	0.0267
(*P. calceolata* -Fe) - (*P. tricornutum*)	-0.00445	1.21E-04	10.00141	-36.7395	2.74E-11
(*P. calceolata* +Fe) - (*P. tricornutum*)	-0.00483	1.20E-04	10.00006	-40.1411	1.32E-11

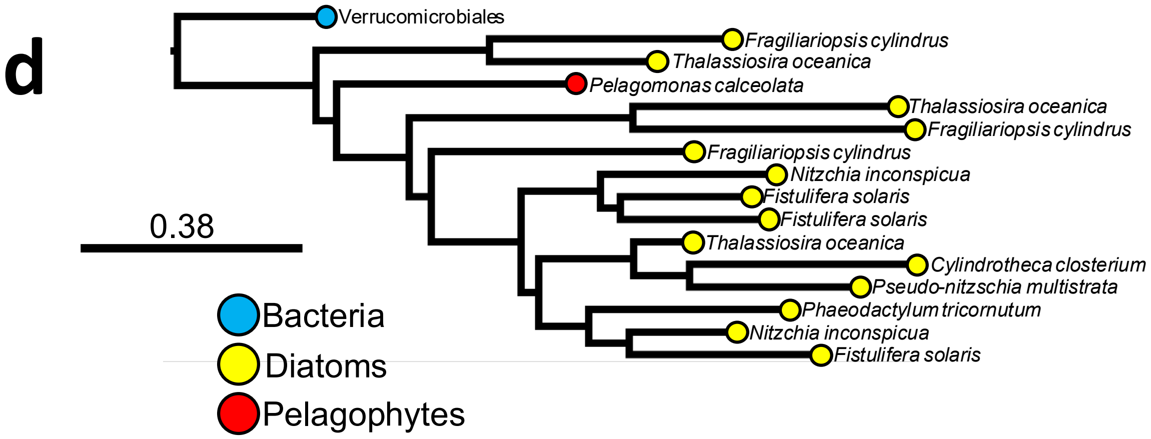

Supplemental Fig 12. Siderophore Fe acquisition by *P. calceolata*. a, Time-course of intracellular iron accumulation (amol Fe cell^{-1}) for each culture treatment, after addition of 250 pM ferrioxamine b. Points represent biological replicates, and lines show linear regressions (±95 % confidence intervals). The slope of each line corresponds to the uptake rate. Cultures include *P. calceolata* under Fe-replete (+Fe) and Fe-limited (-Fe) conditions, and *P. tricornutum* under Fe-limited conditions (15 pM Fe'). **b,** Culture-specific uptake rates (amol Fe cell^{-1} min^{-1}) estimated from a linear mixed-effects model that included random slopes and intercepts for replicate cultures. Bars show slope estimates (±95 % CI)

from the model; letters indicate groups not significantly different in pairwise comparisons (Tukey adjustment, $\alpha = 0.05$). **c,** Estimated differences in slopes (uptake rates) between cultures from Tukey-adjusted pairwise contrasts of the mixed-effects model. Positive values indicate higher uptake rates in the first culture listed in each contrast. **d,** Phylogenetic placement of the *P. calceolata* siderophore-binding protein candidate. Distance tree of BLASTP hits generated with NCBI's "Distance tree of results" using default parameters from the nr database. Branch tips are labeled with organism names; the *P. calceolata* sequence (maker-scf300000870743.0-snap-gene-6.21-mRNA-1) is indicated in red. Branch lengths reflect sequence divergence (substitutions per site) as computed by NCBI's pipeline. Source data are provided as a Source Data file.

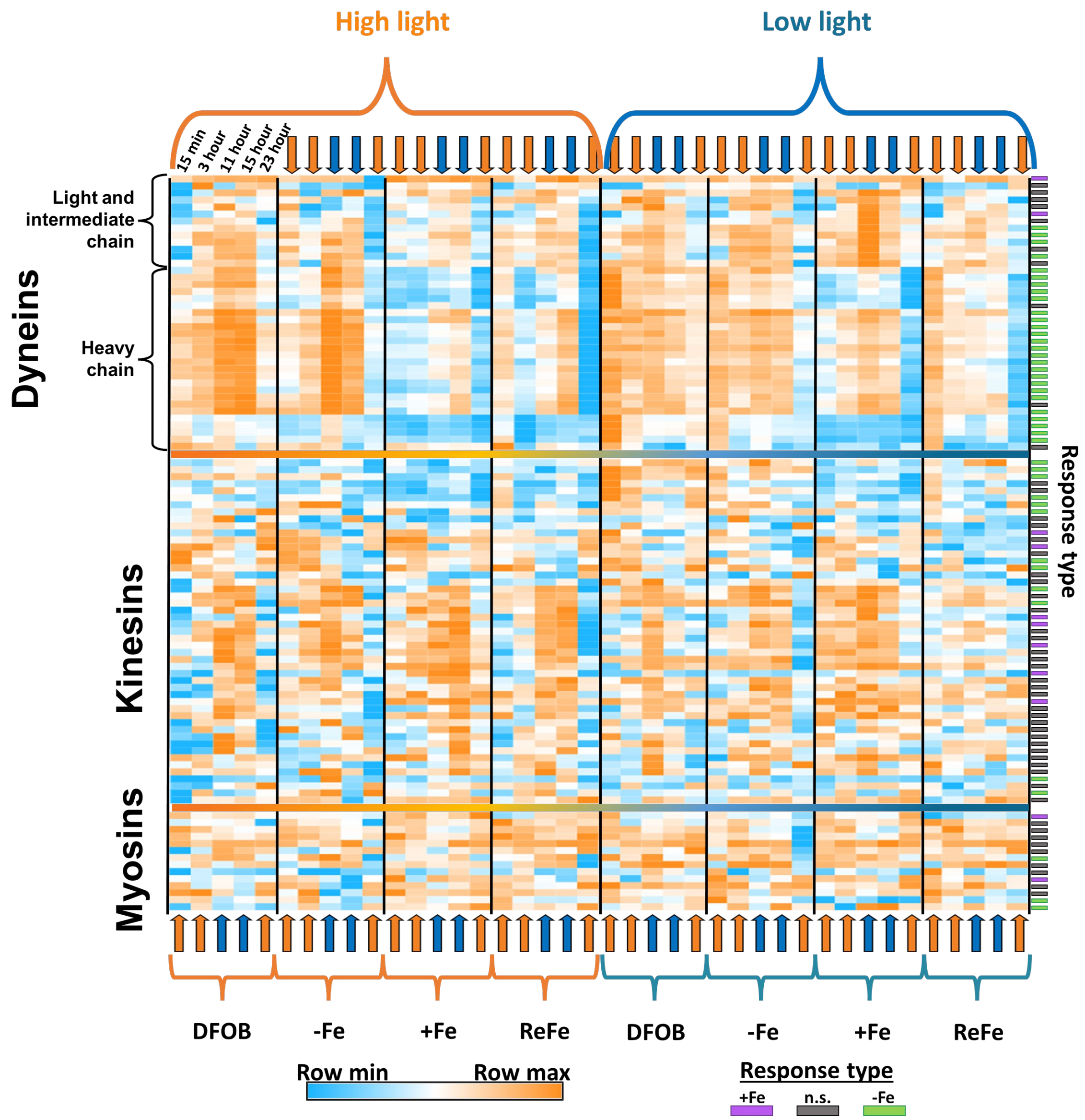

Supplemental Fig.13 Motor protein gene expression with respect to Fe/light co-limitation in *P. calceolata*. Dynein, kinesin and myosin motor protein gene expression across all conditions and time points. Heatmap showing average CPM transcript abundance of genes annotated as dynein, kinesin or myosin across the diel cycle and hierarchically clustered by expression pattern (Pearson correlation). Fe-related response types (Supplementary Data 11) are given by colored bar. Source data are found in Supplementary Data 2 and 3.

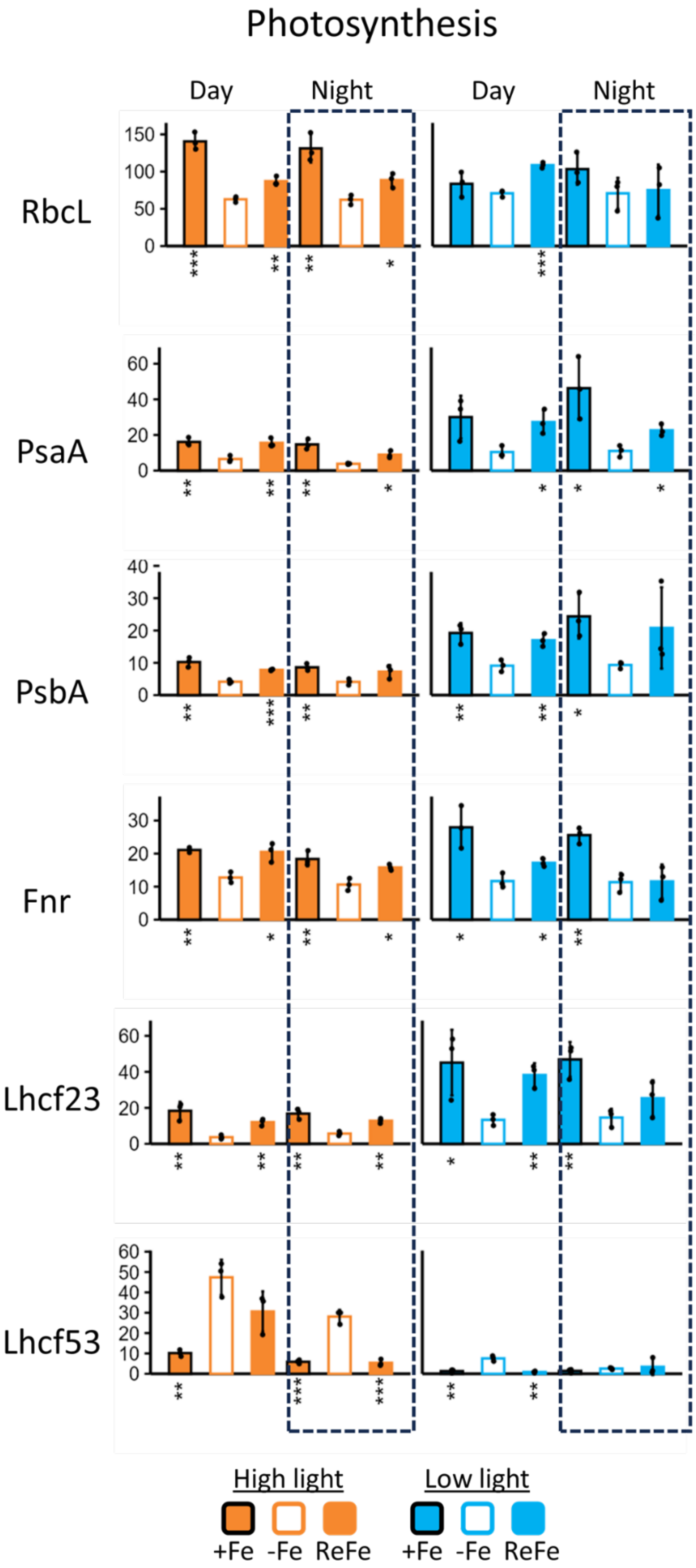

Supplemental Fig.14 Changes in photosynthesis-related protein abundance in P. calceolata. Scaled abundance of photosynthetic proteins. For each protein, bars show

the mean normalized abundance (mean ± SD) for Day and Night samples under high light (HL) and low light (LL) (facets). Individual biological replicates (n=3) are overlaid as black points. Three iron conditions are shown (+Fe, −Fe, ReFe), with −Fe displayed as an open bar and +Fe/ReFe as filled bars. Vertical asterisks indicate significant differences relative to the −Fe reference within the same light regime and time period (*=p < 0.05, **=<0.01, ***=<0.001,two-sided unpaired t-tests, equal variance) difference from -Fe value at same time of day. RbcL = ribulose 1,5 bisphosphate carboxylase large subunit, PsaA = photosystem I reaction center proteins A, PsbA = photosystem II reaction center proteins A, Lhcf = light harvesting complex protein f, Fnr = ferredoxin NADP+ oxidoreductase. Source data are provided as a Source Data file.

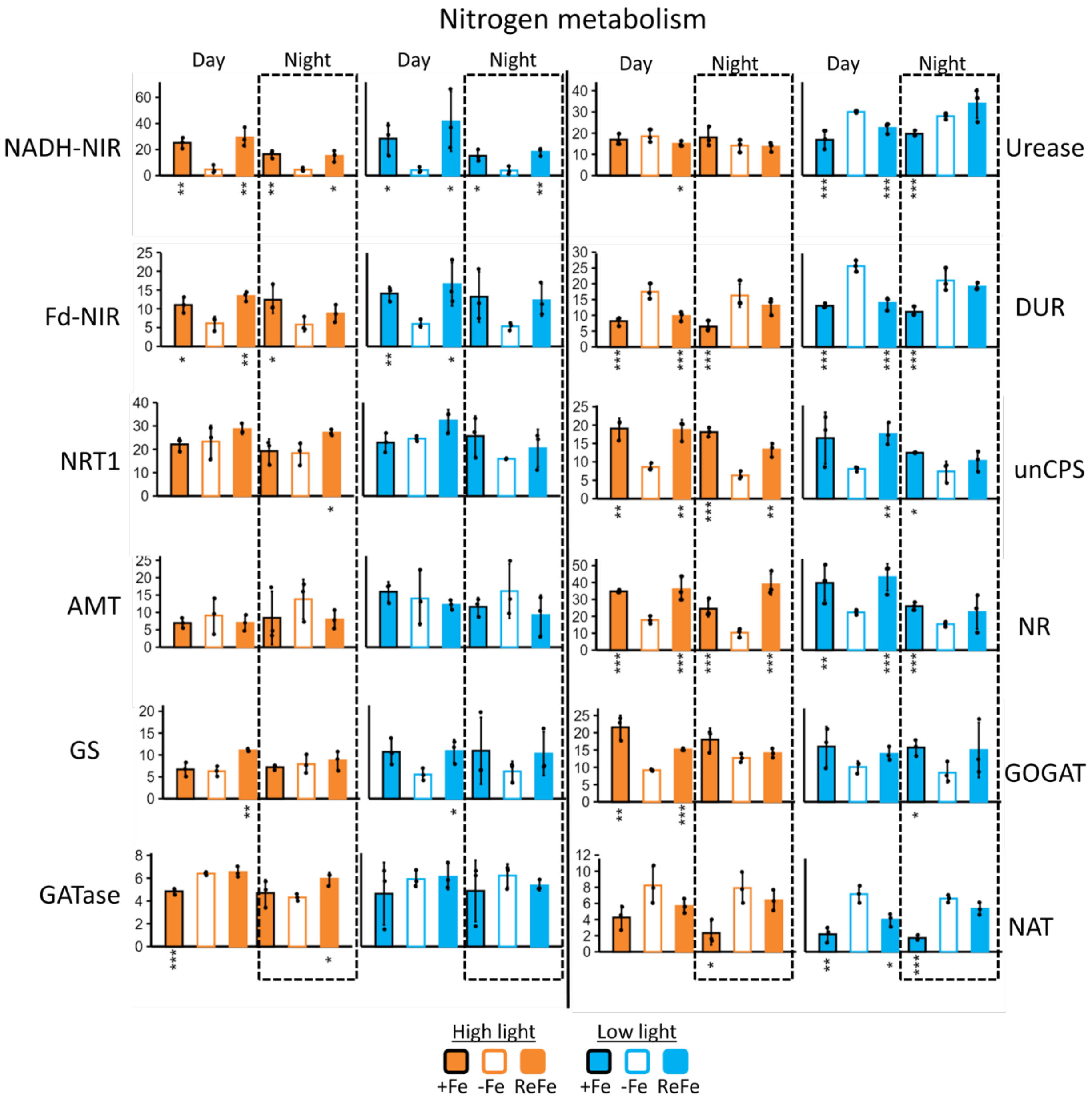

Supplemental Fig. 15 Impacts of Fe/light co-limitation on abundance of nitrogen metabolism proteins in *P. calceolata*. Scaled abundance of nitrogen metabolism proteins. For each protein, bars show the mean normalized abundance (mean ± SD) for Day and Night samples under high light (HL) and low light (LL) (facets). Individual biological replicates (n=3) are overlaid as black points. Three iron conditions are shown (+Fe, −Fe, ReFe), with −Fe displayed as an open bar and +Fe/ReFe as filled bars. Vertical asterisks indicate significant differences relative to the −Fe reference within the same light regime and time period (*=p < 0.05, **=<0.01, ***=<0.001,two-sided unpaired t-tests, equal variance) difference from -Fe value at same time of day. Nadh-Nir = NADPH nitrite reductase, NAT = nucleobase-ascorbate Transporter, Fd-Nir = ferredoxin nitrite reductase,

Nrt1 = nitrate transporter, Amt = ammonia transporter, GsIII = glutamine synthetase III, GATase = glutamine amidotransferases, Uncps = carbamoyl phosphate synthetase, Nr = nitrate reductase, Gogat = glutamine oxoglutarate aminotransferase. Source data are provided as a Source Data file.

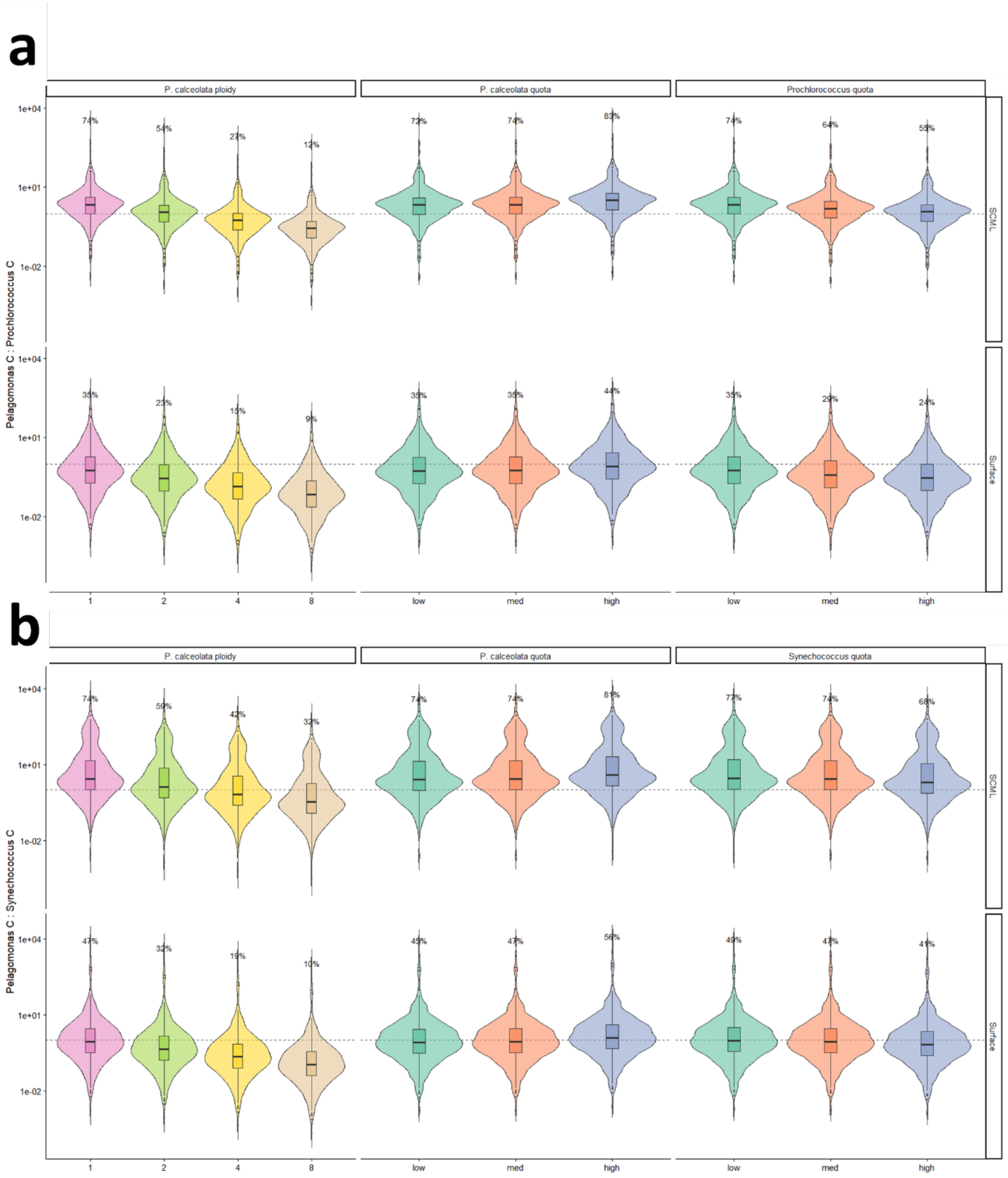

Supplemental Fig 16. Sensitivity of *P. calceolata* carbon estimates to cellular carbon quota and ploidy assumptions. a, Ratios of estimated *P. calceolata* carbon biomass to *Prochlorococcus* carbon biomass under varying assumptions of *P. calceolata* carbon quota, *Prochlorococcus* carbon quota, or *P. calceolata* ploidy. **b,** Same analysis for Synechococcus. Violin plots show the distribution of ratios across all stations within each depth category (surface or subsurface chlorophyll maximum layer), with dashed lines indicating a 1:1 ratio. Each subplot represents a different sensitivity scenario: (i) varying the

assumed *P. calceolata* genome ploidy (1–8 genomes cell^{-1}, with two 18S rDNA copies per genome), (ii) varying the *P. calceolata* cellular carbon content (low = 1200 fg C cell^{-1}, med = 1251 fg C cell^{-1}, high = 1800 fg C cell^{-1}); (iIi) varying the cyanobacterial carbon quota (Prochlorococcus: low = 32 fg C cell^{-1}, med = 45.8 fg C cell^{-1}, high = 60.9 fg C cell^{-1}; Synechococcus: low = 92.4 fg C cell^{-1}, med = 101 fg C cell^{-1}, high = 132 fg C cell^{-1}); Pelagomonas cell abundances were inferred from 18SV9 copy numbers using these assumptions, and total particulate carbon per liter was calculated for each scenario. Percentages above violins indicate the fraction of samples where *P. calceolata* carbon exceeded that of the corresponding cyanobacterium. Ratios are plotted on a $\log_{10}$ scale. Source data are provided as a Source Data file.

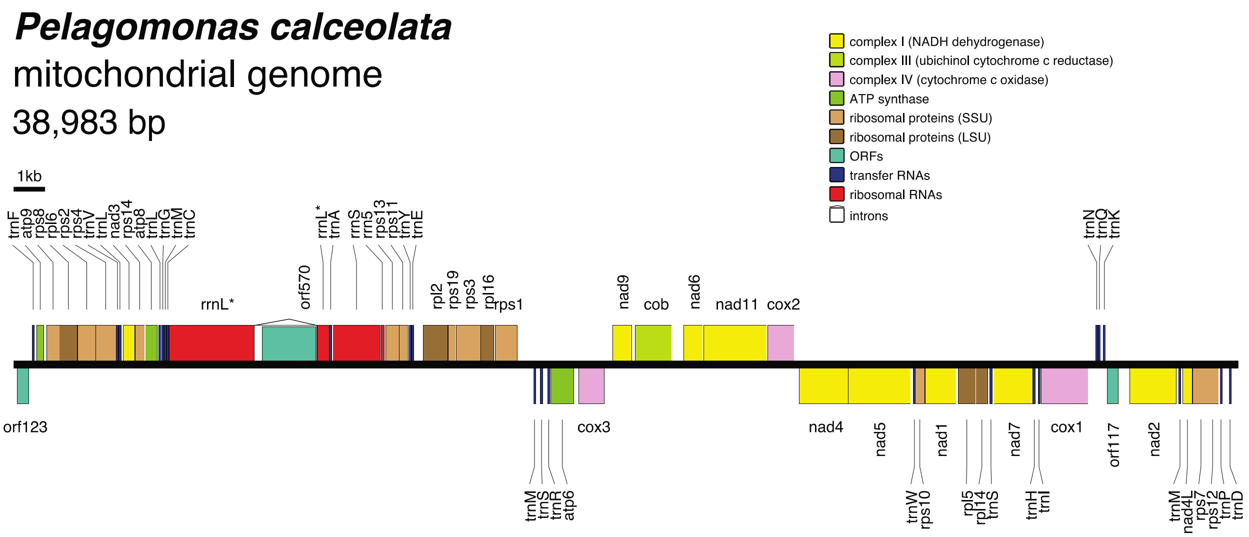

Supplementary Fig. 17. **Map of the *P. calceolata* mitochondrial genome.** The 38,983 bp mitochondrial genome is shown as a linear map with genes drawn as colored boxes along the genome; gene orientation is indicated by the position above/below the black line. Colors denote functional categories, including respiratory chain complexes, ATP synthase subunits, small and large ribosomal proteins, other open reading frames (ORFs), transfer RNAs, ribosomal RNAs, and introns (see legend). The 1 kb scale bar indicates genomic distance.

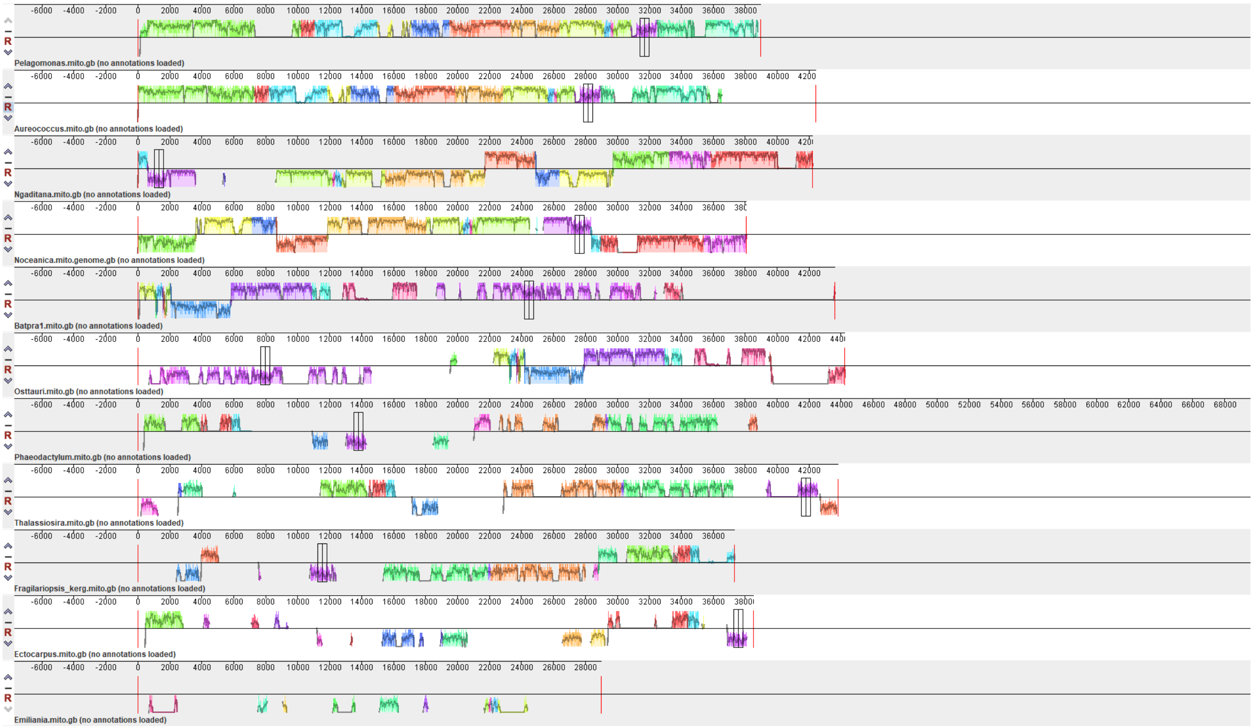

Supplementary Fig. 18.

Comparative MAUVE alignment of the *P. calceolata* mitochondrial genome with other phytoplankton. Locally collinear blocks identified by MAUVE are shown as colored segments, with each color representing homologous genomic regions shared among the mitochondrial genomes of *P. calceolata* and the comparison taxa (*Aureococcus anophagefferens, Nannochloropsis gaditana, Nannochloropsis oceanica, Baythycoccus prasinos, Ostreococcus tauri, Phaeodactylum tricornutum, Thalassiosira pseduonana, Fragilariopsis kerguelensis, Ectocarpus siliculosus, and Gephyrocapsa huxleyi*). Blocks plotted above and below the center line indicate regions in the forward and reverse orientations, respectively, highlighting inversions and rearrangements.

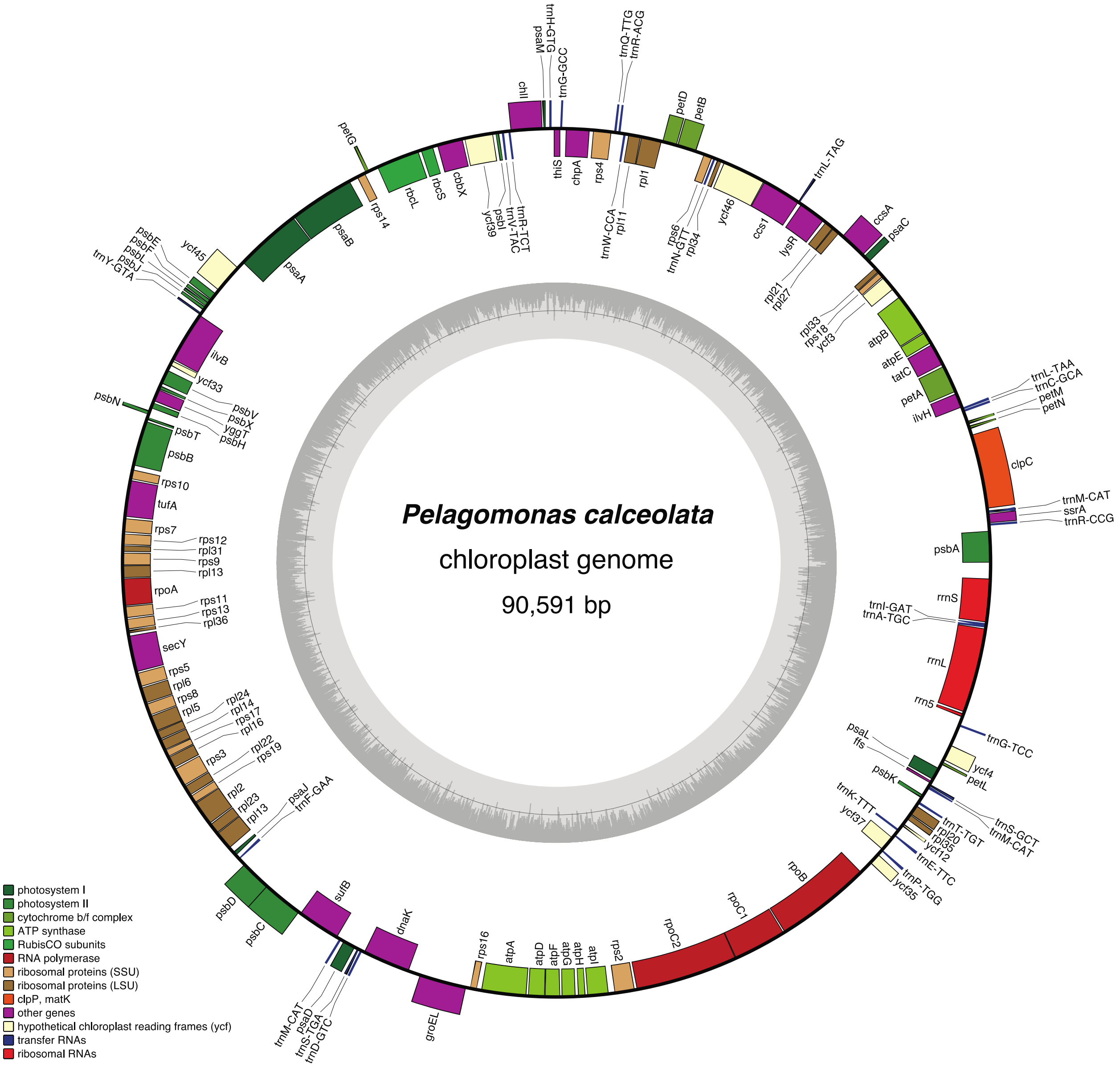

Supplementary Fig. 19. Circular map of the *P. calceolata* chloroplast genome. The 90,591 bp chloroplast genome is shown as a circle, with genes drawn on the outer and inner tracks according to transcriptional orientation. Genes are colored by functional category (see legend). The inner grey ring depicts variation in GC content along the genome.

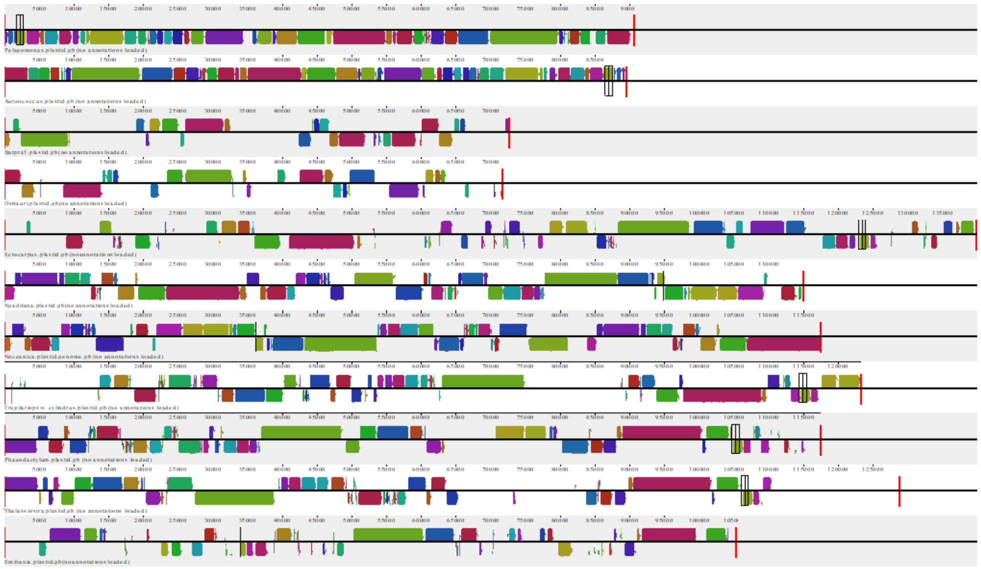

Supplementary Fig. 20. Comparative progressiveMauve alignment of the P. calceolata plastid genome with other marine phytoplankton. Locally collinear blocks identified by progressiveMauve are shown as coloured segments, with each colour representing homologous genomic regions shared among the plastid genomes of P. calceolata and the comparison taxa (*Aureococcus anophagefferens, Nannochloropsis gaditana, Nannochloropsis oceanica, Baythycoccus prasinos, Ostreococcus tauri, Phaeodactylum tricornutum, Thalassiosira pseduonana, Fragilariopsis kerguelensis, Ectocarpus siliculosus, and Gephyrocapsa huxleyi*). Blocks plotted above and below the center line indicate regions in the forward and reverse orientations, respectively, highlighting inversions and rearrangements.

Supplementary Table 1. Light interaction summary across data types. Counts of features classified as positive, negative, or n.s. (not significant) for physiology traits (analyzed per time point: 3, 15, 23 hr), RNA transcripts, and proteins. “Positive” indicates a synergistic Fe x Light interaction (excess over additivity > 0); “negative” indicates antagonism/buffering (excess over additivity < 0); “n.s.” indicates FDR ≥ 0.05 for the interaction term. For physiology, the four cell means (HL +Fe, LL +Fe, HL -Fe, LL -Fe) were modeled per trait/timepoint; for RNA (edgeR) and protein (limma), the interaction was estimated from factorial models.

	**Physiology**	**Transcriptomics**	**Proteomics**
**Positive**	6	32	10
**Negative**	4	110	32
**n.s.**	20	9099	2551
