# Supplementary material for: Molecular and physiological acclimation to low light and iron scarcity in a globally abundant oceanic pelagophyte
Source: Nat Commun. 2026 Apr 20;17:5480. doi: 10.1038/s41467-026-71628-0 (PMC13284251; doi:10.1038/s41467-026-71628-0)
Supplement: Supplementary file 2 — Descriptions of Additional Supplementary Files [file 41467_2026_71628_MOESM2_ESM.pdf]

Descriptions of Additional Supplementary Files

Supplementary Data 1: P. calceolata physiology data from Fe/light co-limitation experiment including cell concentrations, chlorophyll a, C, N, Fe, Cu and protein cellular contents.

Supplementary Data 2: P. calceolata transcriptomic data. Counts and CPM of P. calceolata transcripts from Fe/light co-limitation experiment.

Supplementary Data 3: Annotations and abbreviations for P. calceolata gene models.

Supplementary Data 4: Fasta file of P. calceolata gene model coding sequences.

Supplementary Data 5: Fasta file of P. calceolata gene model amino acid sequences.

Supplementary Data 6: edgeR comparisons made using transcriptomic data.

Supplementary Data 7: Gene membership and GO enrichment in WGCNA modules.

Supplementary Data 8: Proteomics intensities (normalized and imputed).

Supplementary Data 9: Proteomics DE analysis via limma.

Supplementary Data 10: Results of Fe x Light interaction test - physiological parameters.

Supplementary Data 11: Results of Fe x Light interaction test - transcriptomics.

Supplementary Data 12: Results of Fe x Light interaction test - proteomics.

Supplementary Data 13: P. calceolata dyneins - class, Fe sensitivity, and protein sequence.

Supplementary Data 14: Environnmental data from the NCOG project.

Supplementary Data 15: Transcriptomics response types determine with edgeR.

Supplementary Data 16: Results of imputation analysis of proteomics data.

.
