# Supplementary material for: Molecular and physiological acclimation to low light and iron scarcity in a globally abundant oceanic pelagophyte
Source: Nat Commun. 2026 Apr 20;17:5480. doi: 10.1038/s41467-026-71628-0 (PMC13284251; doi:10.1038/s41467-026-71628-0)
Supplement: Supplementary file 19 — Reporting Summary [file 41467_2026_71628_MOESM19_ESM.pdf]

nature portfolio

Corresponding author(s): Andrew Allen

Last updated by author(s): Feb 24, 2026

Reporting Summary

Nature Portfolio wishes to improve the reproducibility of the work that we publish. This form provides structure for consistency and transparency in reporting. For further information on Nature Portfolio policies, see our Editorial Policies and the Editorial Policy Checklist.

Statistics

For all statistical analyses, confirm that the following items are present in the figure legend, table legend, main text, or Methods section.

n/a	Confirmed	
☐	☒	The exact sample size (*n*) for each experimental group/condition, given as a discrete number and unit of measurement
☐	☒	A statement on whether measurements were taken from distinct samples or whether the same sample was measured repeatedly
☐	☒	The statistical test(s) used AND whether they are one- or two-sided<br>*Only common tests should be described solely by name; describe more complex techniques in the Methods section.*
☐	☒	A description of all covariates tested
☐	☒	A description of any assumptions or corrections, such as tests of normality and adjustment for multiple comparisons
☐	☒	A full description of the statistical parameters including central tendency (e.g. means) or other basic estimates (e.g. regression coefficient) AND variation (e.g. standard deviation) or associated estimates of uncertainty (e.g. confidence intervals)
☐	☒	For null hypothesis testing, the test statistic (e.g. *F*, *t*, *r*) with confidence intervals, effect sizes, degrees of freedom and *P* value noted<br>*Give P values as exact values whenever suitable.*
☒	☐	For Bayesian analysis, information on the choice of priors and Markov chain Monte Carlo settings
☐	☒	For hierarchical and complex designs, identification of the appropriate level for tests and full reporting of outcomes
☐	☒	Estimates of effect sizes (e.g. Cohen's *d*, Pearson's *r*), indicating how they were calculated

Our web collection on statistics for biologists contains articles on many of the points above.

Software and code

Policy information about availability of computer code

Data collection

Fv/Fm data was collected using WinControl software, flow cytometry data was collected using Cell LabQuanta SC software

Data analysis

Data analysis was performed using RStudio 2025.05.0, and Microsoft Excel Version 2505 Build 16.0.18827.20102, and publicly available R packages described in the text, including:
R / Bioconductor packages:
- BiocManager (1.30.26)
- edgeR (4.2.2)
- limma (3.60.6)
- WGCNA (1.73)
- GO.db (3.19.1)
- AnnotationDbi (1.66.0)
- impute (1.78.0)
- preprocessCore (1.66.0)
- matrixStats (1.5.0)
- readxl (1.4.5)
- readr (2.1.5)
- dplyr (1.1.4)
- tidyr (1.3.1)
- stringr (1.5.2)
- ggplot2 (4.0.0)
- ggpattern (1.2.1)

- patchwork (1.3.2)
- pheatmap (1.0.13)
- janitor (2.2.1)
- purrr (1.1.0)
- tidyselect (1.2.1)

Custom code for transcriptomic and proteomic data analysis is available in the Zenodo repository: 10.5281/zenodo.18718641 [https://zenodo.org/records/18718641]

For manuscripts utilizing custom algorithms or software that are central to the research but not yet described in published literature, software must be made available to editors and reviewers. We strongly encourage code deposition in a community repository (e.g. GitHub). See the Nature Portfolio guidelines for submitting code & software for further information.

Data

Policy information about availability of data

All manuscripts must include a data availability statement. This statement should provide the following information, where applicable:

- Accession codes, unique identifiers, or web links for publicly available datasets
- A description of any restrictions on data availability
- For clinical datasets or third party data, please ensure that the statement adheres to our policy

All data are provided within the text, Supplementary Information, and Zenodo repository (10.5281/zenodo.18718641 [https://zenodo.org/records/18718641] Supplementary Data 1-16). Genomic data are deposited with the NCBI accession JBNOXR000000000 and transcriptomic data are included in BioProject PRJNA193556. Proteomic data are available in ProteomeXchange via the PRIDE database and available at the DOI:10.6019/PXD065159.

Research involving human participants, their data, or biological material

Policy information about studies with human participants or human data. See also policy information about sex, gender (identity/presentation), and sexual orientation and race, ethnicity and racism.

Reporting on sex and gender	Not applicable, no human participants or human data.
Reporting on race, ethnicity, or other socially relevant groupings	Not applicable, no human participants or human data.
Population characteristics	Not applicable, no human participants or human data.
Recruitment	Not applicable, no human participants or human data.
Ethics oversight	Not applicable, no human participants or human data.

Note that full information on the approval of the study protocol must also be provided in the manuscript.

Field-specific reporting

Please select the one below that is the best fit for your research. If you are not sure, read the appropriate sections before making your selection.

☒ Life sciences ☐ Behavioural & social sciences ☐ Ecological, evolutionary & environmental sciences

For a reference copy of the document with all sections, see nature.com/documents/nr-reporting-summary-flat.pdf

Life sciences study design

All studies must disclose on these points even when the disclosure is negative.

Sample size	No sample size calculations were performed and sample sizes were maximized according to incubator space, time required for sample collection, and culture volume. No a priori statistical power calculation was performed because expected effect sizes and variance were not known in advance for these assays/organisms and because sample throughput was constrained by culture and instrument capacity. Sample sizes were chosen based on (i) standard practice in comparable physiology and “omics” experiments, (ii) feasibility of running independent biological replicates across all treatments and timepoints, and (iii) ensuring sufficient replication to support the planned statistical analyses (e.g., linear modeling with multiple-testing correction for high-dimensional data). Across experiments, we used independent biological replicates (separate cultures/samples) per condition/timepoint.
Data exclusions	No collected data were excluded from the study.
Replication	All culture experiments were performed in triplicate cultures and all attempts at replication were successful.
Randomization	Cultures were randomly assigned to different treatments.

Blinding	Investigators were not blinded to group allocation during data collection or analysis. Blinding was not feasible because experimental groups were defined by overt culture conditions/treatments (e.g., distinct light, Fe, and DFOB conditions) that must be known to set up and maintain the experiments and to execute timepoint-specific sampling. To minimize bias, primary outcomes were based on objective instrument- or sequence-derived measurements, and all analyses were performed using standardized, scripted pipelines with pre-specified processing and statistical criteria applied uniformly across groups.

Reporting for specific materials, systems and methods

We require information from authors about some types of materials, experimental systems and methods used in many studies. Here, indicate whether each material, system or method listed is relevant to your study. If you are not sure if a list item applies to your research, read the appropriate section before selecting a response.

Materials & experimental systems

n/a	Involved in the study	
☒	☐	Antibodies
☐	☒	Eukaryotic cell lines
☒	☐	Palaeontology and archaeology
☐	☒	Animals and other organisms
☒	☐	Clinical data
☒	☐	Dual use research of concern
☒	☐	Plants

Methods

n/a	Involved in the study	
☒	☐	ChIP-seq
☐	☒	Flow cytometry
☒	☐	MRI-based neuroimaging

Eukaryotic cell lines

Policy information about cell lines and Sex and Gender in Research

Cell line source(s)	Pelagomonas calceolata CCMP1756, isolated from 30.8333°N -136.8333°W (North Pacific Central Gyre). Obtained from National Center for Marine Algae and Microbiota
Authentication	Cells were authenticated using epifluorescent microscopy and screened for bacteria using DAPI staining.
Mycoplasma contamination	Cell lines were not tested for Mycoplasma contamination
Commonly misidentified lines (See ICLAC register)	None were used.

Animals and other research organisms

Policy information about studies involving animals; ARRIVE guidelines recommended for reporting animal research, and Sex and Gender in Research

Laboratory animals	The study did not involve animals.
Wild animals	The study did not involve animals.
Reporting on sex	Not applicable. This study examined microorganisms and did not involve animals or human subjects; therefore, sex is not relevant to the experimental design or interpretation. No sex-based variables were measured or analyzed.
Field-collected samples	The study did not involve field-collected samples.
Ethics oversight	Not applicable. This study involved only microorganisms and did not include human participants, identifiable human data, vertebrate animals, or other organisms requiring institutional animal care/ethics review. Therefore, no ethical approval was required.

Note that full information on the approval of the study protocol must also be provided in the manuscript.

Plants

Seed stocks

Not applicable, no plants used in this study.

Novel plant genotypes

Not applicable, no plants used in this study.

Authentication

Not applicable, no plants used in this study.

Flow Cytometry

Plots

Confirm that:

☐ The axis labels state the marker and fluorochrome used (e.g. CD4-FITC).

☐ The axis scales are clearly visible. Include numbers along axes only for bottom left plot of group (a 'group' is an analysis of identical markers).

☐ All plots are contour plots with outliers or pseudocolor plots.

☐ A numerical value for number of cells or percentage (with statistics) is provided.

Methodology

Sample preparation

Culture was pipetted into a 96 well plate and run directly on the instrument. No preservative or sample preparation was used.

Instrument

Beckman Coulter Cell Lab Quanta SC 771917

Software

Cell LabQuanta SC software

Cell population abundance

No post-sort fractions were generated. P. calceolata cells represented 100% of cells in all samples.

Gating strategy

Cells were gated on forward scatter and chlorophyll autofluorescence in order to distinguish them from smaller, non-fluorescent debris in samples. Fluorescence and forward scatter values are not used in this study, only counts are reported and not flow cytometry plots are presented.

☐ Tick this box to confirm that a figure exemplifying the gating strategy is provided in the Supplementary Information.
