# Supplementary material for: Molecular and physiological acclimation to low light and iron scarcity in a globally abundant oceanic pelagophyte
Source: Nat Commun. 2026 Apr 20;17:5480. doi: 10.1038/s41467-026-71628-0 (PMC13284251; doi:10.1038/s41467-026-71628-0)
Supplement: Supplementary file 20 — Transparent Peer Review file [file 41467_2026_71628_MOESM20_ESM.pdf]

nature portfolio

Peer Review File

Molecular and physiological acclimation to low light and iron scarcity in a globally abundant oceanic pelagophyte

Corresponding Author: Professor Andrew Allen

This file contains all reviewer reports in order by version, followed by all author rebuttals in order by version.

Version 0:

Reviewer comments:

Reviewer #1

(Remarks to the Author)
This manuscript investigates the adaptive strategies employed by the marine alga Pelagomonas calceolata, one of the most abundant eukaryotic phytoplankton in the oceans, under simultaneous iron and light limitation. The authors cultured P. calceolata under factorial combinations of iron availability and light intensity in laboratory conditions to characterize its physiological and transcriptomic responses. The results demonstrate that P. calceolata can maintain growth at extremely low cellular iron quotas through the dynamic deployment of iron-sparing mechanisms. Moreover, by comparing these laboratory-based findings with environmental metatranscriptomic data from natural communities, the study reveals that Fe- and light-responsive genes are also highly expressed in situ, underscoring the ecological relevance of these responses. Collectively, the data suggest that P. calceolata is highly specialized for environments where both iron and light are limiting, emphasizing its potential role in marine carbon and nitrogen cycling under such conditions. The manuscript is generally well-written and logically structured. However, certain aspects could benefit from further elaboration or clarification. Below are specific suggestions:
1.Evidence for Fe/light co-limitation: Although the study is designed to simulate both iron and light limitation, further discussion on the combined effects of these two stressors, as opposed to their individual effects, would be valuable. For instance, is there a statistical interaction between iron supply and light intensity, indicating that low light exacerbates iron stress? Does the response of P. calceolata under simultaneous iron and light limitation exceed the additive effects of single-factor limitations? Additional analysis or discussion on the potential synergistic effects of iron–light co-limitation would strengthen the claim that this alga is specifically adapted to environments characterized by both constraints.
2. Interpretation of gene-specific responses: The upregulation of RND family transporters under iron limitation is interpreted as a mechanism for exporting non-iron metal ions. While this is a plausible hypothesis, it would be more convincing if supported by direct measurements of other metal quotas or transport activities. If possible, providing such supporting evidence would enhance the robustness of this interpretation.
3. Ecological success of P. calceolata: Although the data show that P. calceolata can maintain low cellular iron content and adjust gene expression under Fe/light stress, these traits alone may not fully explain its ecological dominance. Other factors not measured in this study—such as small cell size (and therefore high surface-area-to-volume ratio), reduced predation pressure, or the ability to utilize organic nutrients—could also contribute. If the species' ecological success is attributed mainly to its low-iron tolerance, this may oversimplify the complexity of its adaptive strategies. Additional discussion on these potential contributing factors, such as light quality, phosphorus availability etc.. or comparisons with other phytoplankton, would be valuable. Furthermore, labeling P. calceolata as a "co-limitation specialist" would be better supported by evidence of its competitive growth advantage under low Fe/low light conditions relative to other species, or at least by demonstrating sustained net growth under such conditions. While the data show survival under Fe limitation, they also reveal clear growth deceleration (e.g., decreased carbon quotas and requirement for Fe re-supplementation for recovery). The manuscript should elaborate on how such traits translate into competitive advantage.
4.Diurnal gene expression patterns: The observation that a large proportion of genes exhibit diurnal variation is a highlight point. However, the discussion lacks detail on whether P. calceolata possesses robust circadian regulation or whether Fe/light stress disrupts its diel rhythms. While nearly half of the transcriptome appears to be under diurnal control, only 14% of proteins show diel differences, suggesting regulation at the post-transcriptional level or other mechanisms. Although diel regulation is briefly referenced in the introduction, it is not revisited in the discussion. A more in-depth analysis could better showcase this novel aspect of the study.
Minor comments:
1.The experimental design is central to the study's conclusions and should be described in more detail at the start of results

section, rather than being confined to the Methods section.
2.how the two light intensities used were defined as "high light" and "low light"—are they based on natural conditions, or were they chosen based on light-response curve measurements?
3.In Figure 1C, why does the Fv/Fm value in the "+Fe" group continuously decline?
4.The claim that P. calceolata may perform the majority of nitrate assimilation in SCML communities is based on transcriptomic and proteomic data. This conclusion would be strengthened by physiological measurements.(if possible)
5.Growth monitoring was based on RFU. Since pigment content can vary under different light and iron conditions, this could affect the accuracy of the growth estimation?
6.Some references to supplementary materials appear as “Supplementary Table XX.” These should be corrected.
7.Ensure that all abbreviations, including gene and protein names, are defined upon first use.
8.The numbering of Extended Data Figures should follow their order of appearance in the text (e.g., Figure S9 is referenced before Figure S5).

Reviewer #2

(Remarks to the Author)
I read with interest the manuscript titled “Molecular and Physiological Acclimation to Low Light and Iron Scarcity in Globally Abundant Oceanic Pelagophyte”. This paper investigates the strategies of Pelagomonas calceolata to iron and light co-limitation, through physiological and gene expression analysis. The findings of the paper are well written and the analyses are robust and appropriate. Overall, this paper makes an important contribution to our understanding of algal biology.

With expertise in genome assembly, I have limited my comments to this aspect of the paper. The methods used are appropriate and well reported to aid reproducibility. Additionally:

L150 – Supplementary Table XX?
L385 – It would be good to detail the DNA extraction method thoroughly here, similar to the detail for RNA extraction at L455.
L397 – I can’t seem to find Figure S12 and 13. For me, the extended data Figures end at Extended Data Fig.10.

Reviewer #3

(Remarks to the Author)
- This manuscript presents an integrated physiological, transcriptomic, proteomic, and environmental analysis of Pelagomonas calceolata under iron (Fe) and light limitation, including short-term Fe resupply dynamics over a diel cycle. It reports exceptionally low Fe:C ratios, rapid Fe-sparing responses (e.g., flavodoxin for ferredoxin), distinct remodeling of Calvin-Benson cycle components under Fe stress, shifts in nitrogen source preference, and concordance between laboratory Fe/light response signatures and in situ metatranscriptomes from SCML communities in the California Current Ecosystem (CCE). Elemental quotas (C, N, Fe, Cu) and Chl a, together with diel RNA-seq and TMT proteomics, are used to support these claims.

- P. calceolata maintains low Fe:C ratios across treatments and rapidly increases Fe:C within hours of resupply due to fast Fe uptake relative to C restoration, demonstrating stringent Fe economy under both high and low light. These trends are supported by measured C, N, Fe quotas and derived Fe:C across time points.
- Under low Fe, Cu quotas and Cu:C increase, opposite to Fe trends, with Cu returning toward Fe-replete values after Fe resupply; authors interpret this as potential increased reliance on Cu proteins and/or non-specific metal uptake under Fe stress. Moreover, transcriptional responses are strongly structured by day/night and Fe availability; proteomic remodelling is driven more by Fe and light, with less diel variation than the transcriptome (43.9% of transcripts vs 14.3% of proteins differentially expressed by day/night) .
- Canonical Fe-sparing: flavodoxin is upregulated in low Fe (daytime-biased at the transcript level), replacing ferredoxin at the protein level; post-resupply, flavodoxin rapidly declines while ferredoxin increases .
- Fe acquisition machinery: phytotransferrin (pTF) and ISIP3 are upregulated at low Fe at both transcript and protein levels; a ferrichrome-like siderophore receptor is induced under low Fe and decreases upon resupply, consistent with capacity to access strong Fe ligands; ZIP transporters are more expressed in Fe replete cultures, suggesting intracellular roles beyond Fe uptake; RND family transporters are upregulated in low Fe and may mediate efflux of non-Fe metals .
- Photosynthesis and Calvin-Benson cycle: light-harvesting and PSI/PSII reaction center proteins increase in low light with Fe present; PSI/PSII balance is maintained across treatments; a switch from Class II to Class I fructose-bisphosphate aldolase (FBA), plus coordinated low-Fe expression of Fbp and Rpe, indicates rewiring of the regenerative phase under Fe stress .
- P. calceolata is abundant in the CCE (often millions of cells per liter) and frequently contributes more POC than Prochlorococcus or Synechococcus; genes identified as Fe- and light-responsive in culture are enriched in SCML metatranscriptomes, supporting in situ expression of these adaptations.

- The study advances mechanistic understanding of how a globally distributed picoeukaryote thrives at Fe/light co-limited SCMLs, linking cellular Fe economy, diel regulation, and N source flexibility to environmental niches and biogeochemical roles. The integration of controlled diel-resolved culture work with field metatranscriptomes at scale is a notable strength. While Fe-sparing modules (flavodoxin, pTF/ISIPs) and light effects on Fe demand are known in phytoplankton, this work

uniquely characterizes their coordinated, time-resolved deployment in P. calceolata under co-limitation and validates environmental relevance in the CCE. The observed switch in Calvin-Benson components (Class I vs II FBA, Fbp, Rpe) and the Fe-driven shift in N source preference in this taxon add new detail to eukaryotic picoalgal acclimation strategies .
- The manuscript situates results within prior observations of low Fe requirements in P. calceolata, Fe/light interactions in phytoplankton, and canonical Fe-stress machinery across taxa; it highlights differences from diatom models (e.g., urea cycle regulation, ferritin absence) and from diazotroph "hot-bunking," arguing P. calceolata prioritizes Fe sparing over storage .

The data support the conclusions since the low Fe:C and rapid Fe resupply responses in quotas and Fv/Fm are well documented across replicates and time points. Fe-sparing via flavodoxin-for-ferredoxin substitution and induction of Fe acquisition pathways (pTF, ISIP3) are robust at both transcript and protein levels and revert with Fe addition .
Downregulation of nitrate/nitrite reduction proteins at low Fe and upregulation of urea transporter/urease supports a plausible shift in N source usage under Fe stress .
I would highly suggest some revision to temper claims or add evidence in the lack of plastocyanin-for-cytochrome c6 substitution: evidence is mostly from transcript patterns (both upregulated in high Fe) and non-detection of plastocyanin in proteomes; given possible limits of proteome coverage, more direct quantification (e.g., targeted proteomics) would better support this negative result .
Moreover there is "No evidence of Fe hot-bunking": the absence of pronounced diel oscillation in light-reaction proteins suggests stable investment, but does not strictly exclude nighttime Fe recycling; this conclusion should be framed cautiously as "no evidence observed in our proteome time points" .
Dynein upregulation indicates a role under low Fe/high light but functional assignment to motility or osmotrophy remains inferential; additional phenotyping (e.g., flagellar motility assays) would strengthen this link. Siderophore uptake inference from ferrichrome-like receptor expression and growth under DFOB is suggestive; direct uptake assays or radiotracer experiments with defined siderophores would make this claim more definitive .

Methodology and analysis: strengths and points needing clarification
- Strengths:
- Clean trace-metal culturing in Aquil with explicit Fe speciation calculations (Fe′) and chelator perturbations; acclimation for >10 generations prior to experiments; diel sampling design with biological triplicates under two light regimes .
- Quantitative elemental quotas (CHN analyzer; ICP-MS with oxalate-EDTA rinse) and chlorophyll by standard fluorometry provide solid physiological grounding .
- Transcriptomics: ribodepletion, consistent library prep, edgeR GLM across factors; clear day/night and Fe/light separation in PCA/UMAP; response-type assignments are systematic .
- Proteomics: TMT multiplexing, rigorous filtering, normalization (including TMM), and edgeR downstream statistics; alignment to a curated genome assembly and gene models of P. calceolata .
- Environmental mapping: taxonomic assignment via PhyloDB, DIAMOND mapping to the genome, normalization with DESeq2; quantitative 18S-based cell abundance estimates anchored to internal standards .
The paper needs more clarifications on the multiple testing control: The manuscript refers to $p<0.05$ thresholds in edgeR without explicitly stating FDR procedures for transcript or protein DE; please report FDR-adjusted criteria and the number of DE features under specified FDR (e.g., 5%) for each contrast . Proteomics data handling: Seven low-abundance samples were removed and missing values imputed at half row-minimum prior to edgeR. Please demonstrate robustness of key conclusions to alternative imputation strategies and to inclusion/exclusion of these samples (sensitivity analysis), and justify any potential biases introduced by the filtering step .
Siderophore/DFOB inferences: You calculate Fe′ ~5.6 fM with 100 nM DFOB and infer uptake of strongly bound Fe; consider including direct growth rate comparisons and/or uptake assays to substantiate siderophore-based acquisition beyond expression profiles .
Plastocyanin vs c6: Given non-detection can be due to proteomic coverage, targeted quantification (PRM/SRM) or spectral counting statistics would clarify whether plastocyanin is truly absent at low Fe/high Cu, or below detection .
Metatranscriptome mapping specificity: Cross-mapping among pelagophytes can inflate apparent P. calceolata signal. Please provide mapping stringency, unique vs multi-hit handling, and estimates of potential cross-mapping. A sensitivity test restricting to diagnostic genes would increase confidence in the environmental enrichment results .
POC contribution estimates: Field C per cell is inferred from the average of culture quotas (104.3 fmol C $cell^{-1}$) applied across diverse environments; please include uncertainty ranges (e.g., based on condition-specific quotas from your experiments) and explore how SCML vs surface conditions alter the estimates .
18S copy number assumption: The conversion to cells assumes two 18S copies per cell; discuss potential copy-number variability among wild P. calceolata clades and its impact on absolute abundance estimates (provide sensitivity bounds) .
Statistical reporting for physiological comparisons: Extended Data figures show letters for groupings and Welch's t-tests for some pairwise contrasts. Please specify the full multiple-comparison framework used (e.g., ANOVA with post-hoc tests), correction for multiple testing, and exact n for each comparison .

- Methods are generally detailed and meet field standards for trace-metal culture, sequencing, and proteomics, with sufficient detail to replicate culturing, physiological measurements, and sequencing workflows . The Fe speciation calculation parameters are given; providing the exact computation workflow (spreadsheet or code) would further enhance reproducibility .
I would hihly recommend providing the scripts or detailed parameter files (e.g., edgeR model formulas and contrasts, normalization steps, imputation code) to fully reproduce the RNA-seq and proteomics statistical analyses .

- I do not see fatal flaws. The core experimental design and multi-omics integration are strong. However, the missing supplementary items/data access and some analytical clarifications need to be addressed before acceptance. A moderate revision focusing on:

- Completing and depositing all supplementary figures/tables/data,
- Clarifying statistical controls and robustness of proteomics inferences,
- Tempering or reinforcing a few mechanistic claims with additional analysis,
would make the manuscript publication-ready .

Specific requests for revision
1) Provide complete supplementary materials (all referenced Extended Data Figs 8–10 and finalized Supplementary Tables) and update data availability with proteomics repository accessions.
2) Detail multiple testing corrections (FDR) for all DE analyses (transcript and protein); report thresholds and DE counts per contrast; include MA/volcano plots per major contrast in the supplementary material.
3) Proteomics robustness: include sensitivity analyses for imputation/filtering choices; report peptide counts/coverage for plastocyanin and cytochrome c6 to support the "no substitution" inference or temper the claim .
4) Siderophore uptake: either add a brief growth/uptake validation under DFOB or explicitly frame this as a hypothesis consistent with expression patterns rather than a demonstrated capability .
5) Environmental mapping specificity: report unique mapping rates, handling of multi-mappers, and include a conservative analysis based on diagnostic gene sets to confirm SCML enrichment patterns .
6) POC estimates and 18S copy number: add uncertainty bounds/sensitivity analyses for C quotas and rDNA copy number assumptions used in absolute abundance and POC comparisons .
7) Clarify the statistical framework for physiological comparisons (grouping letters, pairwise tests, multiple-comparison adjustments) and specify exact n for each bar/point .
8) Rephrase the "no hot-bunking" claim to reflect the evidence basis (no diel oscillation observed in sampled light-reaction proteins) and acknowledge alternative explanations .

Minor/editorial comments
- Standardize gene/protein names and correct minor typographical issues (e.g., "phytotrasferrin" → "phytotransferrin"; "multicoper" → "multicopper") .
- Where the manuscript refers to "Extended Data Fig. 9/10" for key results (e.g., flavodoxin/ferredoxin), consider duplicating critical panels into the main figures for visibility .
- Ensure all acronyms are defined at first use in the main text and figure captions (e.g., DFOB, RND, TMM) .
- In Fig. 3c, consider reporting PSI:PSII ratio statistics (slope/confidence intervals), not only visual regression .

Version 1:

Reviewer comments:

Reviewer #1

(Remarks to the Author)
The authors have carefully and satisfactorily addressed all the comments raised in the previous round. The revisions have clearly improved the manuscript. I have no further comments and I support acceptance.

Reviewer #3

(Remarks to the Author)
The authors have satisfactorily addressed all the comments raised in the previous review. The revisions have improved the clarity, methodological soundness, and alignment between results and conclusions. The methodology is now described in sufficient detail to support reproducibility, and the analysis appropriately supports the claims made. Overall, the manuscript meets the expected standards of the field and represents a solid and relevant contribution to the literature.

Open Access This Peer Review File is licensed under a Creative Commons Attribution 4.0 International License, which permits use, sharing, adaptation, distribution and reproduction in any medium or format, as long as you give appropriate credit to the original author(s) and the source, provide a link to the Creative Commons license, and indicate if changes were made.
In cases where reviewers are anonymous, credit should be given to 'Anonymous Referee' and the source.
The images or other third party material in this Peer Review File are included in the article's Creative Commons license, unless indicated otherwise in a credit line to the material. If material is not included in the article's Creative Commons license and your intended use is not permitted by statutory regulation or exceeds the permitted use, you will need to obtain permission directly from the copyright holder.
To view a copy of this license, visit https://creativecommons.org/licenses/by/4.0/

When resubmitting, you must provide a point-by-point response to the reviewers' comments. Please show all changes in the manuscript text file with track changes or colour highlighting. If you are unable to address specific reviewer requests or find any points invalid, please explain why in the point-by-point response.

REVIEWER COMMENTS

Reviewer #1 (Remarks to the Author):

This manuscript investigates the adaptive strategies employed by the marine alga Pelagomonas calceolata, one of the most abundant eukaryotic phytoplankton in the oceans, under simultaneous iron and light limitation. The authors cultured P. calceolata under factorial combinations of iron availability and light intensity in laboratory conditions to characterize its physiological and transcriptomic responses. The results demonstrate that P. calceolata can maintain growth at extremely low cellular iron quotas through the dynamic deployment of iron-sparing mechanisms. Moreover, by comparing these laboratory-based findings with environmental metatranscriptomic data from natural communities, the study reveals that Fe- and light-responsive genes are also highly expressed in situ, underscoring the ecological relevance of these responses. Collectively, the data suggest that P. calceolata is highly specialized for environments where both iron and light are limiting, emphasizing its potential role in marine carbon and nitrogen cycling under such conditions. The manuscript is generally well-written and logically structured. However, certain aspects could benefit from further elaboration or clarification.

Below are specific suggestions:
1.Evidence for Fe/light co-limitation: Although the study is designed to simulate both iron and light limitation, further discussion on the combined effects of these two stressors, as opposed to their individual effects, would be valuable. For instance, is there a statistical interaction between iron supply and light intensity, indicating that low light exacerbates iron stress? Does the response of P. calceolata under simultaneous iron and light limitation exceed the additive effects of single-factor limitations? Additional analysis or discussion on the potential synergistic effects of iron–light co-limitation would strengthen the claim that this alga is specifically adapted to environments characterized by both constraints.

We agree that the combined effects of Fe and light warrant explicit analysis. We therefore quantified Fe × Light interactions across physiology, RNA, and proteomics using factorial models (see Methods, Line 575-585, 733-743, 789-801). In all cases the interaction term

estimates the excess over additivity of the co-limited state (LL & -Fe) relative to the sum of the single limitations (LL at +Fe and HL at -Fe). Across endpoints, significant interactions were a minority of features, and among those, antagonistic (negative) interactions were more common than synergistic (positive) in RNA and protein (see Supplementary Table 1 and Data 10-12). These patterns indicate that P. calceolata's Fe and low-light acclimations are largely additive, with occasional buffering under co-limitation, rather than broadly synergistic exacerbation. We interpret this as evidence of adaptation to co-limited niches and maintaining function at low Fe and low light without requiring strong synergistic responses. We have added to the Results (Line 177-189) and clarified the interpretation in the Discussion (Line 423-432).

2. Interpretation of gene-specific responses: The upregulation of RND family transporters under iron limitation is interpreted as a mechanism for exporting non-iron metal ions. While this is a plausible hypothesis, it would be more convincing if supported by direct measurements of other metal quotas or transport activities. If possible, providing such supporting evidence would enhance the robustness of this interpretation.

We agree that a specific role cannot be assigned from expression alone. We therefore revised the text to present RND upregulation as a hypothesis consistent with our measurements rather than a definitive mechanism. Specifically, under low Fe we observe increased cellular Cu and Cu:C that return toward +Fe values after Fe resupply. This covariation with RND transcripts is consistent with a role in non-Fe metal homeostasis (e.g., efflux), but confirmation will require future work that directly characterizes RND transporter function. We now state this and highlight targeted transport assays as a possibility for future work (Line 250-251).

3. Ecological success of P. calceolata: Although the data show that P. calceolata can maintain low cellular iron content and adjust gene expression under Fe/light stress, these traits alone may not fully explain its ecological dominance. Other factors not measured in this study—such as small cell size (and therefore high surface-area-to-volume ratio), reduced predation pressure, or the ability to utilize organic nutrients—could also contribute. If the species' ecological success is attributed mainly to its low-iron tolerance, this may oversimplify the complexity of its adaptive strategies. Additional discussion on these potential contributing factors, such as light quality, phosphorus availability etc.. or comparisons with other phytoplankton, would be valuable. Furthermore, labeling P. calceolata as a "co-limitation specialist" would be better supported by evidence of its competitive growth advantage under low Fe/low light conditions relative to other species, or at least by demonstrating sustained net growth under such conditions. While the data

show survival under Fe limitation, they also reveal clear growth deceleration (e.g., decreased carbon quotas and requirement for Fe re-supplementation for recovery). The manuscript should elaborate on how such traits translate into competitive advantage.

We agree that ecological success is multifactorial. In the revision we broaden the discussion to incorporate additional contributing factors (cell size/SA:V, organic nutrient access, and trophic interactions; Line 382-387) and temper language that could over-attribute dominance to Fe economy. We remove the term "co-limitation specialist" in reference to P. calceolata (Line 433), and state that P. calceolata is well adapted to Fe- and light-limited SCMLs. Our data show sustained net growth at low Fe with coordinated molecular acclimation, but we refrain from inferring comparative superiority; we now frame competitive advantage under co-limitation as a testable prediction for future studies (Line 271-274, 430-432).

4.Diurnal gene expression patterns: The observation that a large proportion of genes exhibit diurnal variation is a highlight point. However, the discussion lacks detail on whether P. calceolata possesses robust circadian regulation or whether Fe/light stress disrupts its diel rhythms. While nearly half of the transcriptome appears to be under diurnal control, only 14% of proteins show diel differences, suggesting regulation at the post-transcriptional level or other mechanisms. Although diel regulation is briefly referenced in the introduction, it is not revisited in the discussion. A more in-depth analysis could better showcase this novel aspect of the study.

We appreciate this suggestion. In response, we expanded our analysis and discussion of diel regulation. Using weighted gene co-expression network analysis (WGCNA), we identified gene modules whose expression patterns were significantly correlated with diel variables (sin24 hr, cos24 hr, and day/night sample categorization), indicating coordinated transcriptional programs that follow the daily light-dark cycle (Line 152-163, Figure 2d, 2e, and Supplementary Figures 4 and 5). Several of these diel modules were also correlated with Fe and light treatments, revealing that Fe limitation and light intensity modulate diurnal expression rhythms. This suggests that P. calceolata maintains a diel regulatory framework, but that environmental stress influences the phase and amplitude of specific gene networks, particularly those related to photosynthesis, carbon metabolism, and nutrient assimilation. Although much of the transcriptome exhibits diel variation, only a small number of proteins showed corresponding oscillations. The persistence of strong rhythmicity at the mRNA level but not in total protein abundance suggests that diel organization in P. calceolata operates mainly through transcriptional control, and changes

in the total protein pool are attenuated. We now discuss this in the revised manuscript as evidence that diel organization in P. calceolata is maintained at the transcript level but buffered at the protein level (Line 399-403). These analyses emphasize that diel gene expression remains a central organizing feature of P. calceolata physiology, but its expression networks are differentially modulated by Fe and light.

Minor comments:

1.The experimental design is central to the study's conclusions and should be described in more detail at the start of results section, rather than being confined to the Methods section.

Added description of experimental design to beginning of results section (Line 98-111).

2.how the two light intensities used were defined as "high light" and "low light"—are they based on natural conditions, or were they chosen based on light-response curve measurements?

The low light level (20 μmol quanta m^{-2} s^{-1}) was chosen to approximate irradiance at SCML depths where P. calceolata is abundant, based on in situ measurements of daily PAR at nitracline depths, which range from 10.9-26.4 μmol quanta m^{-2} s^{-1} in SCMLs (excluding deep photoacclimation zones) (Cornec et al. 2021). *The high light level (165 μmol quanta m^{-2} s^{-1}) was chosen to represent light-saturated conditions, based on a prior physiological study that demonstrated the growth rate of P. calceolata to saturate by 120 μmol quanta m^{-2} s^{-1}* (Timmermans et al. 2005). *Together, these levels bracket the natural irradiance range experienced by typical P. calceolata populations.*

3.In Figure 1C, why does the Fv/Fm value in the "+Fe" group continuously decline?

We appreciate the question. In the +Fe treatments, Fv/Fm shows a modest decline that becomes statistically significant only at the end of the experiment. We do not ascribe a specific cause, and importantly this trend was not accompanied by detectable changes in other physiological measurements over the same interval. As a cautionary step, we excluded the final LL +Fe timepoint (23hr) from the proteomics analyses. The corresponding RNA-seq samples were retained and clustered with the earlier +Fe transcriptomes, indicating comparable cellular state and leaving our conclusions unchanged.

4.The claim that P. calceolata may perform the majority of nitrate assimilation in SCML

communities is based on transcriptomic and proteomic data. This conclusion would be strengthened by physiological measurements.(if possible)

We agree that direct physiological nitrate uptake measurements would further strengthen this conclusion. Our statement that P. calceolata is a major nitrate assimilator in SCML communities is grounded primarily in previous in situ work, which our study reinforces mechanistically. In particular, a 2014 study (Dupont et al. 2014) *showed that pelagophytes with transcriptomes highly similar to Pelagomonas sp. CCMP1756 dominated nitrate assimilation gene expression in SCML communities, contributing >90% of all nitrate transporter transcripts across their transect and expressing a full suite of nitrate/nitrite transport and assimilation genes at high levels. They concluded that these pelagophytes "may dominate nitrate uptake and reduction" in the SCM and highlighted them as keystone picoeukaryotic phytoplankton. Our study does not add new in situ rate measurements, but it strengthens this picture by showing that the P. calceolata genome encodes the complete nitrate assimilation machinery, that nitrate/nitrite transporters and downstream enzymes are strongly expressed and regulated in our cultures under SCML-like Fe/light conditions, and that the same Fe- and light-responsive assimilation genes are highly expressed in SCML metatranscriptomes from the California Current. Together, these observations support a central role for P. calceolata in nitrate assimilation within SCML communities.*

In light of the reviewer's comment, we have softened the wording in the manuscript to emphasize that (i) the dominance of nitrate assimilation by P. calceolata is inferred primarily from transcriptomic evidence (ours and Dupont et al.), (ii) our results provide mechanistic support for that inference, and (iii) direct measurements of P. calceolata nitrate uptake in SCML conditions remain an important target for future work (Line 356-359).

5.Growth monitoring was based on RFU. Since pigment content can vary under different light and iron conditions, this could affect the accuracy of the growth estimation?

RFU measurements were used only to track pre-culture growth and confirm exponential phase at the start of the experiment; quantitative comparisons among treatments were based exclusively on flow cytometric cell counts.

6.Some references to supplementary materials appear as "Supplementary Table XX." These should be corrected.

These supplemental materials have been properly referenced.

7.Ensure that all abbreviations, including gene and protein names, are defined upon first use.

Abbreviations, gene names and proteins names have been defined.

8.The numbering of Extended Data Figures should follow their order of appearance in the text (e.g., Figure S9 is referenced before Figure S5).
Numbering of Extended Data Figures (now Supplementary Figures) has been updated to reflect this change.

Reviewer #2 (Remarks to the Author):

I read with interest the manuscript titled “Molecular and Physiological Acclimation to Low Light and Iron Scarcity in Globally Abundant Oceanic Pelagophyte”. This paper investigates the strategies of Pelagomonas calceolata to iron and light co-limitation, through physiological and gene expression analysis. The findings of the paper are well written and the analyses are robust and appropriate. Overall, this paper makes an important contribution to our understanding of algal biology.

With expertise in genome assembly, I have limited my comments to this aspect of the paper. The methods used are appropriate and well reported to aid reproducibility. Additionally:

L150 – Supplementary Table XX?

These supplemental materials have been properly referenced.

L385 – It would be good to detail the DNA extraction method thoroughly here, similar to the detail for RNA extraction at L455.

The DNA extraction protocol has been detailed in the methods (Line 469-479).

L397 – I can’t seem to find Figure S12 and 13. For me, the extended data Figures end at Extended Data Fig.10.
Extended Data Figures (now Supplementary Figures) have been updated and numbered properly.

Reviewer #3 (Remarks to the Author):

- This manuscript presents an integrated physiological, transcriptomic, proteomic, and environmental analysis of Pelagomonas calceolata under iron (Fe) and light limitation, including short-term Fe resupply dynamics over a diel cycle. It reports exceptionally low Fe:C ratios, rapid Fe-sparing responses (e.g., flavodoxin for ferredoxin), distinct remodeling of Calvin-Benson cycle components under Fe stress, shifts in nitrogen source preference, and concordance between laboratory Fe/light response signatures and in situ metatranscriptomes from SCML communities in the California Current Ecosystem (CCE). Elemental quotas (C, N, Fe, Cu) and Chl a, together with diel RNA-seq and TMT proteomics, are used to support these claims.

- P. calceolata maintains low Fe:C ratios across treatments and rapidly increases Fe:C within hours of resupply due to fast Fe uptake relative to C restoration, demonstrating stringent Fe economy under both high and low light. These trends are supported by measured C, N, Fe quotas and derived Fe:C across time points.
- Under low Fe, Cu quotas and Cu:C increase, opposite to Fe trends, with Cu returning toward Fe-replete values after Fe resupply; authors interpret this as potential increased reliance on Cu proteins and/or non-specific metal uptake under Fe stress. Moreover, transcriptional responses are strongly structured by day/night and Fe availability; proteomic remodelling is driven more by Fe and light, with less diel variation than the transcriptome (43.9% of transcripts vs 14.3% of proteins differentially expressed by day/night) .
- Canonical Fe-sparing: flavodoxin is upregulated in low Fe (daytime-biased at the transcript level), replacing ferredoxin at the protein level; post-resupply, flavodoxin rapidly declines while ferredoxin increases .
- Fe acquisition machinery: phytotransferrin (pTF) and ISIP3 are upregulated at low Fe at both transcript and protein levels; a ferrichrome-like siderophore receptor is induced under low Fe and decreases upon resupply, consistent with capacity to access strong Fe ligands; ZIP transporters are more expressed in Fe replete cultures, suggesting intracellular roles beyond Fe uptake; RND family transporters are upregulated in low Fe and may mediate efflux of non-Fe metals .
- Photosynthesis and Calvin-Benson cycle: light-harvesting and PSI/PSII reaction center proteins increase in low light with Fe present; PSI/PSII balance is maintained across treatments; a switch from Class II to Class I fructose-bisphosphate aldolase (FBA), plus coordinated low-Fe expression of Fbp and Rpe, indicates rewiring of the regenerative phase under Fe stress .

- P. calceolata is abundant in the CCE (often millions of cells per liter) and frequently contributes more POC than Prochlorococcus or Synechococcus; genes identified as Fe- and light-responsive in culture are enriched in SCML metatranscriptomes, supporting in situ expression of these adaptations.

- The study advances mechanistic understanding of how a globally distributed picoeukaryote thrives at Fe/light co-limited SCMLs, linking cellular Fe economy, diel regulation, and N source flexibility to environmental niches and biogeochemical roles. The integration of controlled diel-resolved culture work with field metatranscriptomes at scale is a notable strength. While Fe-sparing modules (flavodoxin, pTF/ISIPs) and light effects on Fe demand are known in phytoplankton, this work uniquely characterizes their coordinated, time-resolved deployment in P. calceolata under co-limitation and validates environmental relevance in the CCE. The observed switch in Calvin-Benson components (Class I vs II FBA, Fbp, Rpe) and the Fe-driven shift in N source preference in this taxon add new detail to eukaryotic picoalgal acclimation strategies .
- The manuscript situates results within prior observations of low Fe requirements in P. calceolata, Fe/light interactions in phytoplankton, and canonical Fe-stress machinery across taxa; it highlights differences from diatom models (e.g., urea cycle regulation, ferritin absence) and from diazotroph "hot-bunking," arguing P. calceolata prioritizes Fe sparing over storage .

The data support the conclusions since the low Fe:C and rapid Fe resupply responses in quotas and Fv/Fm are well documented across replicates and time points. Fe-sparing via flavodoxin-for-ferredoxin substitution and induction of Fe acquisition pathways (pTF, ISIP3) are robust at both transcript and protein levels and revert with Fe addition . Downregulation of nitrate/nitrite reduction proteins at low Fe and upregulation of urea transporter/urease supports a plausible shift in N source usage under Fe stress .

I would highly suggest some revision to temper claims or add evidence in the lack of plastocyanin-for-cytochrome c6 substitution: evidence is mostly from transcript patterns (both upregulated in high Fe) and non-detection of plastocyanin in proteomes; given possible limits of proteome coverage, more direct quantification (e.g., targeted proteomics) would better support this negative result .

Moreover there is "No evidence of Fe hot-bunking": the absence of pronounced diel oscillation in light-reaction proteins suggests stable investment, but does not strictly

exclude nighttime Fe recycling; this conclusion should be framed cautiously as “no evidence observed in our proteome time points” .

Dynein upregulation indicates a role under low Fe/high light but functional assignment to motility or osmotrophy remains inferential; additional phenotyping (e.g., flagellar motility assays) would strengthen this link.

Siderophore uptake inference from ferrichrome-like receptor expression and growth under DFOB is suggestive; direct uptake assays or radiotracer experiments with defined siderophores would make this claim more definitive .

Methodology and analysis: strengths and points needing clarification

- Strengths:
- Clean trace-metal culturing in Aquil with explicit Fe speciation calculations (Fe') and chelator perturbations; acclimation for >10 generations prior to experiments; diel sampling design with biological triplicates under two light regimes .
- Quantitative elemental quotas (CHN analyzer; ICP-MS with oxalate-EDTA rinse) and chlorophyll by standard fluorometry provide solid physiological grounding .
- Transcriptomics: ribodepletion, consistent library prep, edgeR GLM across factors; clear day/night and Fe/light separation in PCA/UMAP; response-type assignments are systematic .
- Proteomics: TMT multiplexing, rigorous filtering, normalization (including TMM), and edgeR downstream statistics; alignment to a curated genome assembly and gene models of P. calceolata .
- Environmental mapping: taxonomic assignment via PhyloDB, DIAMOND mapping to the genome, normalization with DESeq2; quantitative 18S-based cell abundance estimates anchored to internal standards .

The paper needs more clarifications on the multiple testing control:

The manuscript refers to $p<0.05$ thresholds in edgeR without explicitly stating FDR procedures for transcript or protein DE; please report FDR-adjusted criteria and the number of DE features under specified FDR (e.g., 5%) for each contrast

Proteomics data handling: Seven low-abundance samples were removed and missing values imputed at half row-minimum prior to edgeR. Please demonstrate robustness of key conclusions to alternative imputation strategies and to inclusion/exclusion of these

samples (sensitivity analysis), and justify any potential biases introduced by the filtering step .

Siderophore/DFOB inferences: You calculate Fe' ~5.6 fM with 100 nM DFOB and infer uptake of strongly bound Fe; consider including direct growth rate comparisons and/or uptake assays to substantiate siderophore-based acquisition beyond expression profiles .

Plastocyanin vs c6: Given non-detection can be due to proteomic coverage, targeted quantification (PRM/SRM) or spectral counting statistics would clarify whether plastocyanin is truly absent at low Fe/high Cu, or below detection.

Metatranscriptome mapping specificity: Cross-mapping among pelagophytes can inflate apparent P. calceolata signal. Please provide mapping stringency, unique vs multi-hit handling, and estimates of potential cross-mapping. A sensitivity test restricting to diagnostic genes would increase confidence in the environmental enrichment results .

POC contribution estimates: Field C per cell is inferred from the average of culture quotas (104.3 fmol C $cell^{-1}$) applied across diverse environments; please include uncertainty ranges (e.g., based on condition-specific quotas from your experiments) and explore how SCML vs surface conditions alter the estimates .

18S copy number assumption: The conversion to cells assumes two 18S copies per cell; discuss potential copy-number variability among wild P. calceolata clades and its impact on absolute abundance estimates (provide sensitivity bounds).

Statistical reporting for physiological comparisons: Extended Data figures show letters for groupings and Welch's t-tests for some pairwise contrasts. Please specify the full multiple-comparison framework used (e.g., ANOVA with post-hoc tests), correction for multiple testing, and exact n for each comparison.

- Methods are generally detailed and meet field standards for trace-metal culture, sequencing, and proteomics, with sufficient detail to replicate culturing, physiological measurements, and sequencing workflows . The Fe speciation calculation parameters are given; providing the exact computation workflow (spreadsheet or code) would further enhance reproducibility .

I would hihly recommend providing the scripts or detailed parameter files (e.g., edgeR model formulas and contrasts, normalization steps, imputation code) to fully reproduce the RNA-seq and proteomics statistical analyses .

- I do not see fatal flaws. The core experimental design and multi-omics integration are strong. However, the missing supplementary items/data access and some analytical clarifications need to be addressed before acceptance. A moderate revision focusing on:
- Completing and depositing all supplementary figures/tables/data,
- Clarifying statistical controls and robustness of proteomics inferences,
- Tempering or reinforcing a few mechanistic claims with additional analysis,
would make the manuscript publication-ready .

Specific requests for revision
1) Provide complete supplementary materials (all referenced Extended Data Figs 8–10 and finalized Supplementary Tables) and update data availability with proteomics repository accessions.

Supplementary materials have been updated, and proteomics repository accessions have been added.

2) Detail multiple testing corrections (FDR) for all DE analyses (transcript and protein); report thresholds and DE counts per contrast; include MA/volcano plots per major contrast in the supplementary material.

We have revised the manuscript to clarify and standardize multiple-testing correction across all differential-expression analyses. For the transcriptomic data, we now explicitly state that edgeR was used with Benjamini–Hochberg false discovery rate (FDR) control at FDR = 0.05, and we report the number of differentially expressed transcripts per contrast (Line 564-575, Supplementary Figure 3b,c, Supplementary Data 15). For the proteomic data, we re-ran the differential analysis using limma(Ritchie et al. 2015) *on the normalized/imputed protein-intensity matrix, applying empirical Bayes moderation and Benjamini–Hochberg FDR correction (FDR = 0.05) (Line 721-726). In addition, we added volcano plots for the main proteomic comparisons discussed in the text (light-level comparisons at pFe and mFe, Fe-level comparisons within light, and day–night comparisons, including DE counts per contrast) to the Supplementary Material (Supplementary Figure 6-8). These revisions address the request to detail multiple-testing procedures and to visualize DE results.*

3) Proteomics robustness: include sensitivity analyses for imputation/filtering choices; report peptide counts/coverage for plastocyanin and cytochrome c6 to support the "no substitution" inference or temper the claim.

Thank you for this question. We removed certain samples from the proteome dataset before normalization and quantification because they had greatly reduced protein identification numbers and elevated missing values. Inclusion of these samples would have greatly limited the number of proteins we were able to quantify across the full dataset. To ensure that filtering and imputation did not introduce bias, we have compared quant values for proteins with and without filtering and for 4 different imputation strategies (Line 711-720). Supplemental Data 15 shows the impact of filtering and imputation on protein expression values for 14 key proteins discussed in the manuscript.

Sensitivity testing of filtering and imputation on proteomic results

To test the effect of filtering and imputation on our proteome results we compared protein abundance values, generated in the TMT experiment, with different filtering, imputation strategies prior to normalization. We added three additional imputation strategies to the one originally reported in the manuscript. The methods compared are as follows,

'norm' – all rows with missing values removed and standard normalization procedure outlined in methods applied

'filt1' - 6 samples removed, normalization

'filt2' - 7 samples removed, normalization

*'filt2_imp' - 7 samples removed, impute 1/2 min, normalization *(Values reported in paper)*

'filt2_impseq' - 7 samples removed, impute using sequential imputation, normalization

'filt2_impseqrob' - 7 samples removed, impute using robust sequential imputation, normalization

'filt2_grr' - 7 samples removed, impute using Glmnet Ridge Regression (GRR), normalization

Alternate imputation strategies were performed using NAguideR and the best scoring methods were selected https://wkomics.omicsolution.com/wukong/NAguideR/. The methods applied were, robust sequential imputation (impseqrob) https://doi.org/10.1016/j.compbiolchem.2008.07.019, sequential imputation (impseq) https://doi.org/10.1016/j.compbiolchem.2007.07.001, Glmnet Ridge Regression (GRR) https://github.com/WangLab-MSSM/DreamAI.

Following filtering of samples with low identification rates and imputation, results were normalized as outlined in the methods.

These results demonstrate that filtering alone, without imputation or more advanced imputation methods such as robust sequential imputation (impseqrob), sequential imputation (impseq), Glmnet Ridge Regression (GRR) do not greatly affect the derived protein expression values or their relative expression across treatments.

"Plastocyanin vs c6: Given non-detection can be due to proteomic coverage, targeted quantification (PRM/SRM) or spectral counting statistics would clarify whether plastocyanin is truly absent at low Fe/high Cu, or below detection."

We confirmed that no plastocyanin peptides were detected in the TMT-proteomics experiment. While performing SRM or label free proteomics on the latest state of the art mass spectrometers is a good idea, not enough sample for all treatments remains for such an analysis. We agree that such proteins may have been detected with different proteomic approaches.

4) Siderophore uptake: either add a brief growth/uptake validation under DFOB or explicitly frame this as a hypothesis consistent with expression patterns rather than a demonstrated capability.

We have added an experimental validation confirming Fe–DFOB uptake using a ^{59}Fe radiotracer assay. Cultures of P. calceolata (+Fe and –Fe) and P. tricornutum (–Fe) were incubated with ^{59}Fe–desferrioxamine B, and intracellular radioactivity was measured by liquid scintillation counting over an 80-min time course. Uptake rates (amol Fe cell^{-1} min^{-1}) were derived from the linear increase in ^{59}Fe accumulation per cell. P. calceolata showed measurable Fe–DFOB uptake, with elevated rates under Fe limitation, while P. tricornutum (known to access Fe-DFOB via an ortholog of the siderophore binding protein discussed in the text (Coale et al. 2019)*) exhibited substantially higher rates presumably due to larger cell size. These data provide direct evidence that P. calceolata can utilize siderophore-bound iron, validating the transcriptomic inference of siderophore uptake capability (Line 234-236, 238-240, 852-875, Supplementary Figure 12).*

5) Environmental mapping specificity: report unique mapping rates, handling of multi-mappers, and include a conservative analysis based on diagnostic gene sets to confirm SCML enrichment patterns .

For a metatranscriptome read to be classified as P. calceolata in our data, it must map to an assembled open reading frame (ORF) annotated as P. calceolata. Therefore, both the

taxonomic annotation of ORFs and read mapping need to be considered. To avoid mis-annotations, taxonomic assignment of metatranscriptome ORFs was based on BLAST results to our reference database, PhyloDB, and the lineage probability index (LPI) as described by Podell and Gaasterland (2007)(Podell and Gaasterland 2007). *PhyloDB includes 11 other pelagophyte reference organisms, and the average LPI score for P. calceolata ORFs here was 96% indicating that we can have high confidence in our assignment of ORFs as P. calceolata.*

Read mapping was performed with CLC mapper within CLC Assembly Cell v5.2.1.216695-202010091408 using paired end reads and the default settings. With the default settings, reads with multiple equal-scoring hits are randomly assigned, and therefore, they are not double counted. Across all reads regardless of their taxonomic assignment, the average multi-mapping rate was 9.9%; therefore, multi-mapped reads only represent a relatively small fraction of the overall counted reads. Although these reads may include multi-mapped reads between P. calceolata and other organisms, they may also include multi-mappings within P. calceolata.

Furthermore, P. calceolata represents 64% and 71% of all Pelagophyceae reads with our 18S rRNA gene sequencing for the V4 and V9 regions respectively, indicating that it dominates the pelagophyte community in these samples. This dominance reduces the probability that a read from a different organism is mis-counted as P. calceolata. These details were added to the methods (Line 833-850)

6) POC estimates and 18S copy number: add uncertainty bounds/sensitivity analyses for C quotas and rDNA copy number assumptions used in absolute abundance and POC comparisons.

We have added a sensitivity analysis to quantify the effects of uncertainty in cellular carbon quotas and rDNA copy number on estimated particulate organic carbon (POC) contributions by P. calceolata (Line 374-378, 812-832, Supplementary Figure 16). Specifically, we recalculated P. calceolata:cyanobacteria carbon ratios across a range of plausible cellular carbon contents (low-high quotas derived from culture measurements) and genome ploidy levels (1-8 genomes per cell, assuming two 18S rDNA copies per genome). The resulting distributions are shown in Supplementary Figure 16, which illustrates how these assumptions influence the inferred relative carbon contributions of P. calceolata compared with Prochlorococcus and Synechococcus. Percentages above each violin plot indicate the fraction of samples where P. calceolata carbon exceeded that of the corresponding cyanobacterium. While absolute ratios vary with the assumed parameters (especially ploidy), the overall conclusion that P. calceolata represents a substantial

contributor to POC, particularly in subsurface chlorophyll maximum layers, remains robust across all tested scenarios.

7) Clarify the statistical framework for physiological comparisons (grouping letters, pairwise tests, multiple-comparison adjustments) and specify exact n for each bar/point .

We have expanded the Methods section to describe the full statistical framework used for the physiological comparisons (Line 776-801). Each parameter was analyzed using pairwise Welch's t-tests among Fe treatments within a given light level and timepoint, with P-values adjusted for multiple comparisons using the Holm method. Compact letter displays were used to indicate groups that were not significantly different ($\alpha = 0.05$), and pairwise Welch's t-tests between HL and LL treatments were performed separately to identify significant light effects (denoted by asterisks in the figures). These details are now included in the revised Methods section ("Statistical analysis of physiological measurements"). Exact n values (biological triplicates; $n = 3$ per treatment) are now explicitly stated in the figure caption and described in the Methods text.

8) Rephrase the "no hot-bunking" claim to reflect the evidence basis (no diel oscillation observed in sampled light-reaction proteins) and acknowledge alternative explanations .

We have revised the text to clarify that our conclusion is based on the observed lack of diel oscillation in light-reaction protein abundances, rather than the complete absence of Fe recycling, and acknowledges potential alternative explanations (Line 399-403).

Minor/editorial comments

- Standardize gene/protein names and correct minor typographical issues (e.g., "phytotrasferrin" → "phytotransferrin"; "multicoper" → "multicopper") .

Done

- Where the manuscript refers to "Extended Data Fig. 9/10" for key results (e.g., flavodoxin/ferredoxin), consider duplicating critical panels into the main figures for visibility.

We have added a panel of flavodoxin gene expression to the main text figures (Figure 2b). Flavodoxin and ferredoxin protein/gene expression are also presented in Figure 5.

- Ensure all acronyms are defined at first use in the main text and figure captions (e.g., DFOB, RND, TMM).

Acronyms defined.

- In Fig. 3c, consider reporting PSI:PSII ratio statistics (slope/confidence intervals), not only visual regression.

We have updated this figure with confidence intervals and slope.

References

Coale, Tyler H., Mark Andrew Moosburner, Aleš Horák, Miroslav Oborník, Katherine A. Barbeau, and Andrew E. Allen. 2019. "Reduction-Dependent Siderophore Assimilation in a Model Pennate Diatom." Proceedings of the National Academy of Sciences 116(47): 23609–17. doi:10.1073/pnas.1907234116.

Cornec, M., H. Claustre, A. Mignot, L. Guidi, L. Lacour, A. Poteau, F. D'Ortenzio, B. Gentili, and C. Schmechtig. 2021. "Deep Chlorophyll Maxima in the Global Ocean: Occurrences, Drivers and Characteristics." Global Biogeochemical Cycles 35(4): e2020GB006759. doi:10.1029/2020GB006759.

Dupont, Christopher L., John P. McCrow, Ruben E. Valas, Ahmed Moustafa, Nathan G Walworth, Ursula Goodenough, Robyn Roth, et al. 2014. "Genomes and Gene Expression across Light and Productivity Gradients in Eastern Subtropical Pacific Microbial Communities." The ISME Journal 9(5): 1076–92. doi:10.1038/ismej.2014.198.

Podell, Sheila, and Terry Gaasterland. 2007. "DarkHorse: A Method for Genome-Wide Prediction of Horizontal Gene Transfer." Genome biology 8(2): R16.

Ritchie, Matthew E, Belinda Phipson, DI Wu, Yifang Hu, Charity W Law, Wei Shi, and Gordon K Smyth. 2015. "Limma Powers Differential Expression Analyses for RNA-Sequencing and Microarray Studies." Nucleic acids research 43(7): e47–e47.

Timmermans, Klaas R., B. Van Der Wagt, Marcel J.W. Veldhuis, a. Maatman, and Hein J.W. de Baar. 2005. "Physiological Responses of Three Species of Marine Pico-Phytoplankton to Ammonium, Phosphate, Iron and Light Limitation." Journal of Sea Research 53: 109–20. doi:10.1016/j.seares.2004.05.003.
